# Long non-coding RNAs as pan-cancer master gene regulators of associated protein-coding genes: a systems biology approach

**DOI:** 10.7717/peerj.6388

**Published:** 2019-02-20

**Authors:** Asanigari Saleembhasha, Seema Mishra

**Affiliations:** Department of Biochemistry, School of Life Sciences, University of Hyderabad, Hyderabad, Telangana, India

**Keywords:** *PVT1*, E2F1, FOXM1, RNAseq, TCGA, NETWORK, Long non-coding RNAs, Pan-cancer

## Abstract

Despite years of research, we are still unraveling crucial stages of gene expression regulation in cancer. On the basis of major biological hallmarks, we hypothesized that there must be a uniform gene expression pattern and regulation across cancer types. Among non-coding genes, long non-coding RNAs (lncRNAs) are emerging as key gene regulators playing powerful roles in cancer. Using TCGA RNAseq data, we analyzed coding (mRNA) and non-coding (lncRNA) gene expression across 15 and 9 common cancer types, respectively. 70 significantly differentially expressed genes common to all 15 cancer types were enlisted. Correlating with protein expression levels from Human Protein Atlas, we observed 34 positively correlated gene sets which are enriched in gene expression, transcription from RNA Pol-II, regulation of transcription and mitotic cell cycle biological processes. Further, 24 lncRNAs were among common significantly differentially expressed non-coding genes. Using guilt-by-association method, we predicted lncRNAs to be involved in same biological processes. Combining RNA-RNA interaction prediction and transcription regulatory networks, we identified E2F1, FOXM1 and *PVT1* regulatory path as recurring pan-cancer regulatory entity. *PVT1* is predicted to interact with *SYNE1* at 3′-UTR; *DNAJC9, RNPS1* at 5′-UTR and *ATXN2L, ALAD, FOXM1* and *IRAK1* at CDS sites. The key findings are that through E2F1, FOXM1 and *PVT1* regulatory axis and possible interactions with different coding genes, *PVT1* may be playing a prominent role in pan-cancer development and progression.

## Significance

Our paper delves on the role and interplay of coding and long non-coding genes in almost all cancer types which are characterized by same biological hallmarks using systems biology. Analyzing the interplay, we have zeroed in on E2F1, FOXM1 (transcription factors) and *PVT1* (lncRNA) regulatory path as recurring pan-cancer regulatory entity. E2F1 expression may be indirectly regulated by *PVT1*, with *ETS1* being a powerful mediator between the two coding and non-coding genes. In addition to providing novel cancer drug targets, our results provide key insights into the interplay of these emerging novel class of gene regulators.

## Introduction

Cancer, a hitherto-largely impregnable disease, is characterized by distinct hallmarks (Hanahan & Weinberg, 2011) which generalize their biological complexity. Transformation of normal cells into abnormal ones, i.e., cancer, is associated with profound changes in gene expression profile (Kopnin, 2000; Ismail et al., 2000; Herceg & Hainaut, 2007). Factors and causes involved range from genetic (somatic mutation and copy number variations) to epigenetic changes which in turn, lead to differential gene expression due to dysregulation (Sadikovic, Al-Romaih & Squire J.A, 2008; Du & Che, 2017). The dysregulated genes have been found to include both coding and noncoding RNAs (Ezkurdia et al., 2014; Yan et al., 2015). Among non-coding RNAs, long non-coding RNAs (LncRNAs), are found to be involved in epigenetic regulation, transcription and translation processes (Derrien et al., 2012; Mattick & Rinn, 2015; ENCODE Project Consortium et al., 2007; Mercer & Mattick, 2013; Patil et al., 2016). Several lncRNAs have been identified as a tumor suppressor or oncogene in most of the tumors and are aberrantly expressed in cancers (Yang et al., 2017b; Song et al., 2016b; Liu et al., 2016a; Chen et al., 2015; Pandey et al., 2014). Interacting with proteins and other RNAs, these may play an important role in signal transduction processes in cancer and normal cells in their capacity as signals, decoys, guides and scaffolds (Kung, Colognori & Lee, 2013). Many lncRNAs have been correlated with development and disease mainly due to the changes in their expression levels.

Studies are being carried out to understand their precise roles and molecular mechanisms of action. These act through diverse roles such as through down-regulation of gene expression at RNA level (Salameh et al., 2015), through Staufen 1 (STAU1)-mediated messenger RNA decay (SMD) (Gong & Maquat, 2011) and/or acting synergistically in regulating genes (Ma, Bajic & Zhang, 2013). LncRNAs have been demonstrated in several recent studies to be associated with cancer and role and mechanisms of several lncRNAs in cancer signaling pathways is detailed in a recent comprehensive review (Schmitt & Chang, 2016). Promoting cell proliferation in prostate cancer cells (Prensner et al., 2011), effects on the transcriptional and post-transcriptional regulation of cytoskeletal and extracellular matrix genes in lung adenocarcinoma cells (Tano et al., 2010), repressing tumor suppressors INK4a/p16 and INK4b/p15 (Yap et al., 2010; Kotake et al., 2011) are postulated to be some of the mechanisms. *HOTAIR* overexpression is associated with poor prognosis in several cancers and it has been suggested that it may increase tumor invasiveness and metastasis (Gupta et al., 2010). Thus, lncRNAs as master regulators have a strong potential to be used as biomarkers or drug targets. Several studies have been in place towards pan-cancer gene expression and lncRNA profiling (Bolha, Ravnik-Glavač & Glavač, 2017; Zhang et al., 2016; Wang et al., 2018), however, these studies have been performed separately for specific cancer types. An integrated approach towards identification of non-coding lncRNAs serving as likely master regulators of differentially expressed coding genes in cancer will fill the gap in our understanding of the regulatory role of these lncRNAs on mRNAs and proteins in cancer. Our studies will hopefully provide us with key novel molecule/s as common target/s involved in all the major and emerging hallmarks and possible one miracle drug intervention.

Using latest RNAseqV2 data from The Cancer Genome Atlas (TCGA), we performed several statistical analyses on global mRNA expression data from 15 types of cancers and lncRNA expression profiles from nine cancer types, these types are also present in above-mentioned 15 cancer types. Key findings from our studies have identified *E2F1, FOXM1,* and *ETS1* as coding gene/s axes that are overexpressed and may show strong involvement in tumor development in almost all cancer types. Several lncRNA/s such as *PVT1*, *SNHG-11*, *MIR22HG* and *UHRF-1* among others have been postulated to play a regulatory role for these protein-coding genes. It is intriguing to observe that the biological processes predicted for these lncRNA-mRNA modules fulfill all the six distinct cancer hallmarks. Validated by our systems-scale observations and literature evidence, we have attempted to put forward a working model of these genes’ regulation by lncRNAs that is uniform across cancer types.

## Materials and Methods

### Protein-coding genes (mRNA) analyses

#### Data acquisition

Gene expression data was obtained from TCGA data portal (https://cancergenome.nih.gov/ downloaded in the year 2016. We selected UNC_IlluminaHiseq_RNAseqV2 platform only to avoid heterogeneity of data and normalized level-3 sample data sets. A total number of samples was 5,601 from 15 different cancer types (primary cancers) and 601 corresponding normal samples. Each sample had associated expression values for 20,531 protein-coding genes.

#### Significant differential expression analysis of each gene

We analyzed significant differential expression by using unpaired *t*-test with unequal group variances (Welch approximation) in Multiple Experiment Viewer (MeV) version 4.9.0 (http://mev.tm4.org/) and Linear Model for Microarray (LIMMA) as a package in RStudio program (https://www.r-project.org/). For assessing significant differential expression in the *t*-test, the default cut-off was a critical *p*-value of 0.01 and in LIMMA, double filtration approach with the alpha value 0.01 and fold change >2 cut-offs was used. Combining these two results in order to increase accuracy and eliminate false positives, significantly differentially expressed common genes were enlisted using Venn diagram.

#### Identification of up- and down-regulated genes

Using our significantly differentially expressed common genes list, we applied Comparative Marker Selection version 10.1 (http://software.broadinstitute.org/cancer/software/genepattern/modules/docs/ComparativeMarkerSelection/10) to identify up- and down-regulated genes between the two classes of samples (cancer and normal) by *t*-test (median centered) at 10,000 permutations and a *p*-value cutoff of 0.01 with Bonferroni correction.

#### Correlation of protein and mRNA expression levels

We compared protein expression level (intensity obtained from immunohistochemistry results) published in “Human Protein Atlas (HPA)” (available from https://www.proteinatlas.org/, data accessed in 2016–2017) with mRNA expression level of each of the 70 significantly differentially expressed coding genes. As per Cancer Atlas in HPA, the staining intensity for each protein expression level from immunohistochemistry data is given in the range: high, medium, low and not detected, from the expert manual analysis. Side comparisons with normal samples are also presented.

#### Functional annotation

For functional annotation, we used GeneCoDis3 web server (http://genecodis.cnb.csic.es/). This web server takes a list of genes as inputs and annotates a gene set using Gene Ontology, KEGG Pathways, and other terms. Significance of annotations to gene set is determined by *p*-values computed using hypergeometric distribution or chi-square test. For our analyses, we examined only those annotations for which *p*-values obtained through hypergeometric analysis and corrected by the FDR method of Benjamini and Hochberg were provided for significance. Hypergeometric *p*-value of <or = 0.05 (default) was used. The results can be seen as the tags cloud where most significant terms that are enriched in the list of genes are presented as fonts of variable sizes. The font size of each tag depends on the number of genes enriched from our gene list that are present in a particular annotation.

### Non Protein-coding genes (Long Noncoding RNA (lncRNA)) analyses

#### Data acquisition

LncRNAtor database (http://lncrnator.ewha.ac.kr/expression.htm) is an online database which integrates expression profile, interacting protein, integrated sequence curation, evolutionary scores, and coding potential. TCGA, GEO, ENCODE, and modENCODE databases provide the relevant datasets. We selected differentially expressed lncRNAs from TCGA RNAseq platform, level-3 count data. Searching for the same cancer types as present in TCGA database, we found nine cancer types which were the same as the cancer types used in coding genes’ work and for which data from TCGA for comparing normal vs tumor was present. In this way, a total of 2,900 samples with 403 normal and 2,497 tumor samples were investigated. Total number of lncRNAs in lncRNAtor was 24,154. Significantly differentially expressed lncRNAs identified by the database authors using DESeq2 package for TCGA level 3 data with adj *p*-value 0.01 were enlisted from the database. Bioconductor software specifies about DESeq2 as follows: “It estimates variance-mean dependence in count data from high-throughput sequencing assays and tests for differential expression based on a model using the negative binomial distribution” ( Love, Huber & Anders, 2014). We then used Comparative Marker Selection version 10.1 to discriminate up- and down-regulated lncRNA genes between two classes of samples by *t*-test (median centered) at 10,000 permutations and a *p*-value cutoff of 0.01. We used lncRNAtor and GeneCodis3 to predict co-expressed mRNAs and their role in biological processes and molecular function in order to assign putative functions to uncharacterized lncRNAs by “guilt-by-association”.

#### Prediction of lncRNA-mRNA interaction and function

We used the human lncRNA-mRNA interaction database (http://rtools.cbrc.jp/cgi-bin/RNARNA/index.pl) (Terai et al., 2016) which is validated. It contains lncRNA-mRNA and lncRNA-lncRNA interactions between 23,898 lncRNAs and 20,185 mRNA (human) sequences obtained from the GENCODE Project (http://www.gencodegenes.org/releases/19.html). The first step in this pipeline; RACCESS program is used for extracting accessible regions in each RNA sequence, Step 2: tandem repeats are removed using the TANTAN program, Step 3: reverse complementary ‘seed matches’ are detected using LAST, Step 4: the binding energies between query and target RNAs from the seed matches are evaluated using INTARNA. In Step 5, the candidate interaction pairs are ranked according to the interaction energies (SUMENERGY and MINENERGY). Finally, the interaction site secondary structure with minimum interaction energy is predicted in each pair of RNA sequence using RACTIP. Using this database, we analyzed interactions of common significantly differentially expressed mRNAs and lncRNAs enlisted from our studies. Interaction energy (ranking was done using SUMENERGY score which is adequate for long RNA sequences such as lncRNAs and mRNAs), location of the interaction site on mRNA and joint secondary structures of the binding site were predicted. We used Excel for generating plots of SUMENERGY.

#### Integrated Network analysis

For experimentally verified transcriptional regulatory network (TFs-gene interaction) analysis, we retrieved data from Open-Access Repository of Transcriptional Interactions (ORTI, URL: http://orti.sydney.edu.au/about.html) database, which apart from other databases on mammalian transcription factors (TF) and their regulated target gene (TG) interactions, harbors Human Transcriptional Regulatory Interaction database (HTRIdb). LncRNAs are also included in the network interactions. In another network, we annotated differentially expressed genes as co-expressed, physically interacting and pathway genes using GeneMania for further analysis (Fig. S1). These two types of interaction data were imported into Cytoscape 3.5.1 (http://www.cytoscape.org/download.php). The network analyzer tool—using the directed network with TF as the source and gene as the target (directed TF to gene network)—was used to produce quantitative data for node degree, betweenness centrality, clustering coefficient among others.

## Result

**Figure 1:**
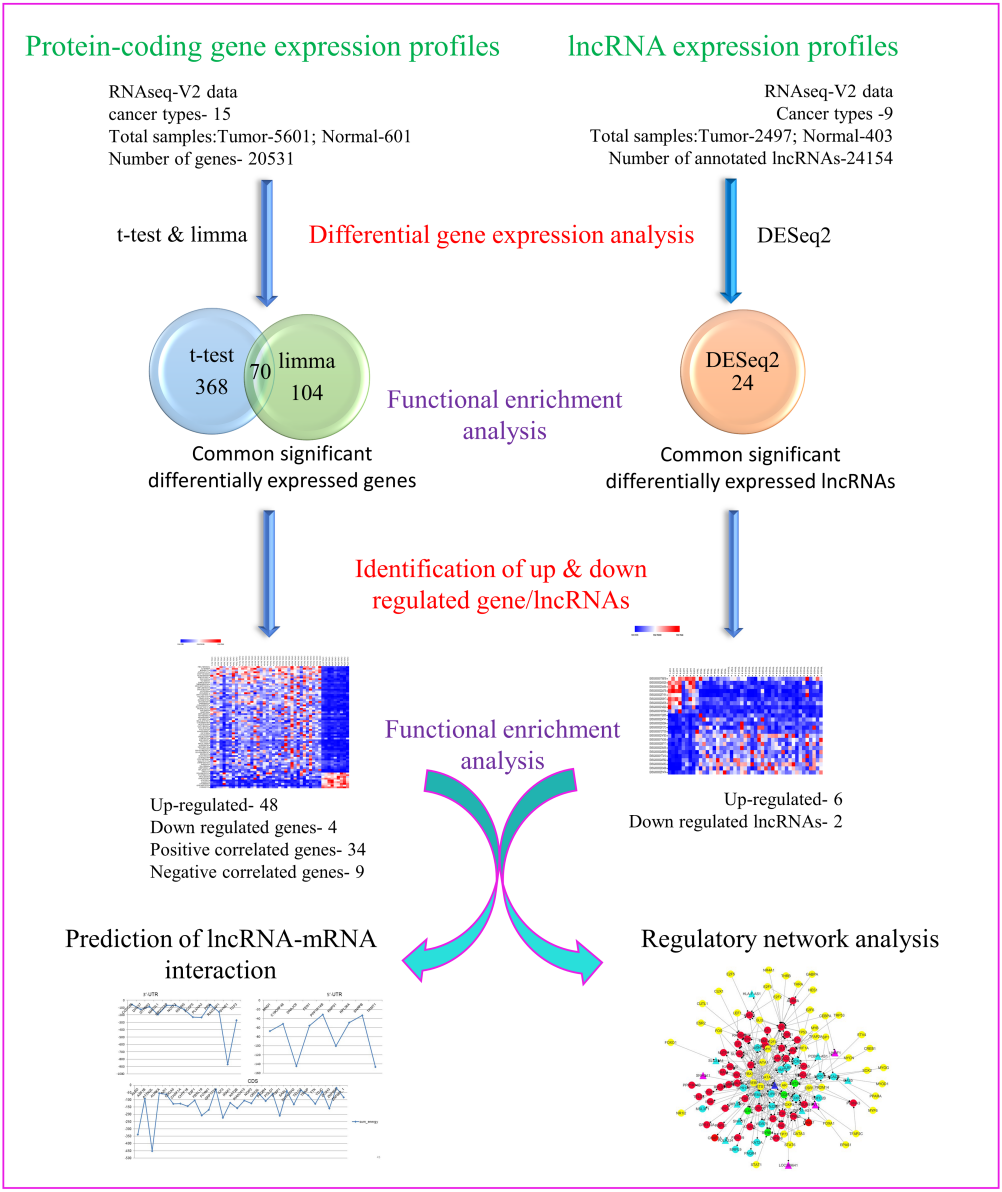
Flowchart depicting different steps used and narrowing down genes as seen through corresponding number of coding and non-coding genes.

Towards our studies on understanding interrelationship between coding and non-coding genes playing possible essential roles in pan-cancer development, we have used several well-established and validated tools to generate hypothesis and gain useful insights. A concise summary of various methods used has been put together as a flowchart in Fig. 1.

### Differential expression analysis of coding RNAs (mRNA)

**Figure 2:**
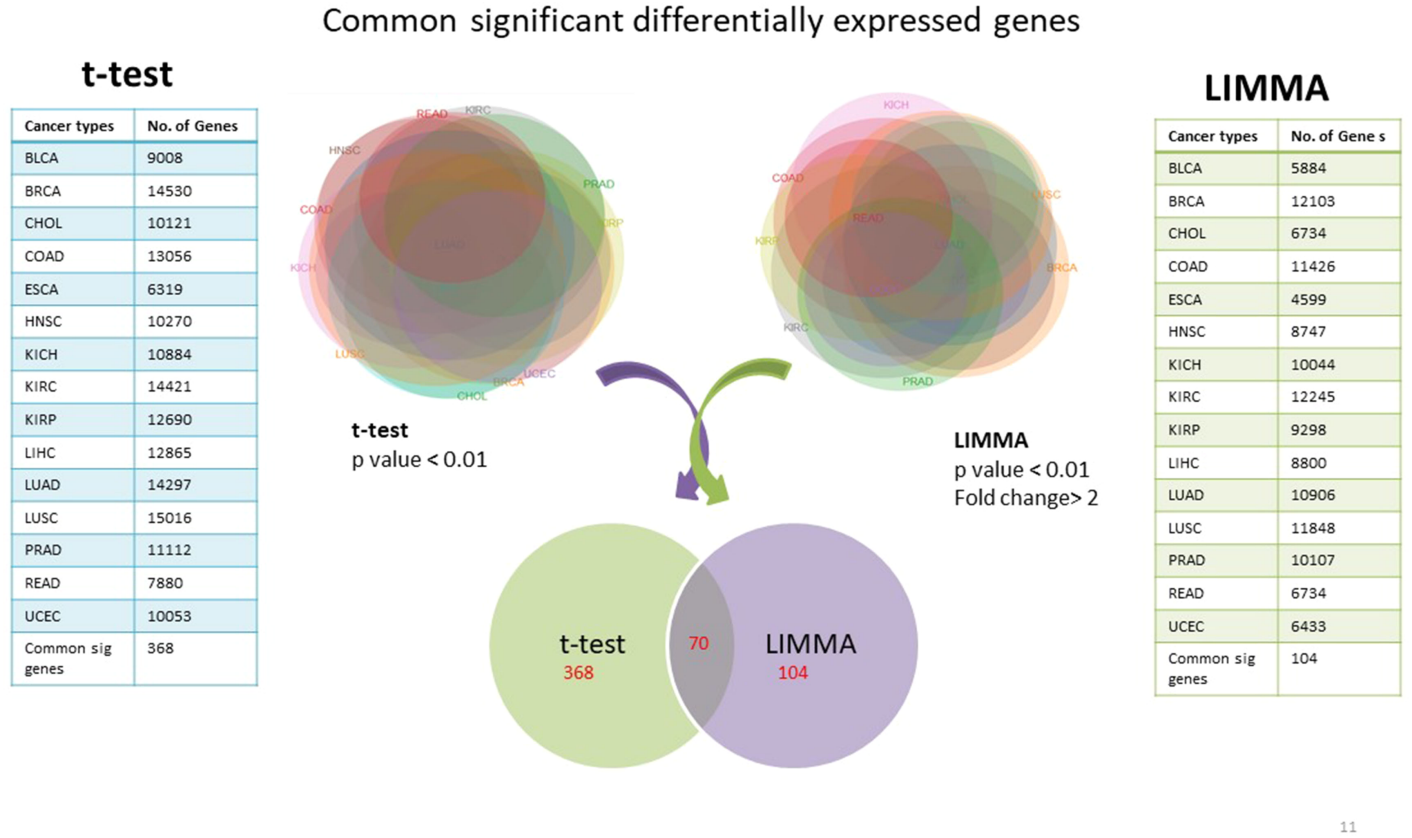
Common significantly differentially expressed protein-coding genes from *t*-test (at *p*-value <0.01) & LIMMA (at *p*-value <0.01, fold change >2.0).

Using TCGA RNAseqv2 data, we analyzed a total of 6,202 samples from 15 cancer types out of which 5,601 were tumors and 601 were corresponding normal samples. Total number of coding genes analyzed was 20,531. The number of samples within each tumor type is enlisted in Table 1. 368 significantly differentially expressed genes common to all cancer types were identified using unpaired *t*-test with critical *p*-value <0.01 and 104 such genes were selected using LIMMA (Linear Model for Microarray Analysis) with *p*-value <0.01 and fold change >2 (Fig. 2). In order to narrow down to more significant genes list, we pooled together genes from both *t*-test and LIMMA results and constructed a Venn diagram. 70 common significantly differentially expressed genes were enlisted (Table 2).

**Table 1:** Tumor types and number of samples in each tumor in our study taken from TCGA RNAseq v2 data. These cancer types are used in studies on protein-coding gene expression. Italicized cancer types are those used in non-protein coding lncRNA expression studies also.

S. NO	Cancer type	Tumor samples	Normal samples	Total
1	*Bladder Urothelial Carcinoma [BLCA]*	408	19	427
2	*Breast invasive carcinoma [BRCA]*	1,112	113	1,225
3	Cholangiocarcinoma [CHOL]	36	9	45
4	Colon adenocarcinoma [COAD]	286	41	327
5	Esophageal carcinoma [ESCA]	185	11	196
6	*Head and Neck squamous cell carcinoma [HNSC]*	522	44	566
7	Kidney Chromophobe [KICH]	66	25	91
8	*Kidney renal clear cell carcinoma [KIRC]*	534	72	606
9	*Kidney renal papillary cell carcinoma [KIRP]*	290	32	322
10	*Liver hepatocellular carcinoma [LIHC]*	374	50	424
11	*Lung adenocarcinoma [LUAD]*	517	59	576
12	*Lung squamous cell carcinoma [LUSC]*	502	51	553
13	*Prostate adenocarcinoma [PRAD]*	497	52	549
14	Rectum adenocarcinoma [READ]	95	10	105
15	Uterine Corpus Endometrial Carcinoma [UCEC]	177	13	190
		Total		6,202

**Table 2:** Common significantly differentially expressed genes in all 15 types of cancer.

Gene name	Protein name	Gene name	Protein name
*ACACB*	Acetyl-CoA Carboxylase Beta	*NOP2*	NOP2 Nucleolar Protein
*ALAD*	Aminolevulinate Dehydratase	*NR2C2AP*	Nuclear Receptor 2C2 Associated Protein
*ASF1B*	Anti-Silencing Function 1B Histone Chaperone	*NR3C2*	Nuclear Receptor Subfamily 3 Group C Member 2
*ATXN2L*	Ataxin 2 Like	*NSUN5*	NOP2/Sun RNA Methyltransferase Family Member 5
*AURKA*	Aurora Kinase A	*NUP85*	Nucleoporin 85
*AZI1(CEP13)*	5-Azacytidine-Induced Protein 1 (Centrosomal Protein 131)	*NUTF2*	Nuclear Transport Factor 2
*C19orf48*	Chromosome 19 Open Reading Frame 48	*OBFC2B*	Nucleic Acid Binding Protein 2
*C20orf20*	MRG/MORF4L Binding Protein	*ORC6L*	Origin Recognition Complex Subunit 6
*CCDC86*	Coiled-Coil Domain Containing 86	*PAQR4*	Progestin And AdipoQ Receptor Family Member 4
*CDCA5*	Cell Division Cycle Associated 5	*PCGF5*	Polycomb Group Ring Finger 5
*CHAF1A*	Chromatin Assembly Factor 1 Subunit A	*PLXNA3*	Plexin A3
*CHTF18*	Chromosome Transmission Fidelity Factor 18	*POLD1*	Polymerase (DNA) Delta 1, Catalytic Subunit
*DHX37*	DEAH-Box Helicase 37	*PPIA*	Peptidylprolyl Isomerase A
*DNAJC9*	DnaJ Heat Shock Protein Family (Hsp40) Member C9	*PPP1R14B*	Protein Phosphatase 1 Regulatory Inhibitor Subunit 14B
*E2F1*	E2F Transcription Factor 1	*PTBP1*	Polypyrimidine Tract Binding Protein 1
*EHMT2*	Euchromatic Histone-Lysine N-Methyltransferase 2	*RACGAP1*	Rac GTPase Activating Protein 1
*FBXL19*	F-Box And Leucine-Rich Repeat Protein 19	*RNPS1*	RNA Binding Protein With Serine Rich Domain 1
*FEN1*	Flap Structure-Specific Endonuclease 1	*RPL36A*	Ribosomal Protein L36a
*FOXM1*	Forkhead Box M1	*SAAL1*	Serum Amyloid A Like 1
*FOXN3*	Forkhead Box N3	*SLBP*	Stem-Loop Binding Protein
*GPR146*	G Protein-Coupled Receptor 146	*SNRPB*	Small Nuclear Ribonucleoprotein Polypeptides B And B1
*GPR172A*	Solute Carrier Family 52 Member 2	*SUV420H2*	Lysine Methyltransferase 5C
*GTPBP3*	GTP Binding Protein 3	*SYNE1*	SYNE1
*HN1L*	Hematological And Neurological Expressed 1-Like	*SYNJ2BP*	Synaptojanin 2 Binding Protein
*ILF3*	Interleukin Enhancer Binding Factor 3	*TCF3*	Transcription Factor 3
*IRAK1*	Interleukin 1 Receptor Associated Kinase 1	*TELO2*	Telomere Maintenance 2
*KAT2A*	Lysine Acetyltransferase 2A	*THOC4*	Aly/REF Export Factor
*KAT2B*	Lysine Acetyltransferase 2B	*TMEM206*	Transmembrane Protein 206
*KIAA0415*	Adaptor Related Protein Complex 5 Zeta 1 Subunit	*TRAIP*	TRAF Interacting Protein
*MAD2L1*	MAD2 Mitotic Arrest Deficient-Like 1	*TRMT1*	TRNA Methyltransferase 1
*MCM3*	Minichromosome Maintenance Complex Component 3	*TTK*	TTK Protein Kinase
*MND1*	Meiotic Nuclear Divisions 1	*WDR75*	WD Repeat Domain 75
*MRPL9*	Mitochondrial Ribosomal Protein L9	*YDJC*	Not Available
*MSH5*	MutS Homolog 5	*ZC3H3*	Zinc Finger CCCH-Type Containing 3
*MSL3L2*	Not Available	*ZNF668*	Zinc Finger Protein 668

#### Identification of up- and down- regulated genes

**Figure 3:**
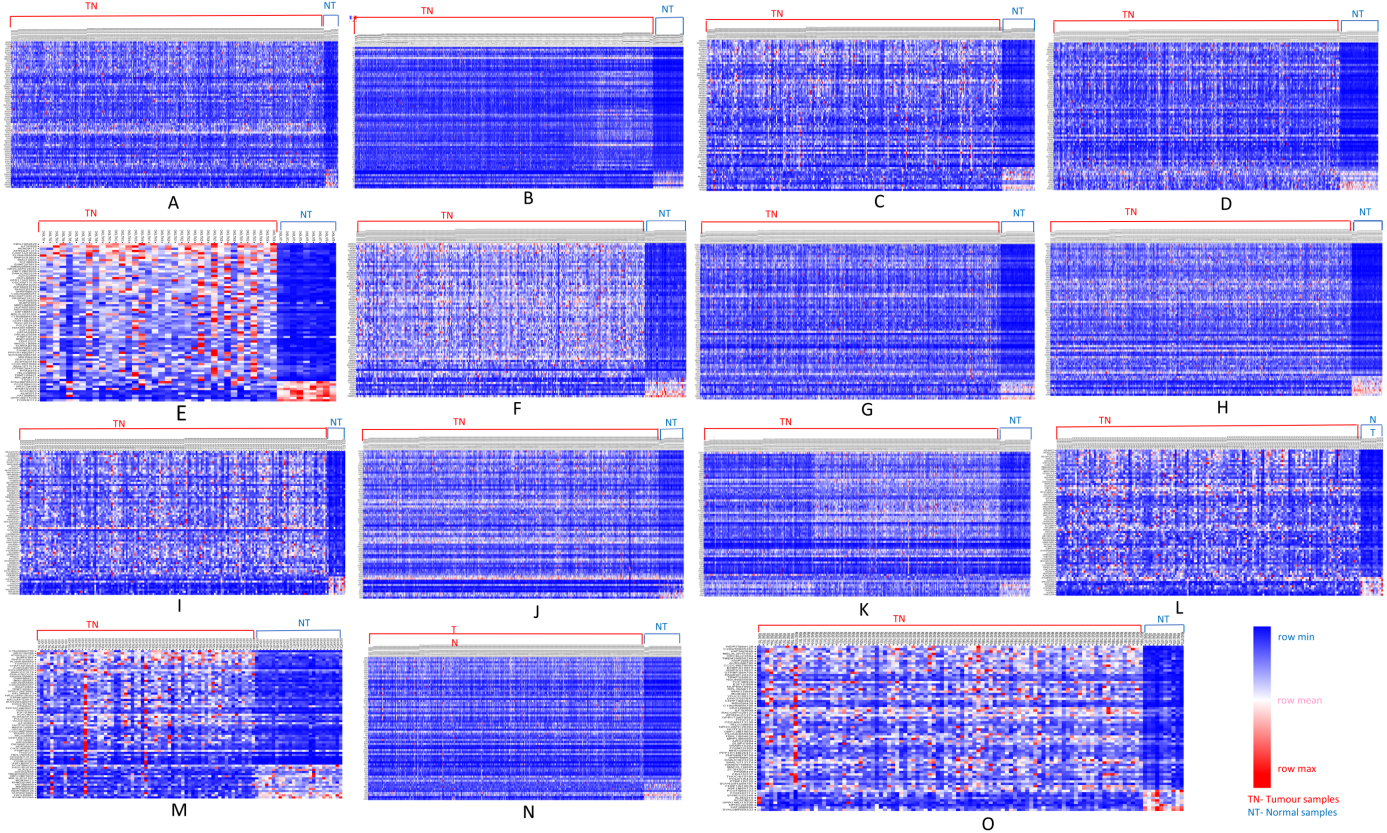
Heatmap showing up- and down-regulated protein-coding genes in 15 types of tumors. Rows refer to genes and columns are the samples. The red color bar indicates genes with higher expression levels and the blue color indicates genes with lower expression levels. N: Normal samples, T: Tumor samples. (A) BLCA, (B) BRCA, (C) KIRP, (D) LIHC, (E) CHOL, (F) COAD, (G) LUAD, (H) LUSC, (I) ESCA, (J) HNSC, (K) PRAD, (L) UCEC, (M) KICH, (N) KIRC, (O) READ.

We further analyzed our data to identify which of these genes are up-regulated and which ones are down-regulated in cancers as compared to normal tissues. For this purpose, comparative marker selection in GenePattern suite of programs was used. We observed that out of these 70 commonly dysregulated genes, 48 were up-regulated and 4 genes down-regulated in all 15 types of tumors while remaining 18 were significantly differentially expressed genes that differed in their expression pattern in one or two tumor types only and therefore, still taken as showing a uniform pattern of expression (Fig. 3). These have been tabulated in Table 3.

**Table 3:** List of up-and down-regulated genes common across 15 cancer types.

	Genes	
Up-regulated genes	*ASF1B, ATXN2L, AURKA, AZI1, C19orf48, C20orf20, CCDC86, CDCA5, CHAF1A, CHTF18, DHX37, DNAJC9, E2F1, FBXL19, FEN1, FOXM1, GPR172A, GTPBP3, ILF3, IRAK1, KIAA0415, MAD2L1, MND1, NOP2, NR2C2AP, NSUN5, NUTF2, ORC6L, PAQR4, PLXNA3, POLD1, PPIA, PPP1R14B, PTBP1, RACGAP1, RNPS1, RPL36A, SAAL1, SNRPB, SUV420H2, TCF3, TELO2, TRAIP, TRMT1, TTK, YDJC, ZC3H3,ZNF668*	These genes are commonly up-regulated in all 15 types of cancers.
Down-regulated genes	*ALAD, KAT2B, PCGF5, SYNE1.*	These genes are down-regulated in 15 types of cancers.
Common significantly differentially expressed genes up-regulated or down-regulated in 1 or 2 tumors	*ACACB, EHMT2, FOXN3, GPR146, HN1L, KAT2A, MCM3, MARPL9, MSH5, MSL3L2, NR3C2, NUP85, OBFC2B, SLBP, SYNJ2BP, THOC4, TMEM206, WDR75.*	

#### Functional annotation

We annotated the functioning of these 70 common significantly differentially expressed genes using GeneCodis3 web-server implementing Gene Ontology (GO) resources for biological processes. Except two genes, these genes (Table 4) were found to be involved in prominent biological processes such as gene expression, DNA-dependent regulation of transcription, transcription from RNA polymerase-II, DNA replication, mitotic cell cycle and DNA repair in decreasing order of the number of genes involved in each biological process. These are all major processes widely implicated in tumor development and progression.

**Table 4:** Functional annotation of common significantly differentially expressed protein-coding genes.

GO.id	No. of genes	Biological process	Hypergeometric *p*-value	Name of genes
GO:0010467	9	gene expression (BP)	1.18E–07	*RPL36A, NR2C2AP, PTBP1, KAT2B, SNRPB, RNPS1, SLBP, THOC4, NR3C2*
GO:0006355	10	regulation of transcription, DNA-dependent (BP)	0.00124323	*ZNF668, C20orf20, CHAF1A, ASF1B, PCGF5, FOXM1, TCF3, E2F1, SUV420H2, NR3C2*
GO:0000278	8	mitotic cell cycle (BP)	1.65E–07	*MAD2L1, AZI1, POLD1, FEN1, E2F1, AURKA, NUP85, MCM3*
GO:0006366	6	transcription from RNA polymerase II promoter (BP)	2.68E–05	*KAT2A, FOXM1, SNRPB, RNPS1, SLBP, THOC4*
GO:0006260	5	DNA replication (BP)	1.35E–05	*CHAF1A, POLD1, FEN1, MCM3, CHTF18*
GO:0006281	6	DNA repair (BP)	2.25E–05	*OBFC2B, KIAA0415, CHAF1A, POLD1, FOXM1, FEN1*

#### Correlation with protein expression levels

It is widely known that expression levels of mRNA and protein may not correlate at times due to the stability issues of mRNA and corresponding protein and further post-transcriptional modifications and regulatory processes. Further, if proteins are to be used as targets, it is important that their levels correlate with their mRNA levels too. In our attempt to understand further if mRNA and protein levels correlate in our list of genes, we used Human Protein Atlas (HPA) database. In HPA, protein expression levels are scored based on staining intensity score from immunohistochemistry experiments in cancer tissues. When mRNAs were found to be up-regulated or down-regulated in tumors and corresponding proteins had a score of high and low immunoreactivity, respectively, it was deciphered to be a positive correlation. A negative correlation was understood to be present when protein intensity score was high while mRNA is downregulated and *vice versa*. In some cases, we were unable to establish a clear correlation and categorized these genes as ‘uncorrelated or not able to correlate’. Thus, out of 70 genes, 34 genes were positively correlated, 9 genes negatively correlated, and remaining 16 genes were found to be uncorrelated (Table 5 and Fig. S2). Data was not available for 11 of these. Of these 34 positively correlated genes, using GeneCodis3, we found that processes/annotations over-represented for majority of genes were: gene expression, transcription from RNA polymerase-II promoter and DNA repair. Mitotic cell cycle and DNA replication were also among the biological processes involved (Table 6).

**Table 5:** Correlation of common significantly differentially expressed genes with protein levels (Human Protein Atlas database).

Correlation type	Genes	Description
Positively correlated genes	*ALAD, ATXN2L, CCDC86, CDCA5, CHAF1A, DHX37, DNAJC9, E2F1, FEN1, FOXM1, GTPBP3, ILF3, IRAK1, KAT2A, MRPL9, NR3C2, NUP85, ORC6L PCGF5, POLD1, PPIA, PPP1R14B, PTBP1, RACGAP1, SAAL1, SNRPB, SLBP, SYNE1, TELO2, THOC4, TRAIP, TTK, WDR75, ZNF668*	Similar expression levels of both mRNA and protein (protein expression levels are calculated based on “colour intensity score”).
Negatively correlated genes	*AURKA, AZI1, C19ORF48, MAD2L1, MND1, RNPS1, RPL36A, ZC3H3, NUTF2*	High mRNA expression values and negative color intensity score for proteins and vice versa.
Not able to establish clear correlation	*ACACB, EHMT2, FOXN3, GRP172A, HN1L, KAT2B, MCM3, NR2C2AP, NOP2, NSUN5, OBFC2B, SYNJ2BP, SUV420H2, TCF3, TMEM206, TRMT1*	These genes are having unique expression pattern at mRNA and protein level.
Not available	*ASF1B, C20ORF20, CHTF18, FBXL19, GPR146, KIAA0415, MSH5, MSL3L2, PAQR4, PLXNA3, YDJC, PLXNA3.*	These genes are not available in “Human Protein Atlas database.

**Table 6:** Functional annotation of positively correlated protein-coding genes.

GO.id	No. of genes	Biological process	Hypergeometric *p*-value	Name of genes
GO:0006355	6	regulation of transcription, DNA-dependent	0.00398166	*ZNF668, CHAF1A, PCGF5, FOXM1, E2F1, NR3C2*
GO:0010467	5	gene expression	4.25E–05	*PTBP1, SNRPB, SLBP, THOC4, NR3C2*
GO:0006366	5	transcription from RNA polymerase II promoter	8.83E–06	*KAT2A, FOXM1, SNRPB, SLBP, THOC4*
GO:0007165	4	signal transduction	0.0257568	*RACGAP1, IRAK1, TRAIP, NR3C2*
GO:0007049	4	cell cycle	0.000788097	*RACGAP1, CHAF1A, FOXM1, CDCA5*
GO:0006281	4	DNA repair	0.000159569	*CHAF1A, POLD1, FOXM1, FEN1*
GO:0000278	4	mitotic cell cycle	0.000204197	*POLD1, FEN1, E2F1, NUP85*
GO:0008283	3	cell proliferation	0.00334808	*KAT2A, E2F1, TRAIP*

Involvement of these genes/proteins in cancer development and progression and their mechanistic actions have been detailed in a separate Supplemental File.

The genes *MSL3L2* or *MSL3P1* and *YDJC* appear to be novel and their functions in cancer are unknown, to the best of our knowledge.

To further understand their regulation in cancers, we turned our focus on all of these 34 key essential genes. Apart from regular gene regulators, lncRNAs are among the emerging regulators involved in biological processes predominantly through epigenetic regulation and found to be powerful regulators in cancer as well. Further, epigenetic regulation is at the heart of almost all the biological processes over-represented in our gene set. Therefore, in order to gain further regulatory and functional insights, we proceeded for lncRNA studies.

### Differential expression analysis of long non-coding RNAs (lncRNAs)

**Figure 4:**
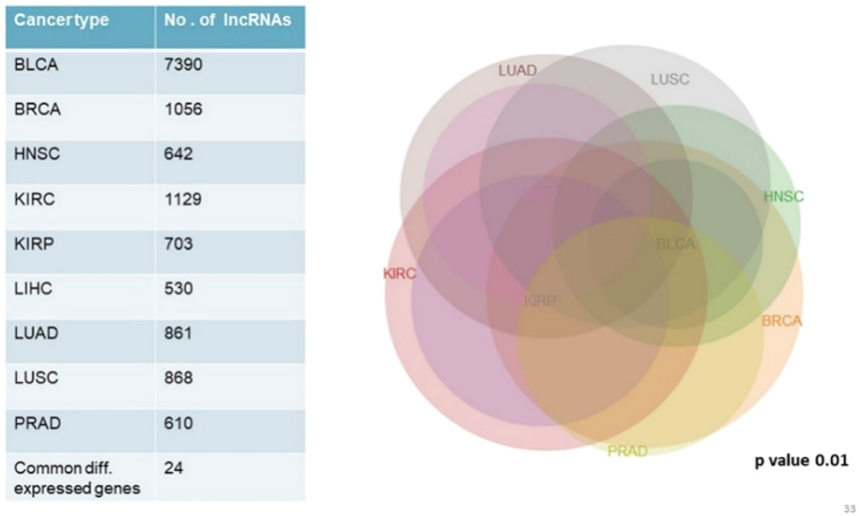
Common significantly differentially expressed noncoding genes (lncRNAs) in nine types of tumors.

We analyzed and compared lncRNA expression profiles from 2,497 total samples in 9 types of cancer with their corresponding normal samples numbering 403, using lncRNAtor database. Our objective was not to compare mRNA and lncRNA expression profiles together but to enlist a list of significantly differentially expressed genes common to all cancer types studied. Among significantly aberrantly expressed lncRNA genes in each cancer type identified in lncRNAtor, 24 common lncRNA genes were enlisted (Fig. 4; Table 7).

**Table 7:** Common significantly differentially expressed lncRNAs in nine types of cancers.

	lncRNA ID	*lncRNA NAME*
Up-regulated lncRNAs in tumor	ENSG00000249859 ENSG00000034063 ENSG00000236455 ENSG00000174365 ENSG00000246582 ENSG00000261373	*PVT1* *UHRF1* *AC005154.5* *SNHG11* *RP11-1149O23.3* *RP11-368I7.2*
Down-regulated lncRNAs in tumor	ENSG00000234456 ENSG00000186594	*MAGI2-AS3* *MIR22HG*
Tumor-specific differentially expressed lncRNAs	ENSG00000214922 ENSG00000256594 ENSG00000137700 ENSG00000179818 ENSG00000214783 ENSG00000245025 ENSG00000251474 ENSG00000172965 ENSG00000234636 ENSG00000260917 ENSG00000255717 ENSG00000224078 ENSG00000257151 ENSG00000177410 ENSG00000234741 ENSG00000203499	*HLA-F-AS1* *RP11-705C15.2* *SLC37A4* *PCBP1-AS1* *POLR2J4* *RP11-875O11.1* *RPL32P3* *AC068491.1* *MED14-AS1* *RP11-57H14.4* *SNHG1,* *SNHG14* *RP11-701H24.2* *ZNFX1-AS1* *GAS5* *RP11-429J17.6*

#### Identification of up- and down-regulated lncRNAs

**Figure 5:**
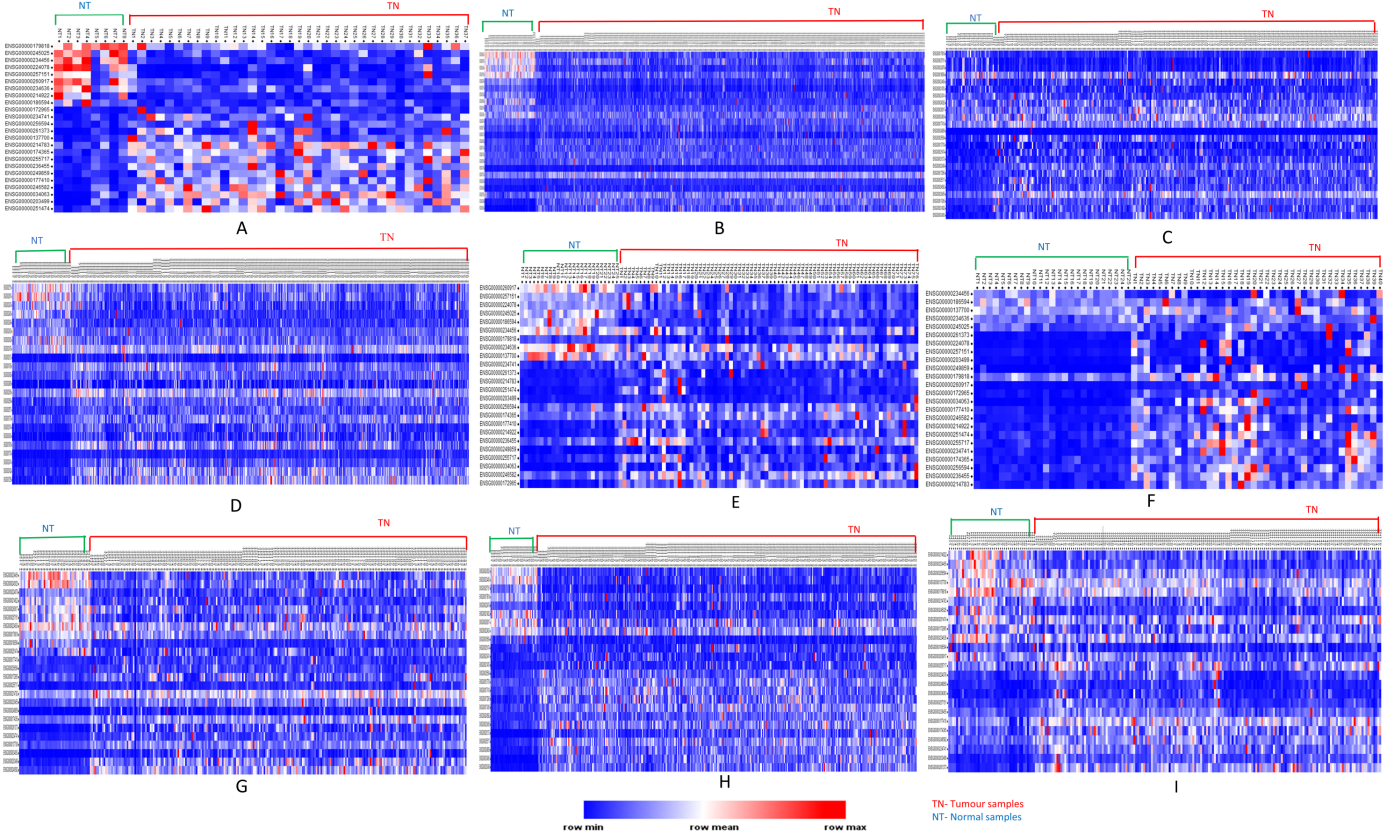
Heatmap showing common up- and down-regulated lncRNA genes in tumors. Rows refer to lncRNA and columns are the samples. The red color bar indicates lncRNAs with higher expression levels and the blue color indicates lncRNAs with lower expression levels. N: Normal samples; T: Tumor samples. (A) BLCA, (B) BRCA, (C) HNSC, (D) KIRC, (E) KIRP, (F) LIHC, (G) LUAD, (H) LUSC, (I) PRAD.

Analyses of up- and down-regulated status of these 24 common significantly differentially expressed lncRNAs, showed that lncRNAs *PVT1, UHRF1, AC005154.5, SNHG11, RP11-1149O23.3,* and *RP11-368I7.2* were up-regulated in tumors; *MAGI2-AS3, MIR22HG* down-regulated in tumors and the remaining were tumor-specific differentially expressed lncRNAs (Fig. 5).

#### Functional analyses of dysregulated lncRNAs

In light of the above studies and our own observations, we proceeded to study the function of lncRNA molecules in more detail. Function of a few lncRNAs such as *Xist* and *NEAT1*, both involved in cancer progression, have been experimentally validated (Patil et al., 2016; Qian et al., 2017). While *Xist* is involved in X-chromosome inactivation (Xiong et al., 2017; Zhang et al., 2017; Song et al., 2016a), *NEAT1* plays a regulatory role in initiation and progression of cancers (Fang et al., 2017; Yang et al., 2017a; Li et al., 2016). *PVT1* lncRNA functions as an oncogene by acting as a sponge for miRNAs (Li et al., 2018; Liu et al., 2016b; Li, Meng & Yang, 2017) as well as several other mechanisms as has been detailed above. Function of many lncRNAs remains to be discovered. One of the several approaches used to know the likely functions of lncRNAs is “guilt-by-association” with co-expressed protein-coding mRNAs, as these protein-coding genes are likely to be co-regulated by lncRNAs synergistically (Sun et al., 2015). In this manner, role of lncRNAs in specific biological processes and molecular function can be predicted.

**Figure 6:**
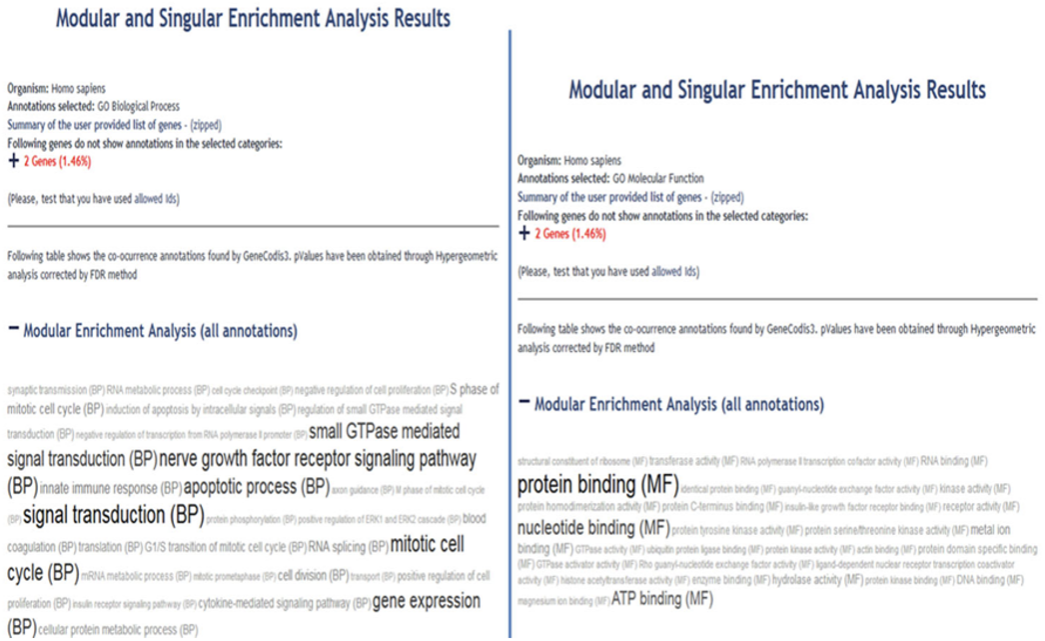
Functional enrichment of *PVT1* lncRNA using co-expressed protein coding genes from RNAseq data of bladder urothelial carcinoma (representative figure) using GeneCodis. Hypergeometric *p*-value of < or = 0.05 (default) was used.

We wanted to find out the function of lncRNAs in our narrowed down list. As no *de-novo* function prediction tool for lncRNAs is available as yet, we used lncRNAtor which predicts lncRNA function from co-expression of protein-coding mRNAs. We characterized each lncRNA for co-expressed mRNAs from lncRNAtor for each of the 9 cancer types. These co-expressed mRNAs were analyzed for their enrichment in biologicals processes and molecular function using GeneCodis3. Most prominent biological processes are common in which these lncRNAs may be involved through “guilt-by-association” are: signal transduction, gene expression, mitotic cell cycle, nerve growth factor receptor signaling pathway, apoptotic process and blood coagulation, in that order. DNA repair and DNA replication processes were also predicted. The predicted molecular functions are in protein binding, nucleotide-binding and ATP binding (Fig. 6 and Fig. S3 ). It is highly interesting to note from our observations that the major biological processes predicted for lncRNAs also correspond to the biological processes predicted for our significantly differentially expressed protein-coding gene list. This shows that these lncRNAs and protein-coding genes from our list may be interacting or regulating each other in one way or another.

### Prediction of interactions between dysregulated mRNAs and lncRNAs

**Figure 7:**
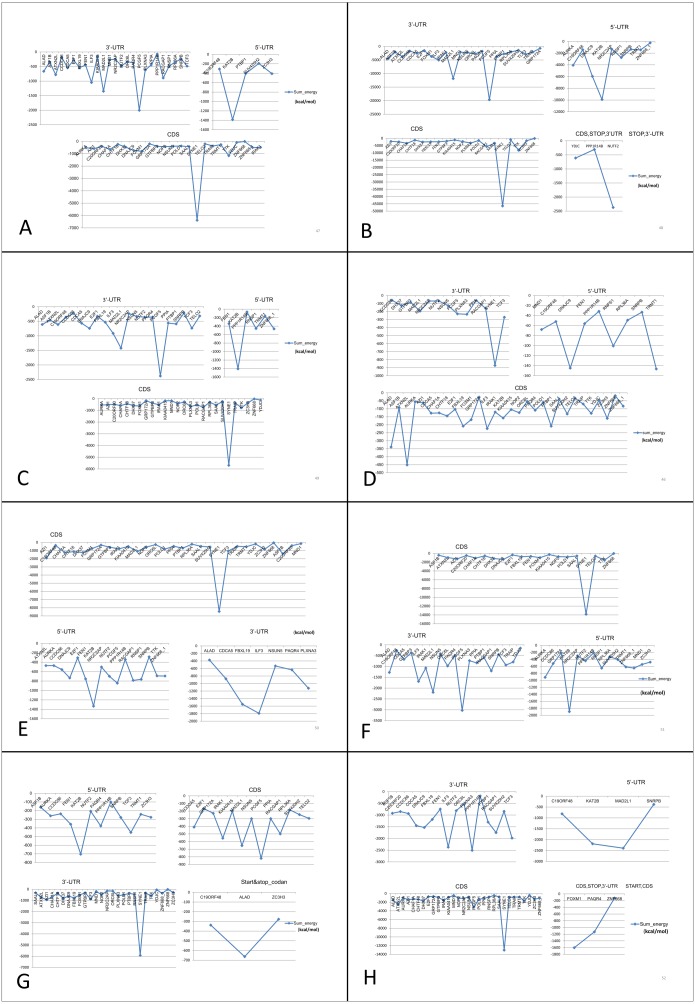
Prediction of lncRNA-mRNA interaction and their binding sites. Their corresponding binding energies (sumenergy) show the intensity of interactions with higher negative binding energy meaning tighter association leading to more favorable or stable interaction. (A) *AC005154.5*, (B) *MAGI2-AS3*, (C) *MIR22HG*, (D) *PVT1*, (E) *RP11-368I7.2*, (F) *RP11-1149O23.3*, (G) *SNHG11*, (H) *UHRF1*.

We predicted possible interactions between these eight common (both up- and down-regulated) lncRNAs and mRNAs using lncRNA-mRNA interaction database. Binding energy (sumenergy) values of each interaction were plotted (Fig. 7) and significant interactions based on lower binding energy values were enlisted (Table 8). From the patterns observed from our plots, we identified that *SNHG11, AC005154.5, MAGI2-AS3, MIR22HG, RP11-368I7.2, RP11-1149O23.3,* and *UHRF1* lncRNAs interacted with *KAT2B* mRNA at 5′-UTR with higher SUMENERGY values except *PVT1*. Further, all lncRNAs except *SNHG11* and *PVT1* interacted with *SYNE1* at CDS (Coding region). Both *SNHG11* and *PVT1* were predicted to interact with *SYNE1* at 3′UTR. *PVT1* had highest interactions with *DNAJC9* at 5′-UTR, and with *ATXN2L, ALAD, IRAK1* and *FOXM1* at CDS sites.

**Table 8:** Prediction of lncRNA-mRNA interactions and binding sites.

lncRNA	Protein-coding gene	Binding site	lncRNA	Protein-coding gene	Binding site
*AC005154.5*	*KAT2B* *SYNE1* *PCGF5* *MAD2L1* *ILF3*	5′UTR CDS 3′UTR 3′-UTR 3′-UTR	*RP11-368I7.2*	*KAT2B* *DNAJC9* *PCGF5* *SYNE1* *ILF3* *PLXNA3*	5′-UTR 5′-UTR 5′UTR CDS 3′-UTR 3′-UTR
*MAGI2-AS3*	*KAT2B* *SYNE1* *PCGF5* *MAD2L1*	5′-UTR CDS 3′-UTR 3′-UTR	*UHRF1*	*MAD2L1* *KAT2B* *SYNE1* *ILF3* *PLXNA3* *RACGAP1*	5′-UTR 5′-UTR CDS 3′-UTR 3′-UTR 3′-UTR
*MIR22HG*	*KAT2B* *SYNE1* *PCGF5* *MAD2L1*	5′-UTR CDS 3′-UTR 3′-UTR	*SNHG11*	*KAT2B* *TCF3* *PCGF5* *SYNE1* *ALAD*	5′-UTR 5′-UTR CDS 3′-UTR START & STOP
*PVT1*	*DNAJC9* *TRMT1* *RNPS1* *ATXN2L* *ALAD* *SYNE1*	5′-UTR 5′-UTR 5′-UTR CDS CDS 3′UTR	*RP11-1149O23.3*	*KAT2B* *AURKA* *RNPS1* *SYNE1* *PCGF5* *NSUN5* *IRAK1* *ALAD*	5′-UTR 5′-UTR 5′-UTR CDS 3′-UTR 3-UTR 3′UTR 3′-UTR

*MAGI2-AS3* and *MIR22HG* down-regulated lncRNAs both interact with same genes *KAT2B, SYNE1, PCGF5* and *MAD2L1* at same site. *PVT1* interacts with *ATXN2L* at CDS, *ATXN2L* is a positively correlated gene interacting with *PVT1* only. *RP11-1149O23.3* interacts with *AURKA* at 5′-UTR, *IRAK1* at 3′-UTR which are also positively correlated genes and *NSUN5* at 3′-UTR, these genes interact specifically with *RP11-1149O23.3* lncRNA. *SNHG11* and *UHRF1* have unique interactions with positively correlated genes *TCF3* and *RACGAP1* at 5′-UTR and at 3′-UTR, respectively.

### Network analysis of significantly differentially expressed mRNAs and lncRNAs

We constructed and analyzed context-specific regulatory network comprising of all the mRNAs/proteins and lncRNAs from our list of significantly differentially expressed molecules (master network). These networks constitute interaction signatures of transcription factors (TFs) and their regulated target genes. A total of 130 nodes (genes/proteins) and 311 edges (the interactions between these nodes) were taken from ORTI database to produce this master regulatory network. The network was analyzed on the basis of several parameters such as node degree, the degree to which one node is connected to other nodes in the network; betweenness centrality, which characterizes nodes that have many shortest paths going through them and clustering coefficient which refers to the extent to which nodes cluster together as a tight unit (Mishra, 2014; Albert & Barabasi, 2002). In a directed TF-gene network, node out-degree refers to number of genes regulated by a TF, while node in-degree refers to number of TFs that target/regulate a gene.

**Figure 8:**
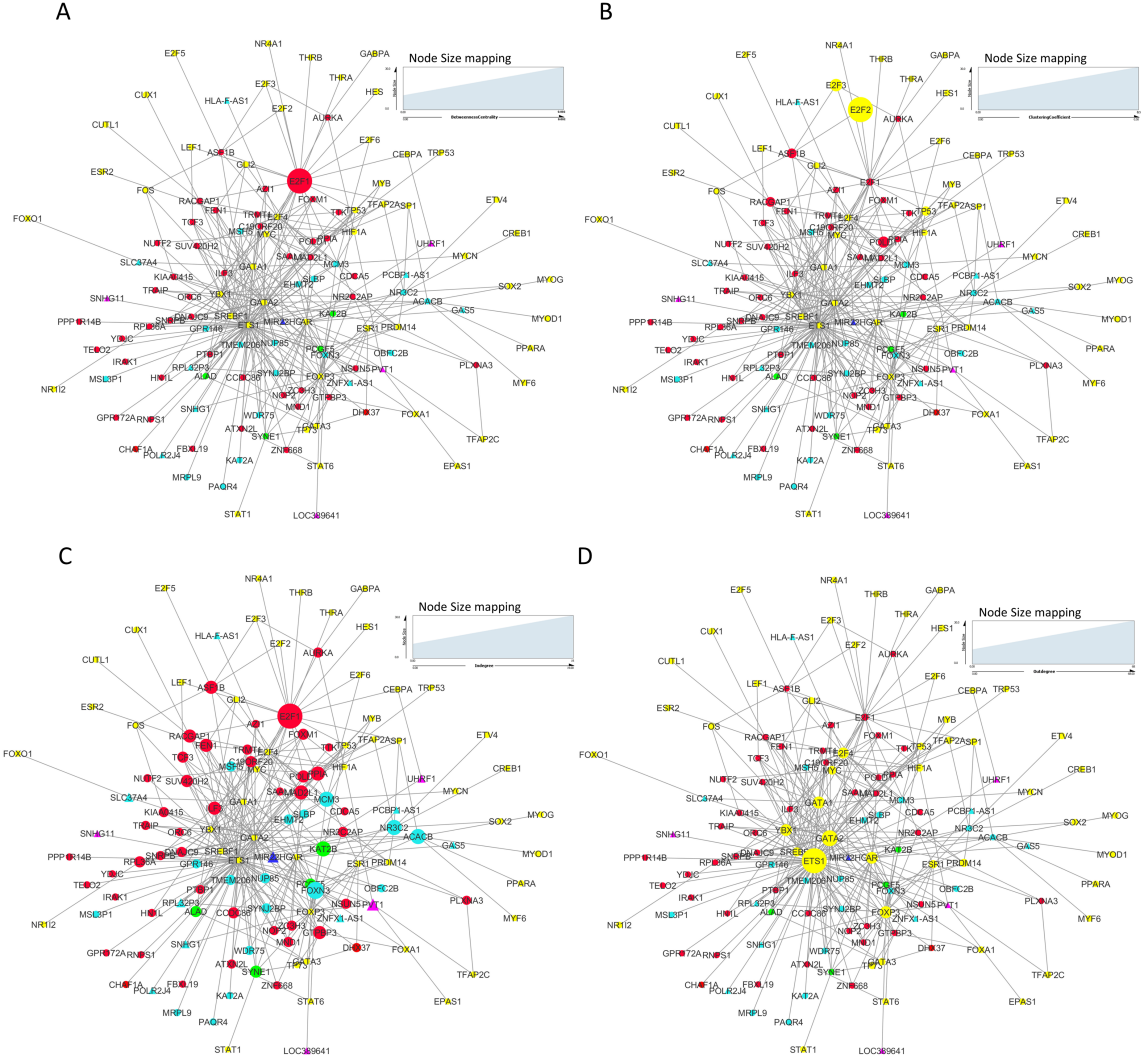
Master regulatory network parameters. (A) Betweenness centrality: *E2F1* showing highest betweenness centrality; (B) Clustering coefficient: *E2F2, E2F3, ASF1B, POLD1* and *RACGAP1* genes are clustered together; (C) Indegree: Number of inward directed nodes, *E2F1* has highest number of in-degree nodes; (D) Outdegree: Number of outward directed nodes, *ETS1* has highest out degree node. The node size increases with values of the respective topological parameter.

**Figure 9:**
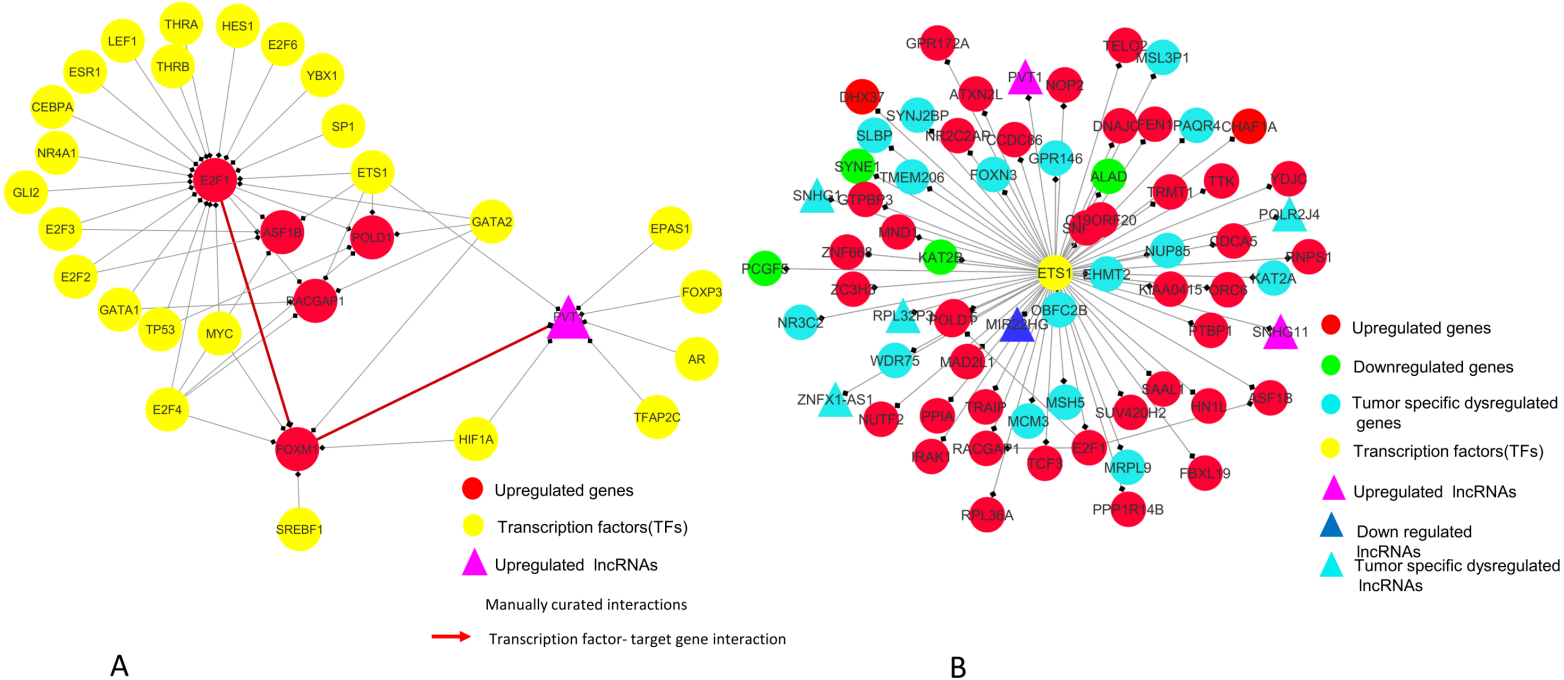
*E2F1* and *ETS1* subnetworks generated from master regulatory network. (A) *E2F1* subnetwork (generated from master regulatory network). *E2F1* is seen directly connected to tumor-specific dysregulated transcription factors and common significantly differentially expressed genes *POLD1, RACGAP1* and *ASF1B*. The red color edges are manually curated TF-TG interactions from the literature (Millour et al., 2011; De Olano et al., 2012). (B) ETS1 subnetwork (generated from master regulatory network). *ETS1* is a tumor-specific TF in at least one cancer type, directly connected with most of the common significantly differentially expressed coding and non-coding genes in all types of cancers studied.

In our master network, analyses of betweenness centrality, clustering coefficient and node degree parameters (Table 9) showed that the node with highest betweenness centrality was *E2F1* (Fig. 8A), the one with highest clustering coefficient was *E2F2* (Fig. 8B), highest degree node was *E2F1* for in-degree (Fig. 8C) and *ETS1* for out-degree (Fig. 8D). Nodes *E2F3, POLD1, RACGAP1* and *ASF1B* were also the nodes with higher clustering coefficient, some of which are again in our significantly differentially expressed and positively correlated with proteins list. From our network, we observed that *E2F1* regulates *POLD1, ASF1B, FOXM1* and *RACGAP1* which are up-regulated in all 15 cancer types (Subnetwork, Fig. 9A). *ETS1* regulates *E2F1* apart from *POLD1, ASF1B and RACGAP1* (Subnetwork, Fig. 9B). The direct interactions of these two essential genes with other genes shows that these directly interacting genes may also be playing a role in cancer development and progression by virtue of their association, and provide further insights.

**Table 9:** Regulatory network analysis of dysregulated protein coding and noncoding genes.

Genes	Betweenness centrality	Clustering coefficient	Indegree	Outdegree
*E2F1*	0.00272529	0.02597403	19	3
*E2F2*	0	0.5	0	2
*E2F3*	0	0.16666667	0	3
*POLD1*	0	0.13333333	6	0
*RACGAP1*	0	0.0952381	7	0
*ASF1B*	0	0.0952381	7	0
*FOXN3*	0	0	11	0
*KAT2B*	0	0	9	0
*NR3C2*	0	0	9	0
*MAD2L1*	0	0	8	0
*PVT1*	0	0	6	0
*FOXM1*	0	0	5	0
*ETS1*	0	6.58E–04	0	68
*GATA2*	0	0.00168067	0	35
*HIF1A*	0	0	0	5

On the basis of quantitative data, *E2F1* can be a hub bottleneck gene while *ETS1* can be a hub gene. Among lncRNAs, *PVT1* was found to be the node with highest degree thereby acting as a hub molecule. The other lncRNA with high node degree was *MIR22HG*. So, for analyzing subnetworks, we selected *E2F1* and *ETS1* among coding genes and *PVT1* among all lncRNAs genes for further deeper understanding based on the following criteria:

a. *E2F1* is a gene positively correlated with protein expression levels and is up-regulated in all tumor types studied. It also has highest node in-degree and highest betweenness centrality in the network.
b. *ETS1* is directly connected to *E2F1*. It is also the node with highest out-degree directly connected to *PVT1, SNHG11 and MIR22HG.* Though it is not in our list of common genes (it was connected by ORTI database while generating networks using our genes list), by dint of its direct connections to all other key molecules from our data and by being a proto-oncogene, it is also studied further to discern patterns in regulation.
c. *PVT1* lncRNA gene is up-regulated in all tumor types studied.
d. It is predicted to interact with *SYNE1* at 3′-UTR, with *DNAJC9* and *RNPS1* at 5′-UTR and *FOXM1, IRAK1, ATXN2L* and *ALAD* at CDS sites.
e. * PVT1* function is known, i.e., it is an oncogene. Its localization is both cytoplasmic and nuclear in SiHa (cervical cancer cell line) (Iden et al., 2016) while in 85% of SK-BR-3 breast cancer cells, a nuclear co-localization of MYC and *PVT1* was observed (Tseng et al., 2014).

While function of *SNHG11* is unknown, we hypothesize that primarily by its expression, interaction and network patterns similar to *PVT1*, its function can be predicted by extrapolation. Further, the only other coding gene interacting with it in the network is *ETS1*, the proto-oncogene as mentioned above, so its importance cannot be overlooked.

*ETS1* and *GATA2* transcription factors regulate most of the common significant differentially expressed genes/lncRNAs as seen from the node degree (especially out-degree) regulatory network (Fig. 8D). However, these two genes are not in our list of significantly differentially expressed gene across all tumor types, and were added by ORTI database during regulatory network construction. ETS1 showed negative immunoreactivity in all types of cancers while GATA2 showed moderate to strong positive immunoreactivity in all tumor types.

## Discussion

From our studies, we identified common coding and non-coding (lncRNAs) genes from various cancer types. Correlation pattern of mRNA with corresponding protein expression levels was also observed. Most of these genes were found to be involved in cancer progression-associated pathways such as gene expression, cell cycle regulation and nerve growth factor signaling. We hypothesized that the differential correlation between mRNA and protein expression levels may be due to the critical role of lncRNAs in regulating mRNA stability, degradation and role in protein synthesis.

Analysis of lncRNA-mRNA interactions of our dysregulated coding and non-coding genes list showed that in particular, *PVT1* lncRNA interacting genes were among common coding genes. *PVT1* is a widely studied lncRNA. *SYNE1 (Nesprin 1),* one of the *PVT1* interacting genes, is a nuclear envelope protein, residing in outer nuclear membrane, and has been shown to play a role in cancers, the mode being speculated to be through DNA damage response pathway (Sur, Neumann & Noegel, 2014). Interactions of all of the lncRNAs from our dataset with *SYNE1* mRNA shows its major prominent role. We hypothesize that since *SYNE1* is found to be a part of complex linking nucleoskeleton and cytoskeleton to maintain spatial organization, its mRNA interactions with lncRNAs may be somehow involved in regulation/dysregulation of cancer cell migration and metastasis through possible nuclear membrane rupture and repairing processes (Denais et al., 2016). The nuclear membrane rupture may also drive genomic instability in cancers (Lim, Quinton & Ganem, 2016). Even altered nuclear shape and size may be a cancer driver. Further studies with SYNE1 protein expression and lncRNA interactions will throw more light into its role in cancer development and progression in all cancer types.

*KAT2B* interactions with most of the lncRNAs from our list shows that these lncRNAs may play a major role in epigenetic regulation in cancers, since KAT2B protein is involved in lysine acetyltransferase activity in case of histone molecules. However, KAT2B is not in our list of molecules with positive correlation between mRNA and protein levels. From our analysis, *RNPS1* and *AURKA* mRNA expression level is negatively correlated with protein expression level. Because *PVT1* is predicted to interact with 5′-UTR region of *RNPS1* mRNA, and is found localized in both nucleus and cytoplasm of cervical cancer cells (Iden et al., 2016), this may be one possible mechanism of regulating *RNPS1* mRNA translation in cytoplasm. Similar case may hold for *RP11-1149O23.3* lncRNA in AURKA translation regulation. In case of other coding genes which are positively correlated with protein expression levels, the lncRNA interaction may play a role in mRNA stability.

**Figure 10:**
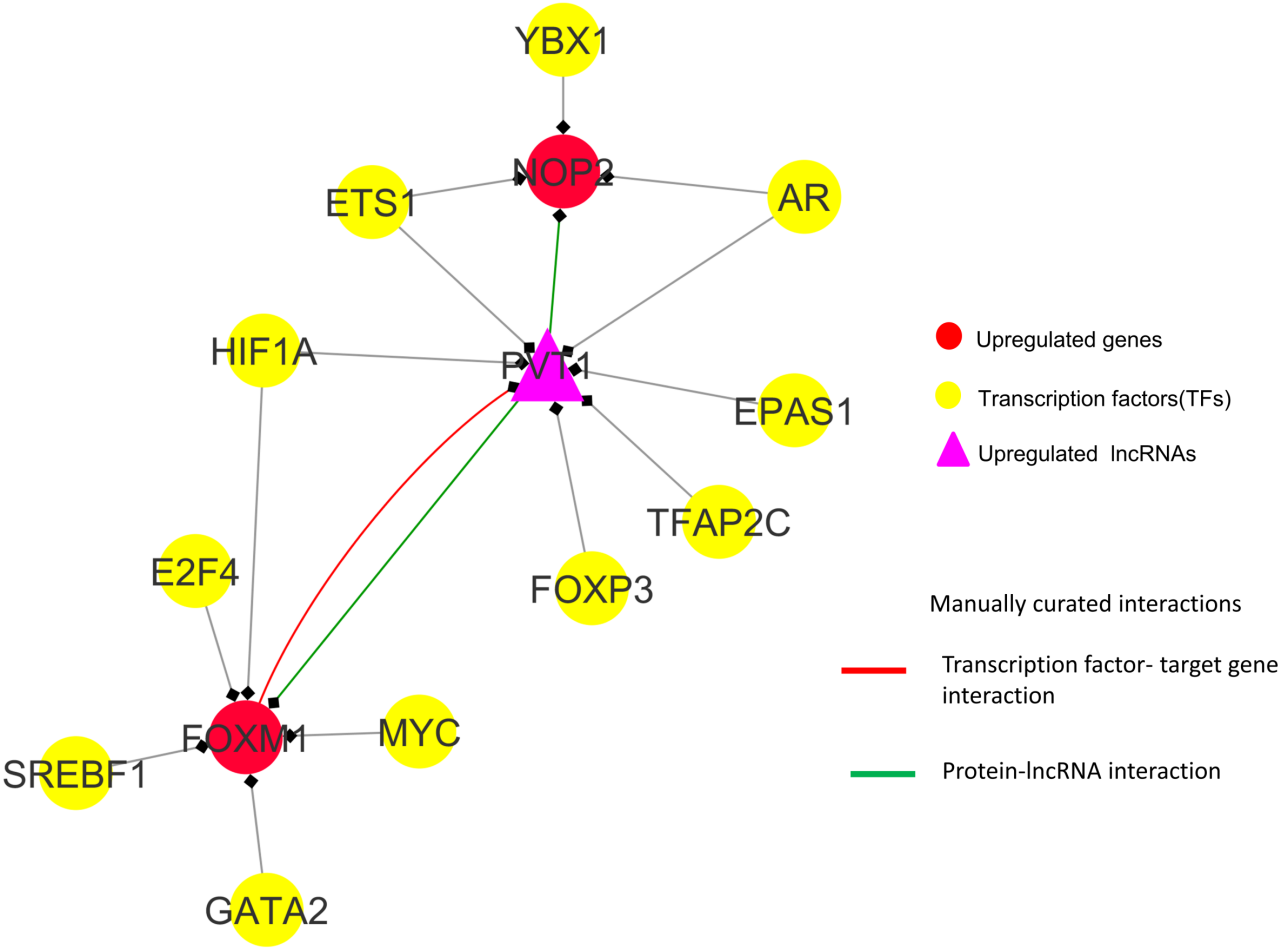
*FOXM1*, NOP2 and *PVT1* subnetwork generated from master regulatory network. TF-TG interaction between *FOXM1* and *PVT1 (red colored edge)*, lncRNA-protein interactions between *FOXM1*, NOP2 and *PVT1* (*green colored edges*) were taken from the literature and manually added as extra information (Xu et al. (2017) and Wang et al. (2014), respectively).

Observations from the connectivity patterns in the network show that E2F1 may be indirectly regulated by *PVT1*, and possibly also *SNHG11*, with ETS1 being a mediator between the two coding and non-coding genes. Further, *PVT1* transcription is regulated by FOXM1, another significantly differentially expressed coding gene from our results (Fig. 9A). *PVT1* also binds and stabilizes FOXM1 and NOP2 protein (Millour et al., 2011; Wang et al., 2014) (Fig. 10). This experimental conclusion from literature is validated by our prediction of lncRNA-mRNA interactions (Fig. 7).

**Figure 11:**
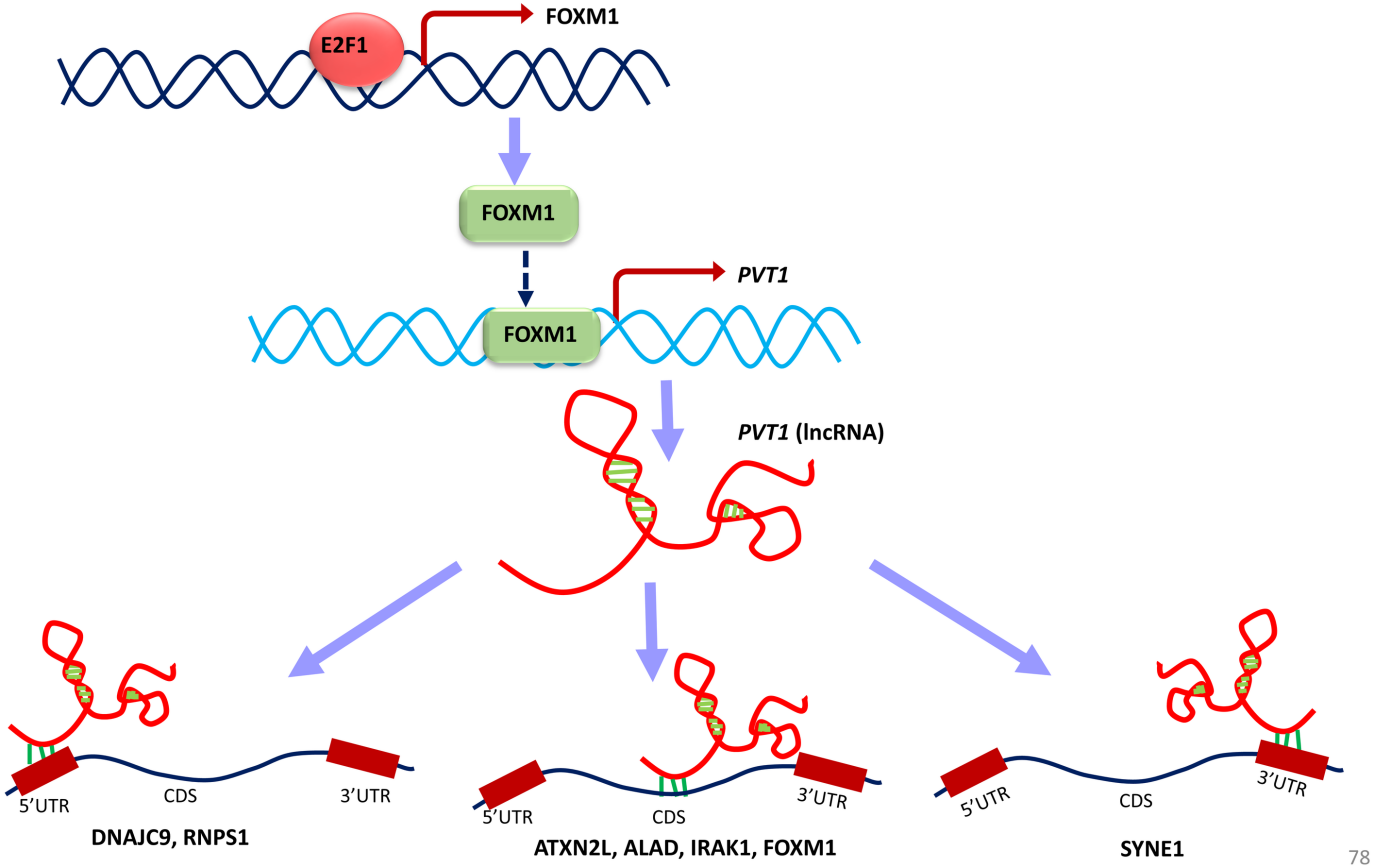
A unified model of pan-cancer regulation of common coding genes’ (mRNA) by non-coding genes (lncRNAs). *E2F1, FOXM1* and *PVT1* genes are identified as common significantly differentially expressed coding and non-coding genes from our analysis. E2F1 up-regulates FOXM1 expression and *FOXM1* up-regulates *PVT1* lncRNA expression. *PVT1* may play a role in regulation of mRNA splicing, stability and degradation of target genes to which it is predicted to bind such as *DNAJC9, RNPS1, ATXN2L, ALAD, IRAK1, FOXM1* and *SYNE1.*

Converging our varied set of results into one unified summary, we put forward a working model of the coding and non-coding genes’ regulation in all cancer types (Fig. 11). In the model, E2F1 regulates the FOXM1 transcription (Millour et al., 2011; De Olano et al., 2012), and when FOXM1 protein is translated, it regulates *PVT1* transcription. This E2F1-FOXM1-*PVT1* regulatory path was identified from our network studies. *PVT1* promotes cell progression, growth, invasion, and acquisition of stem cell-like properties by stabilizing *FOXM1* and NOP2 proteins in breast cancer and hepatocellular carcinoma, respectively (Millour et al., 2011; Wang et al., 2014). Our studies show that this may be the case in multiple cancer types as well. *PVT1* after being transcribed further binds to 5′-UTR, CDS and 3′-UTR of the genes in our results, specifically, *DNAJC9* (at 5′-UTR), *ATXN2L, ALAD, FOXM1, IRAK1* at CDS and *SYNE1* at 3′-UTR and may be regulating these genes through processes such as mRNA splicing, stability and degradation. *ETS1*, though not in our list of significantly differentially expressed gene across all tumor types, regulates at multiple levels: at the level of E2F1, FOXM1 and *PVT1*, but due to its negative immunoreactivity in all tumor types, it may be doing so at gene/mRNA level. Or it may be even working with non-coding genes to establish the regulatory orders. It remains to be seen, whether it may be involved in regulation in multiple cancer types. Further investigations will be required to understand if multiple lncRNAs can regulate the same gene or a set of genes by binding to the same or a different location. From a look at Table 8, from the given binding patterns, we surmise that multiple lncRNAs may regulate a single gene by binding to the same location at different stages in regulatory process. Several key genes may be missed because of limitations such as the *p*-value cutoff used and need for other cancer types to be included in case of lncRNA genes. The hypotheses generated here require further validation by qPCR and other molecular biology techniques.

## Conclusions

In this study, we have analyzed pan-cancer gene expression using RNASeq data and the mechanistic role and interplay of both coding and long non-coding RNA genes in pan-cancer gene regulation. Analyzing their cross-interacting patterns, among a total of about 20,531 protein-coding and as many non-coding RNA genes, we have zeroed in on the major players: E2F1, FOXM1 (transcription factors) and *PVT1* (lncRNA). The regulatory path defined by these key genes is a pan-cancer recurrent pattern. Our studies show that E2F1 transcription factor may be indirectly regulated by *PVT1* lncRNA. Transcription factor ETS1 may be playing a powerful mediator role between these two coding and non-coding genes. Several key insights gained into the interplay of these emerging novel class of gene regulators may help us understand pan-cancer gene regulation and provide novel cancer drug targets.

##  Supplemental Information

10.7717/peerj.6388/supp-1 Figure S1 GeneMania networks for differentially expressed genes as co-expressed, physically interacting and pathway genes First row shows co-expressed gene networks, second row as pathway gene networks and third row as physically intercating gene networks from our list of significantly differentially expressed genes.

10.7717/peerj.6388/supp-2 Figure S2 Protein expression levels as intensity scores taken from Human Protein Atlas For each protein, protein expression levels are scored based on staining intensity score from immunohistochemistry experiments in several cancer tissues. The protein names are mentioned on the top right corner of the figures.

10.7717/peerj.6388/supp-3 Figure S3 GeneCodis annotation of each lncRNA Biological processes and molecular functions predicted by GeneCodis for each lncRNA are shown.

10.7717/peerj.6388/supp-4 Supplemental Information 1 Supplementary Information: Supporting Text and References

## Additional Information and Declarations

### Data Availability

The following information was supplied regarding data availability: The research in this article did not generate any raw data or code. It uses data available in public domain. The raw data is included in the manuscript (in the ‘Materials and Methods’ section and in Supplemental Information) and has been duly credited by naming the source.
