# Supplementary figures and images for: Long non-coding RNAs as pan-cancer master gene regulators of associated protein-coding genes: a systems biology approach
Source: PeerJ. 2019 Feb 20;7:e6388. doi: 10.7717/peerj.6388 (PMC6387586; doi:10.7717/peerj.6388)

Supplementary fig. S1:

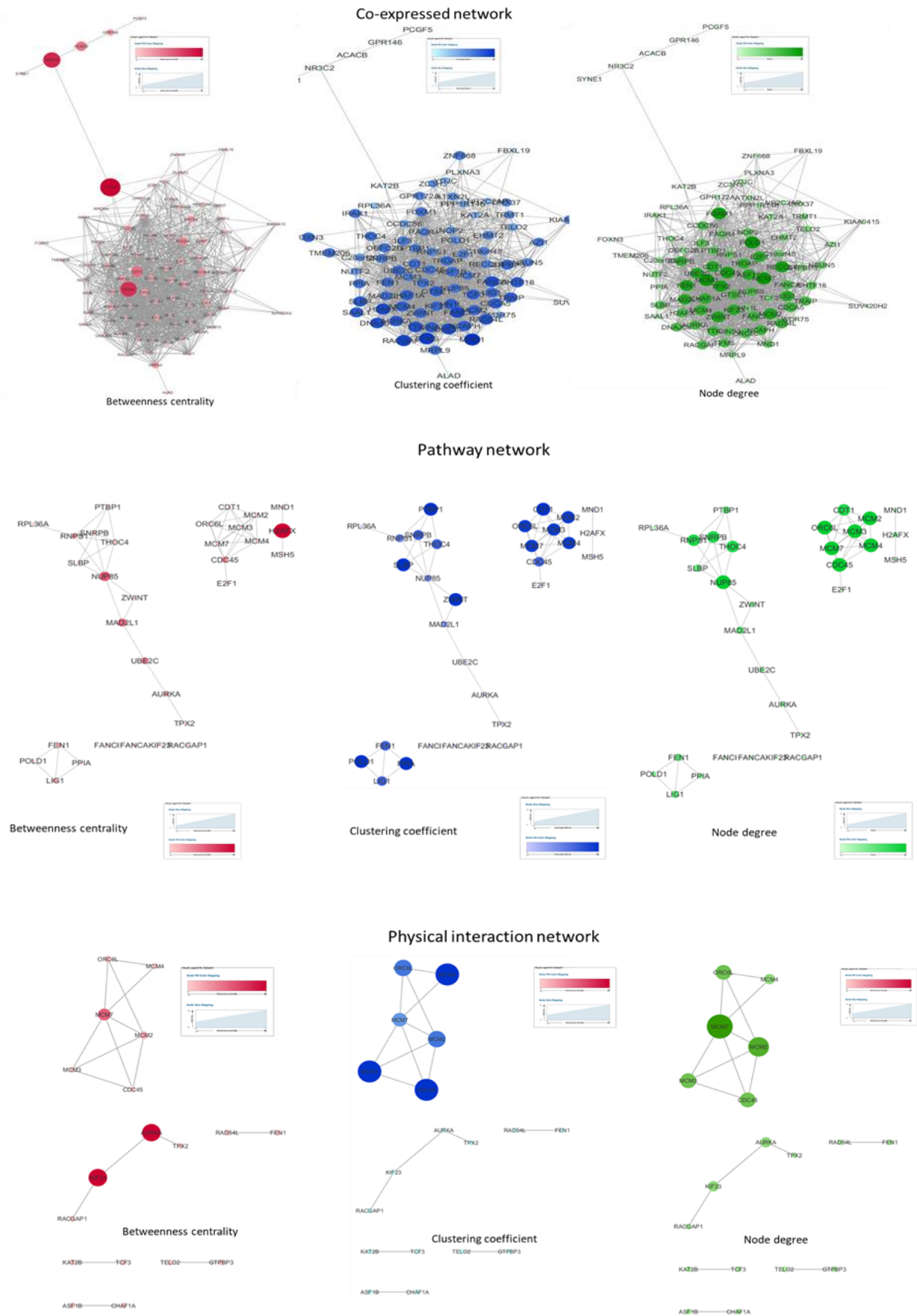

Supplement: Figure S1 — First row shows co-expressed gene networks, second row as pathway gene networks and third row as physically intercating gene networks from our list of significantly differentially expressed genes. [file peerj-07-6388-s001.pdf]

Supplementary fig:

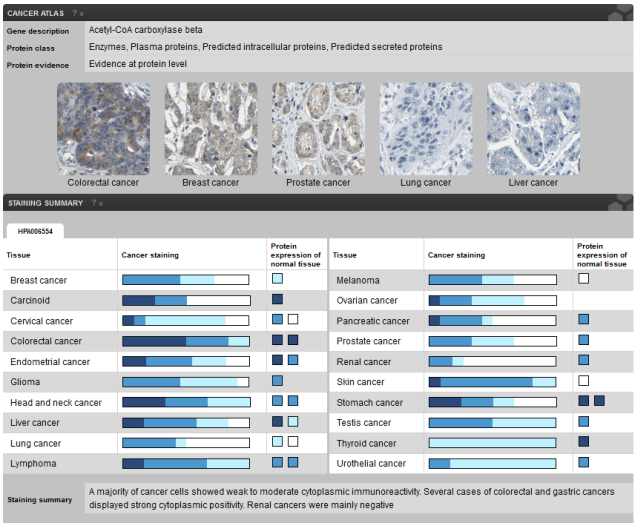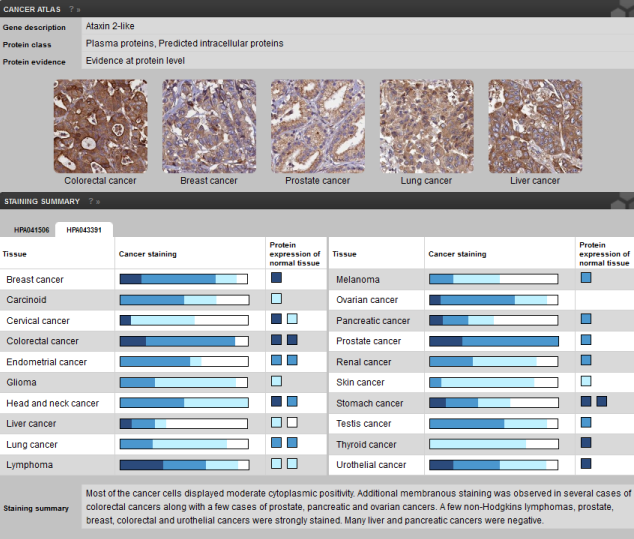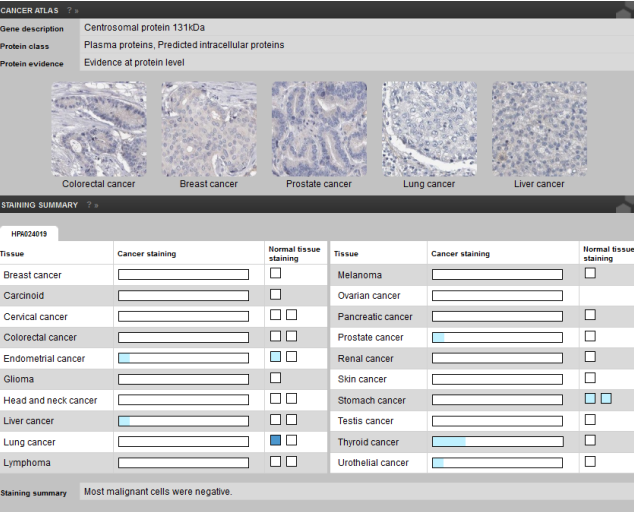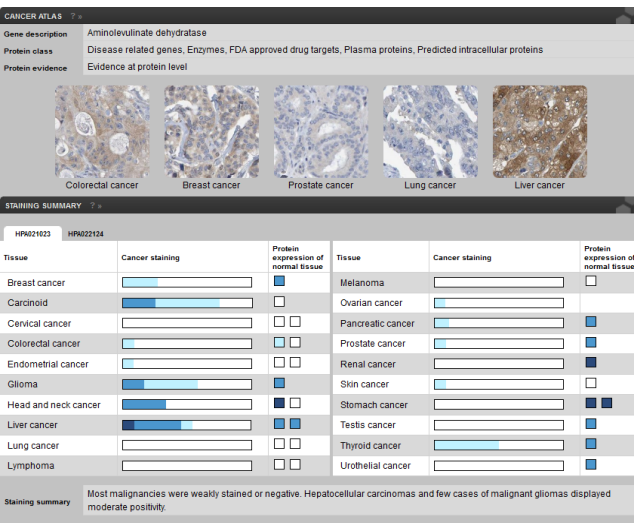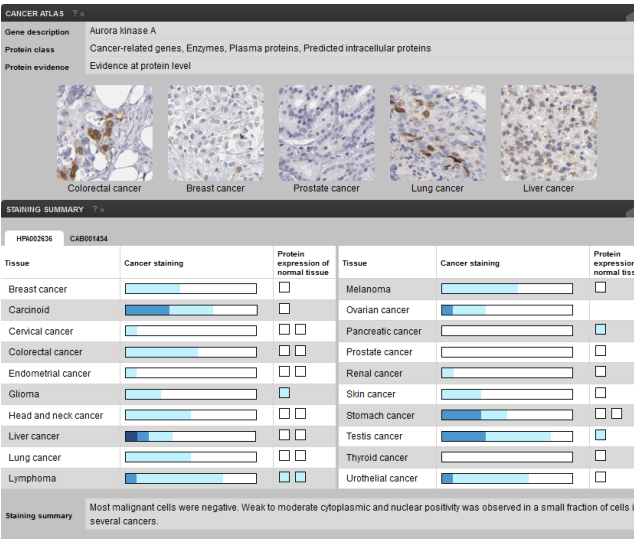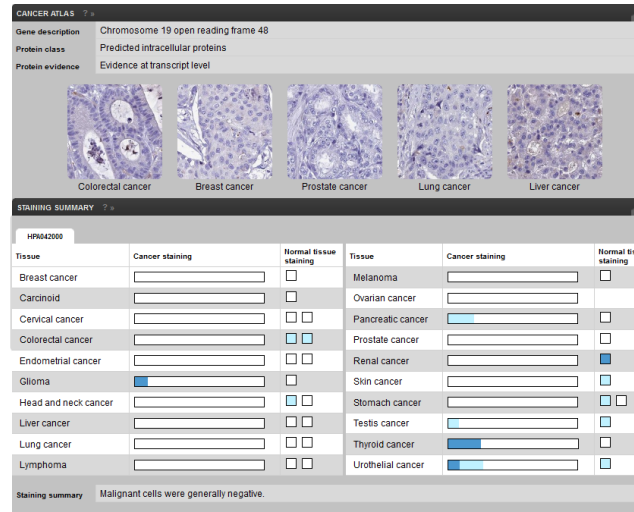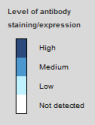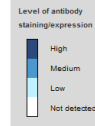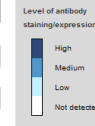

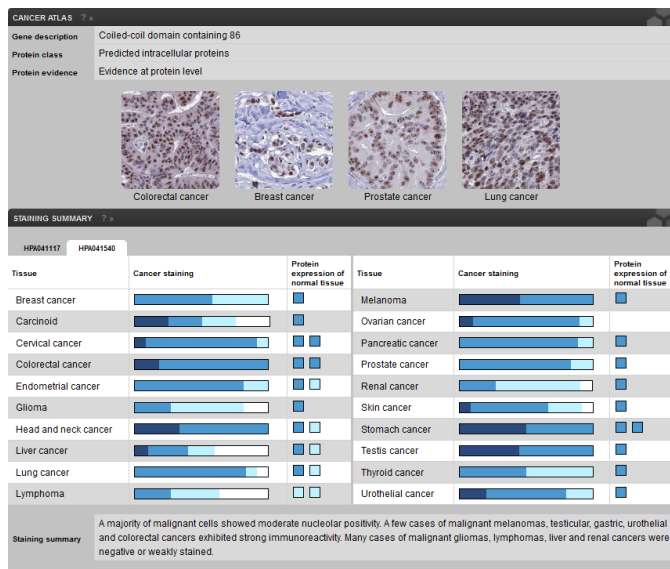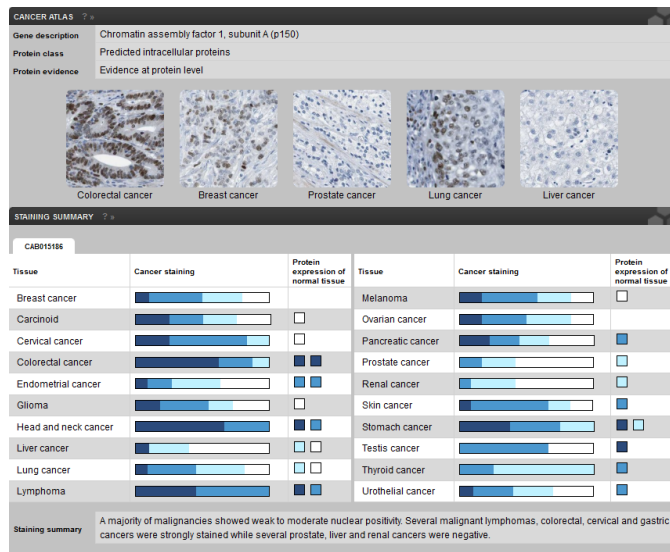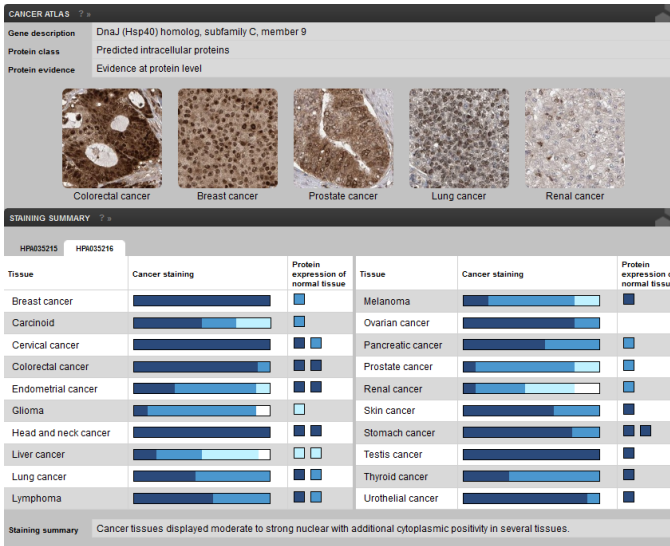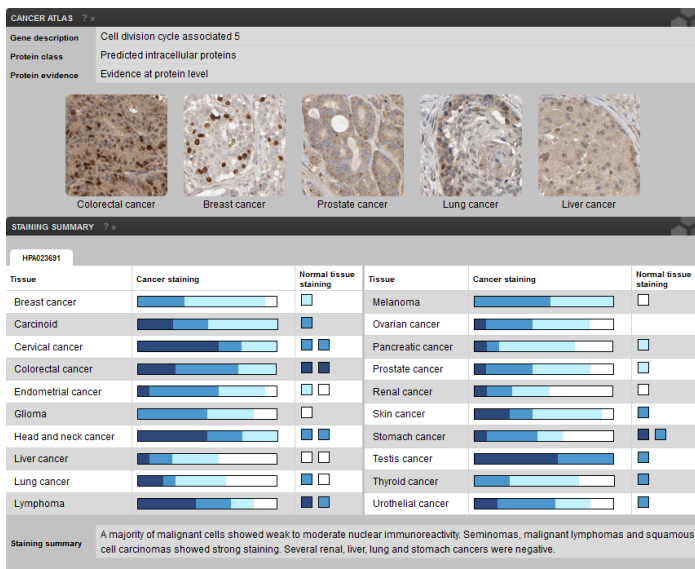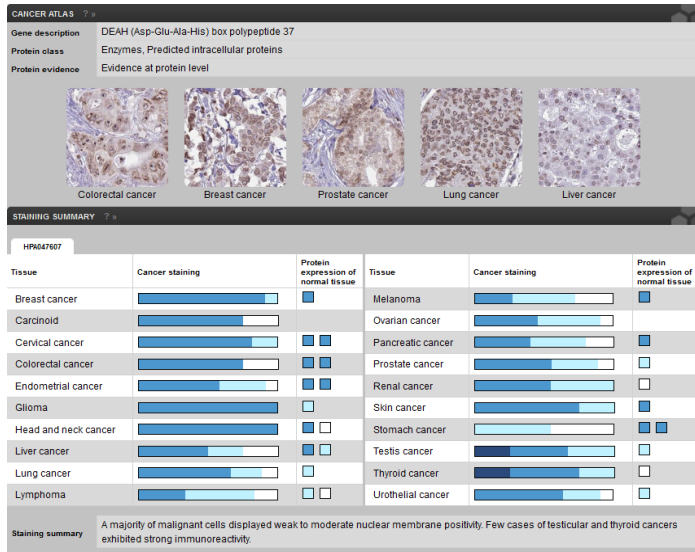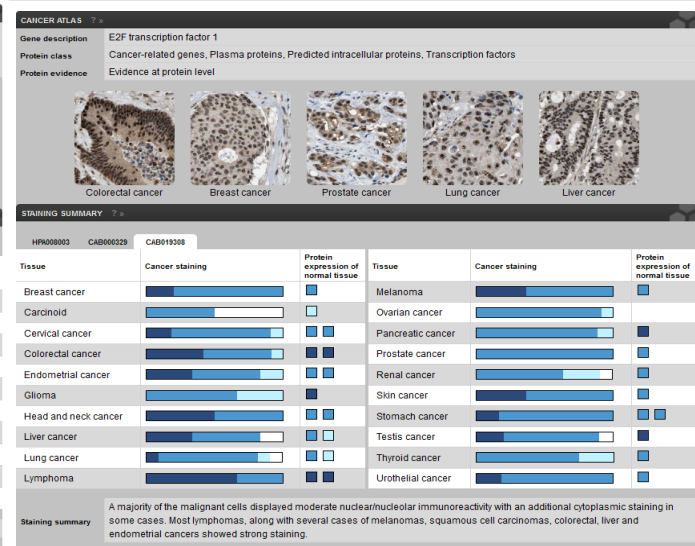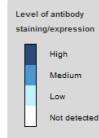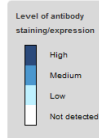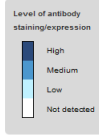

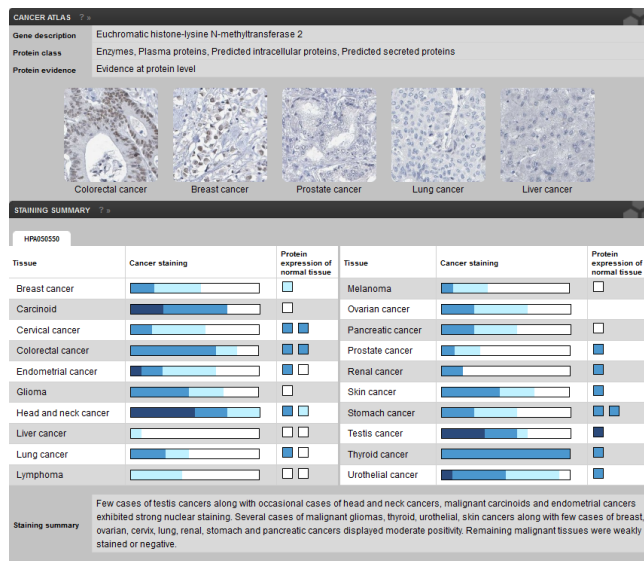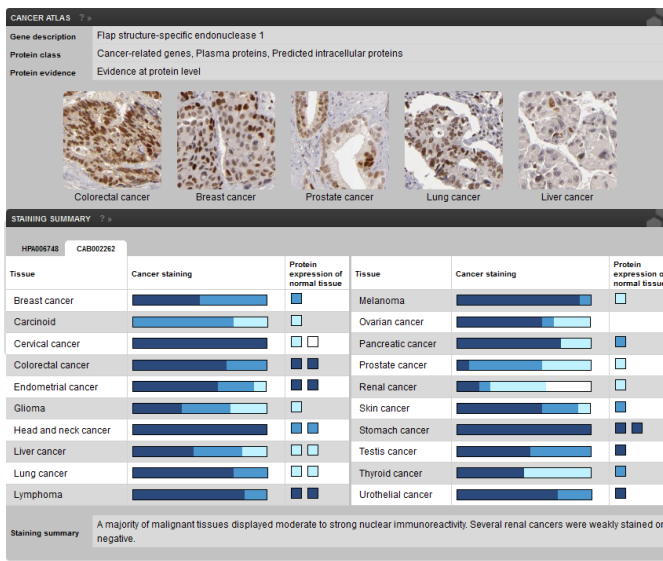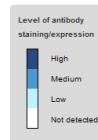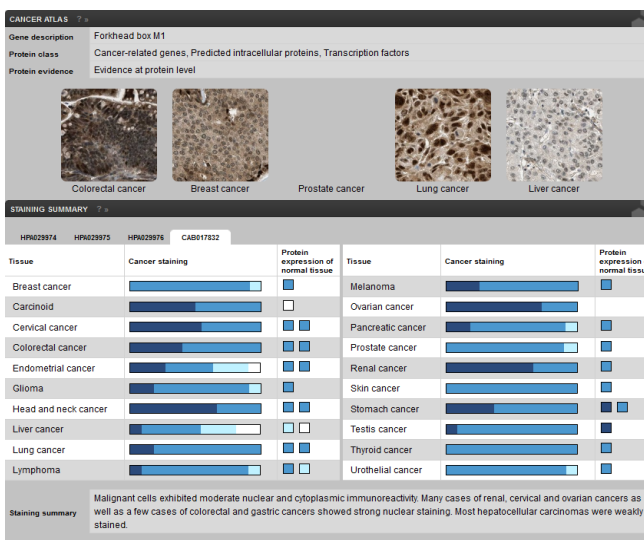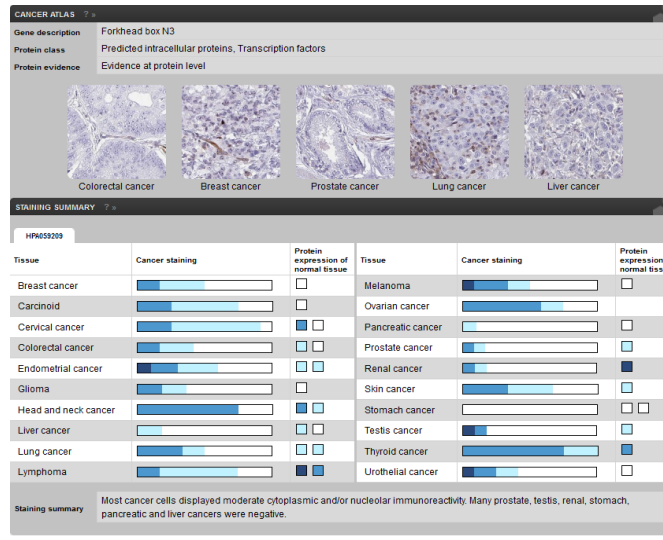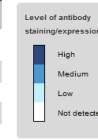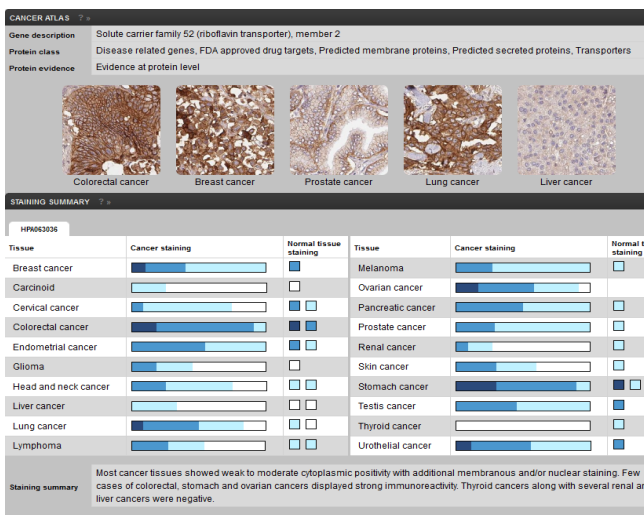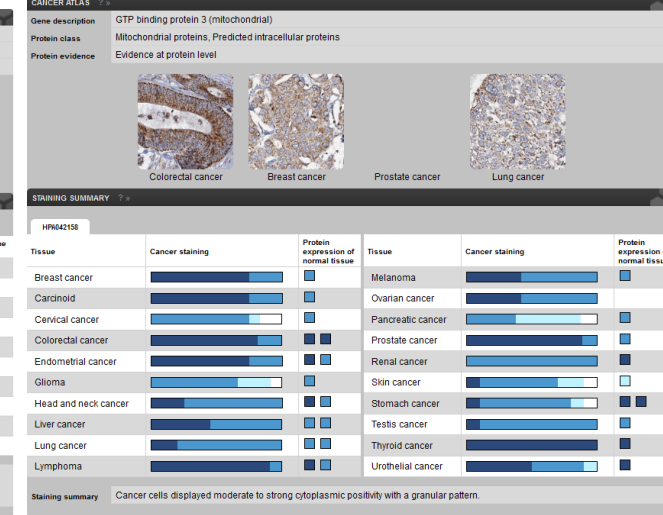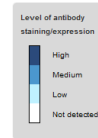

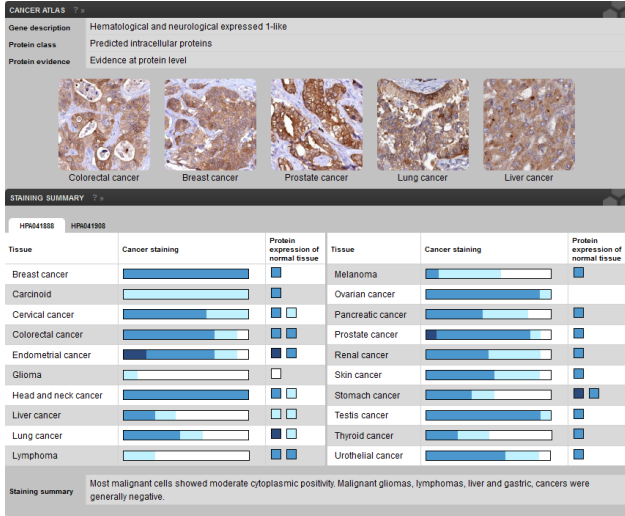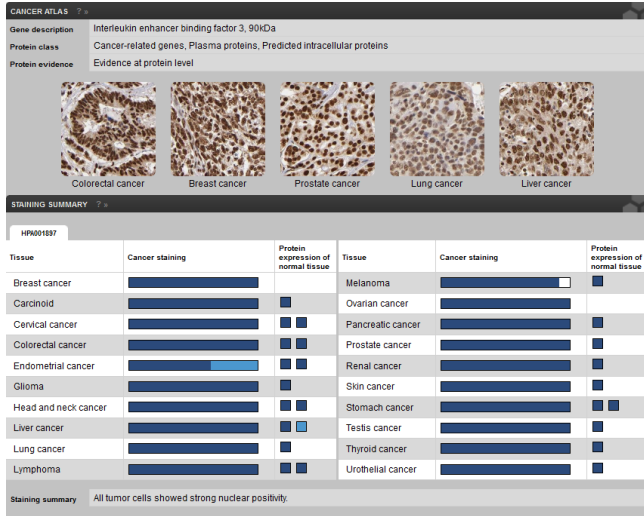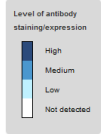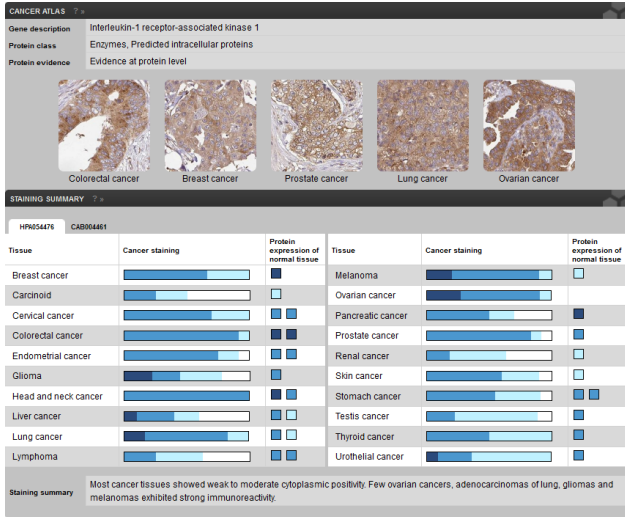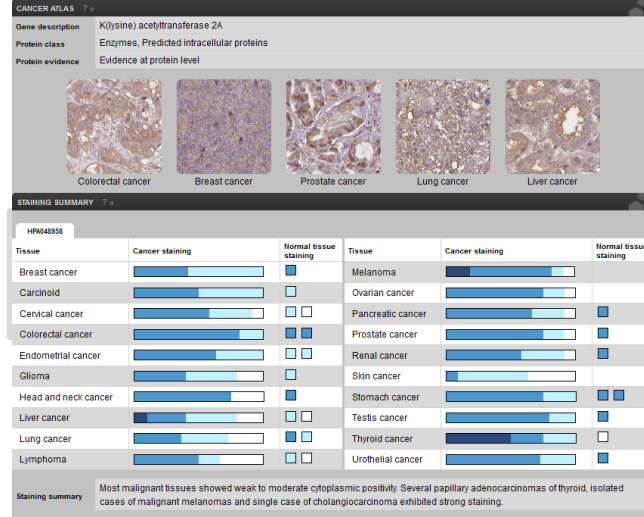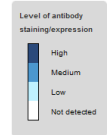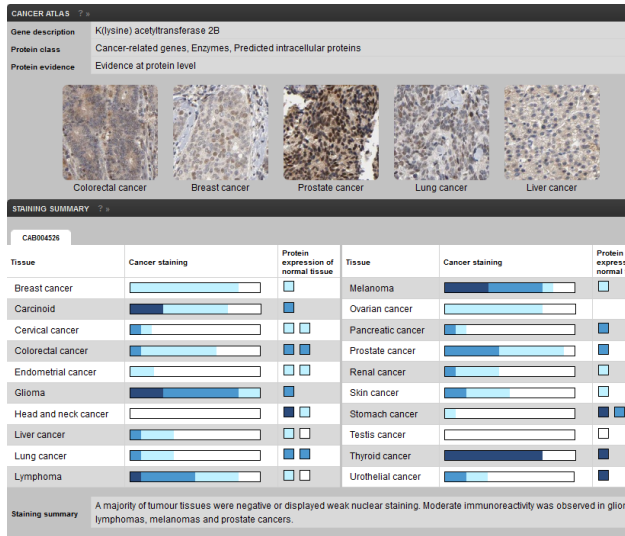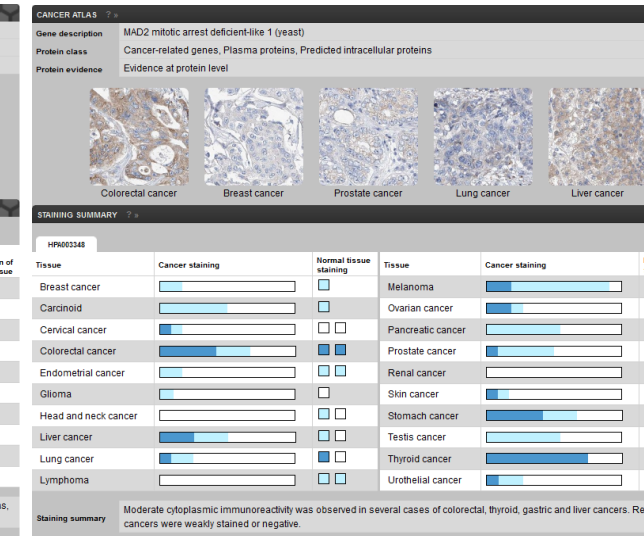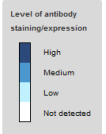

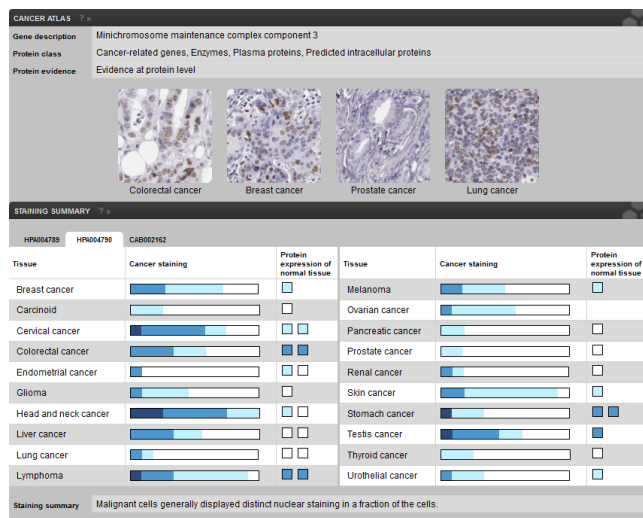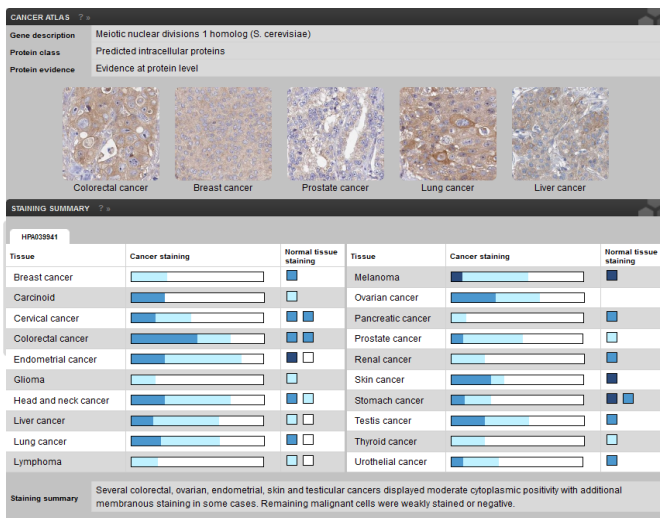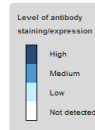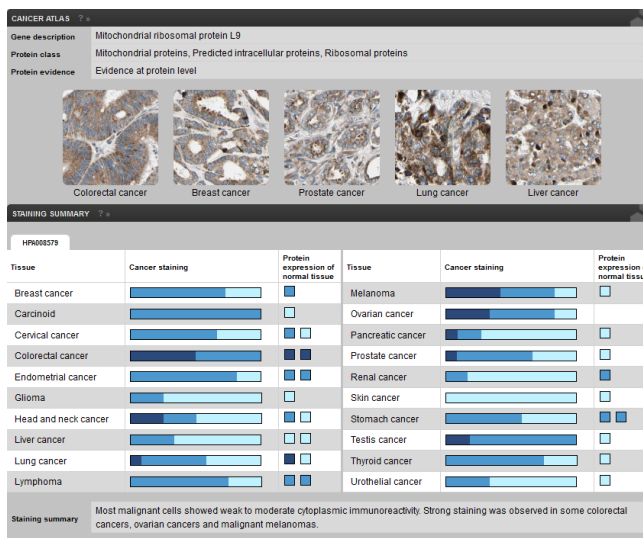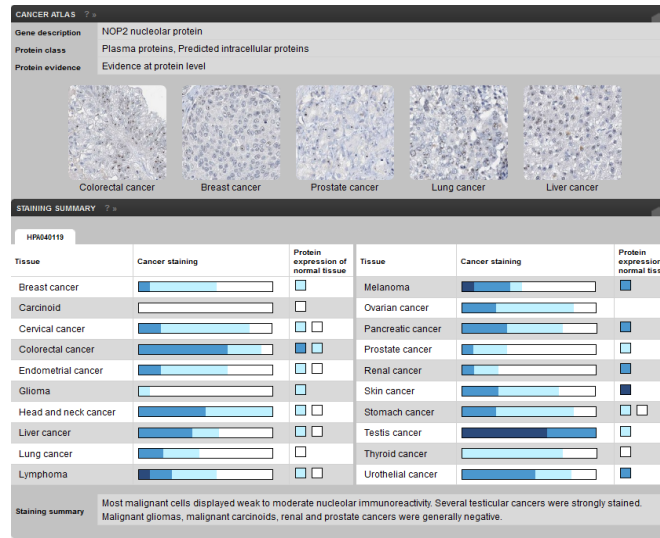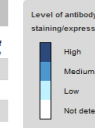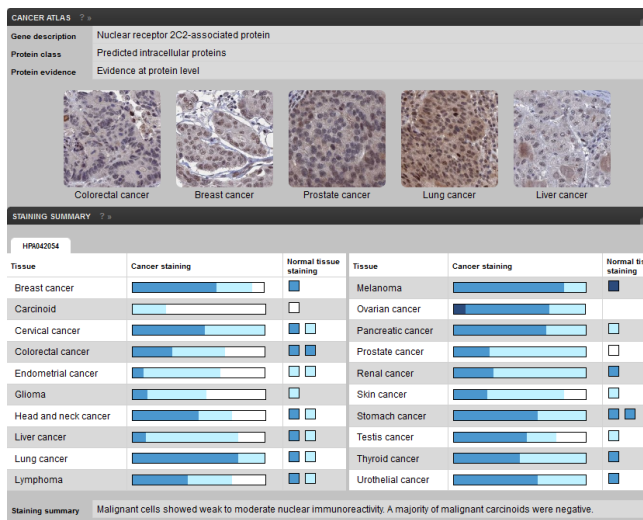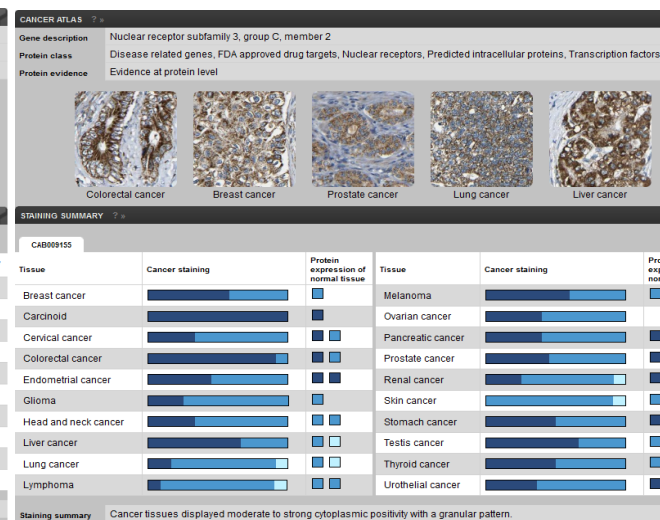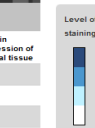

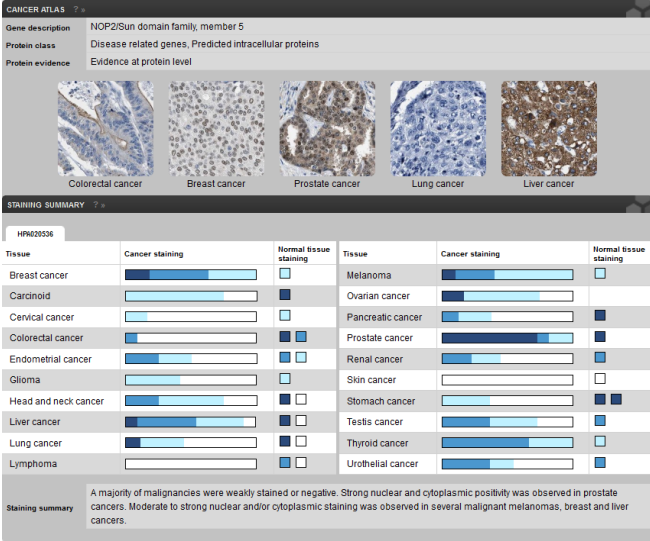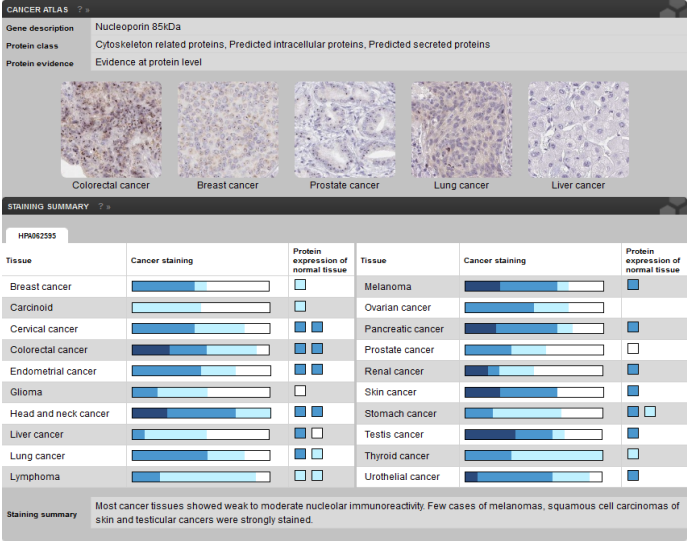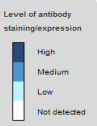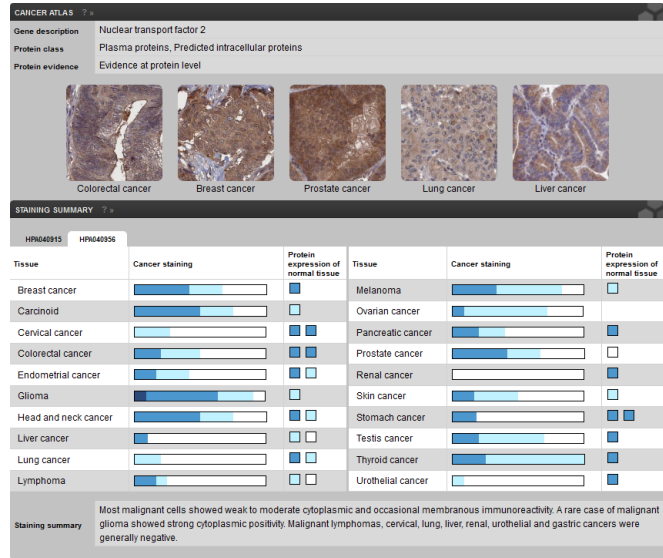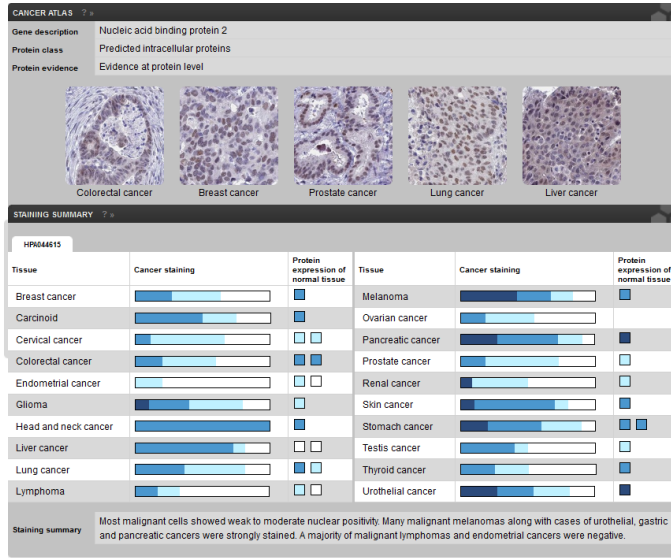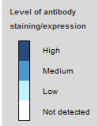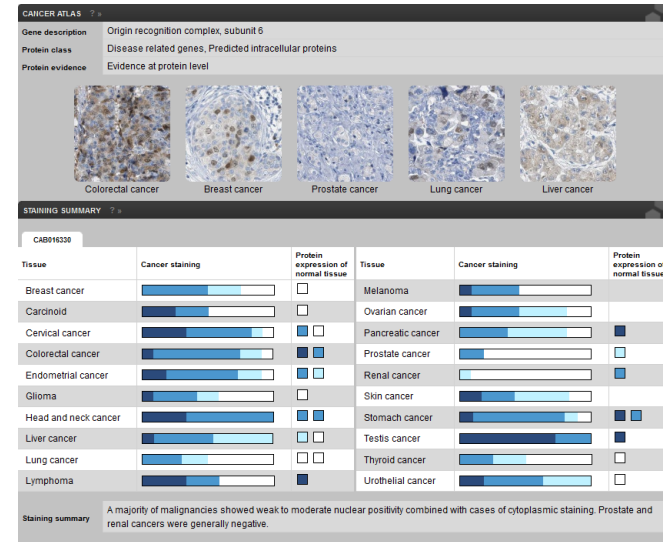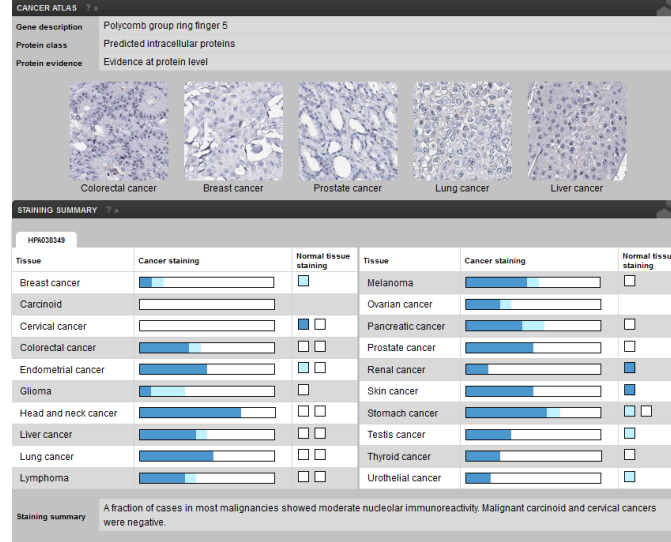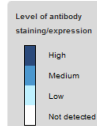

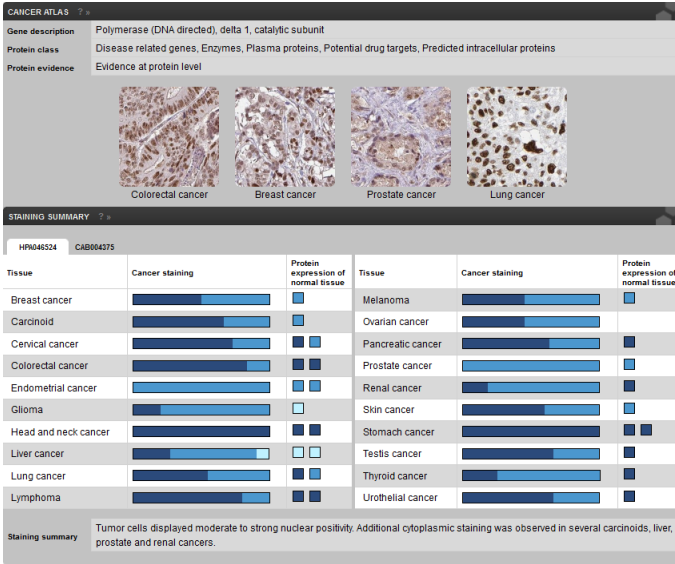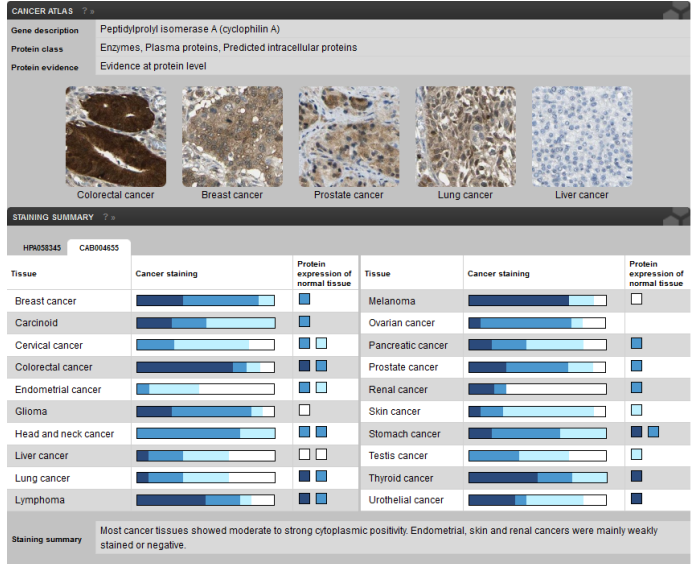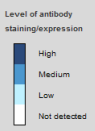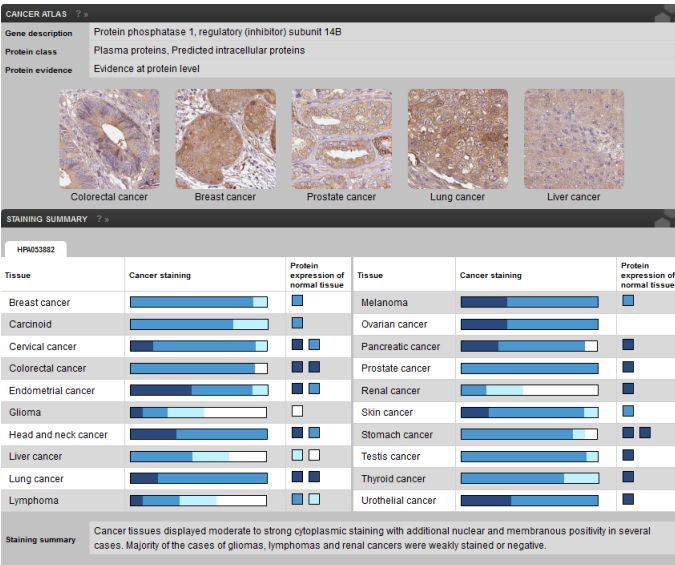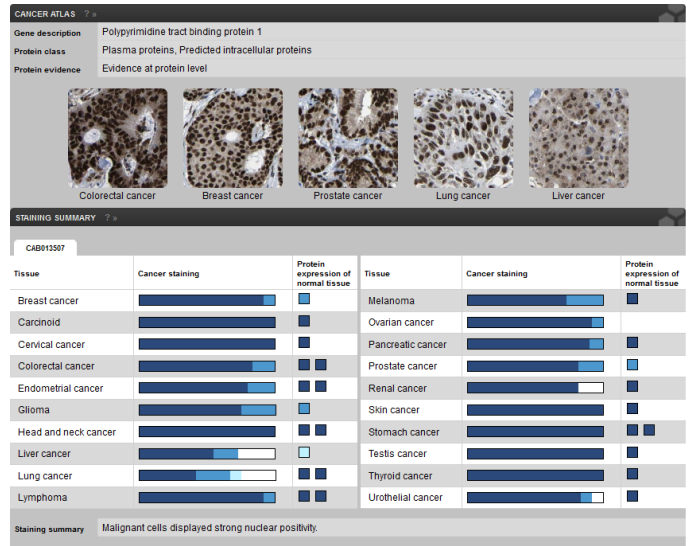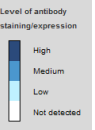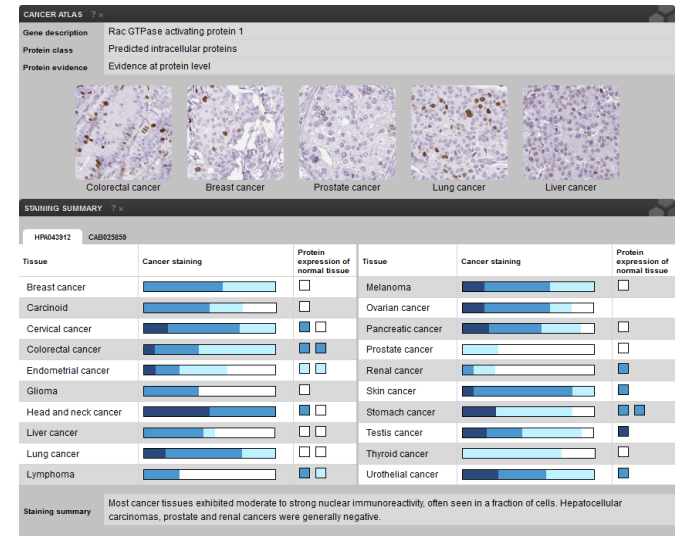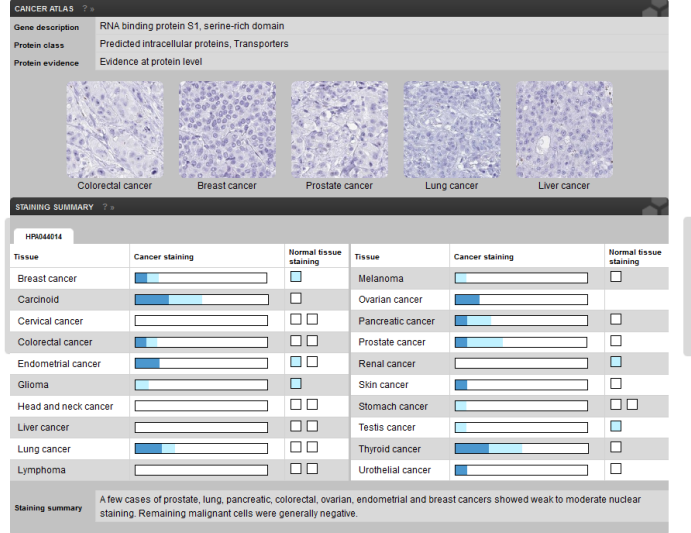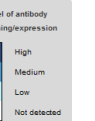

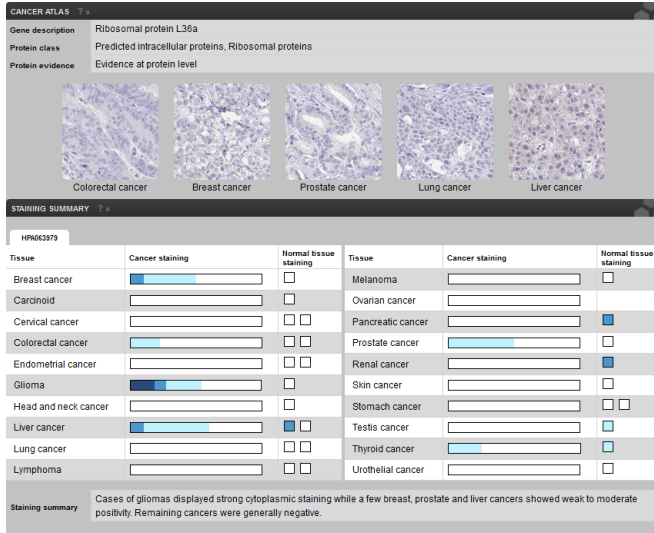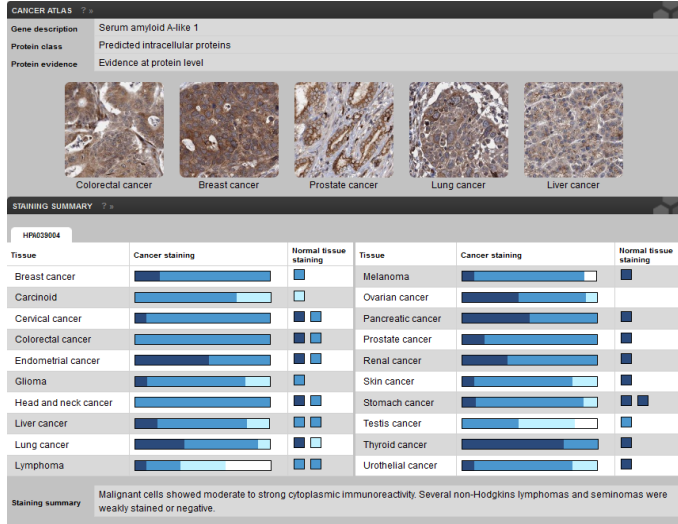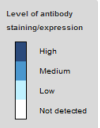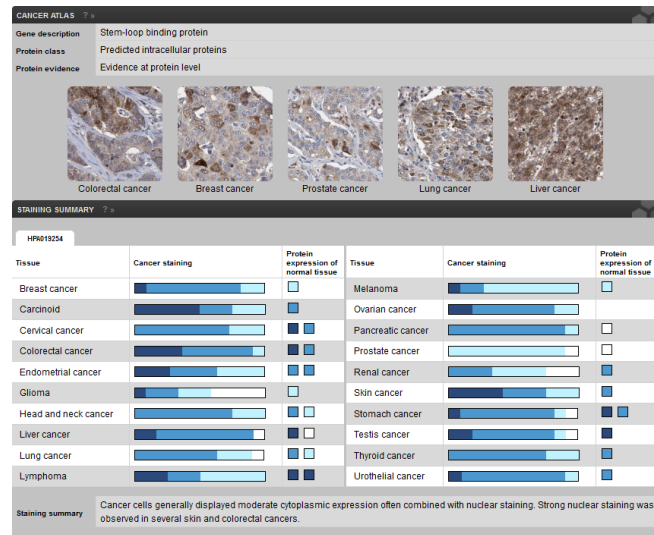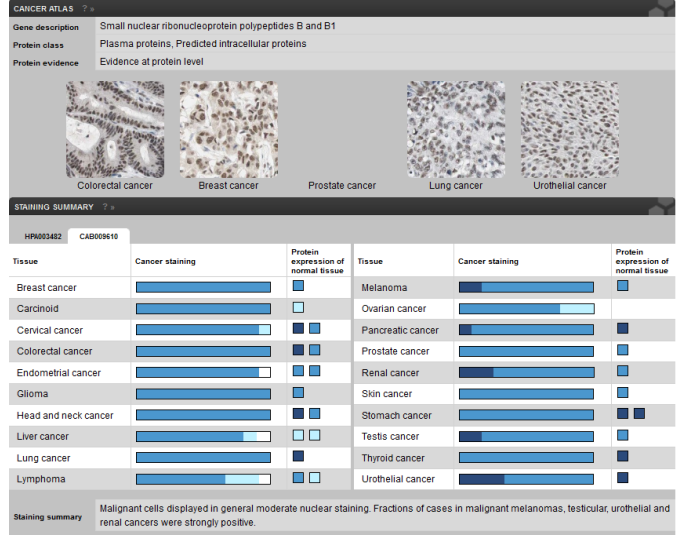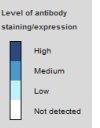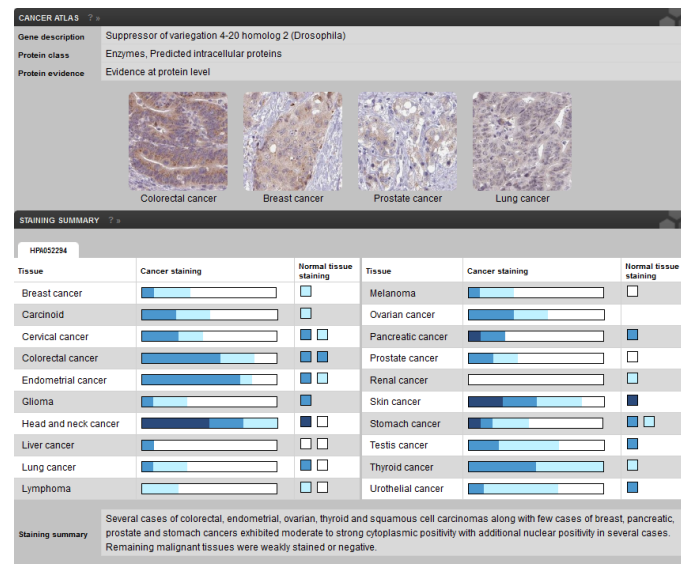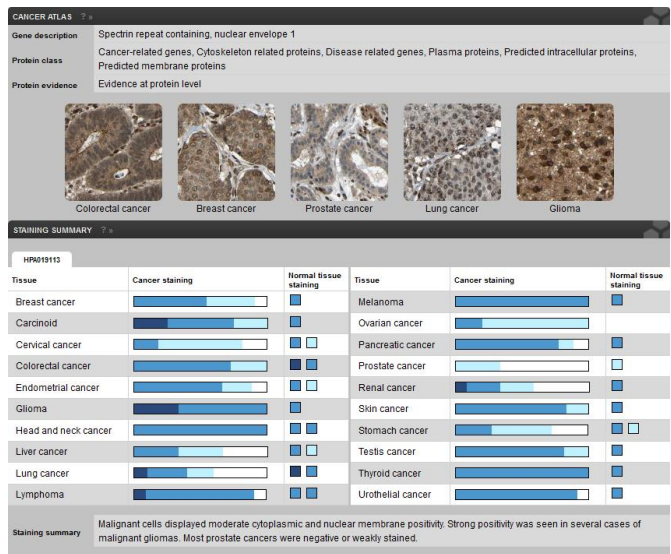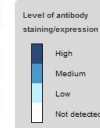

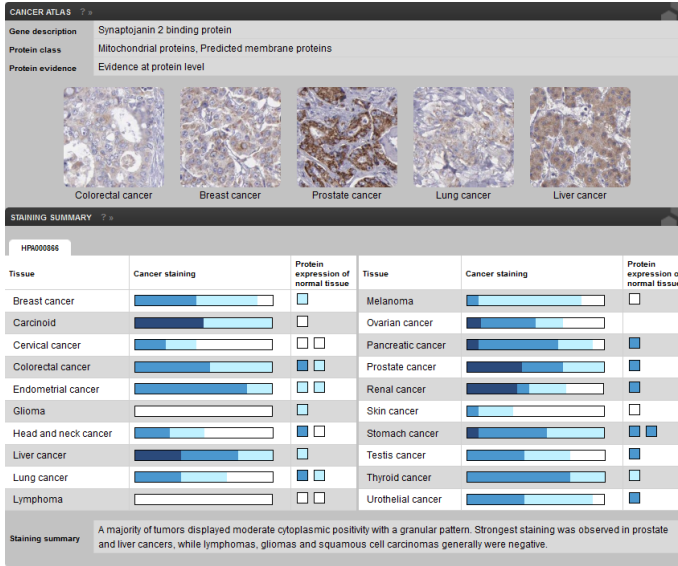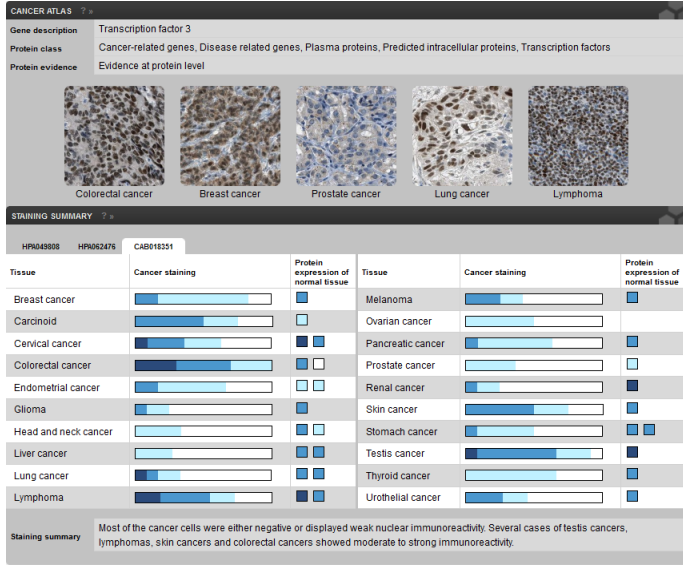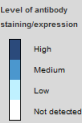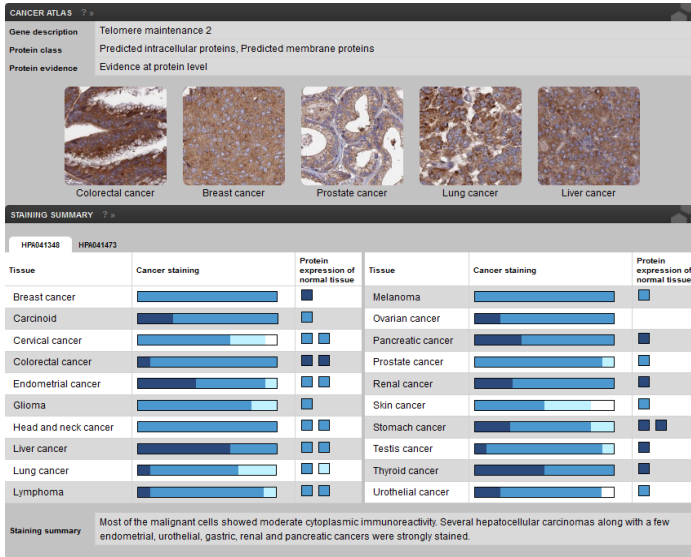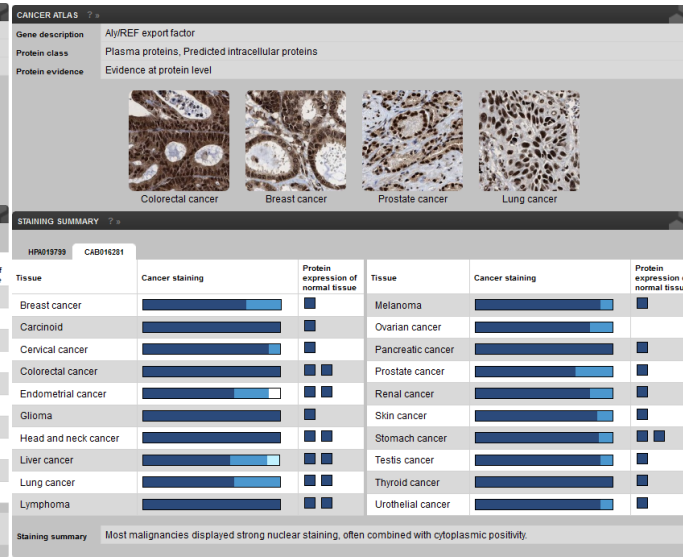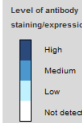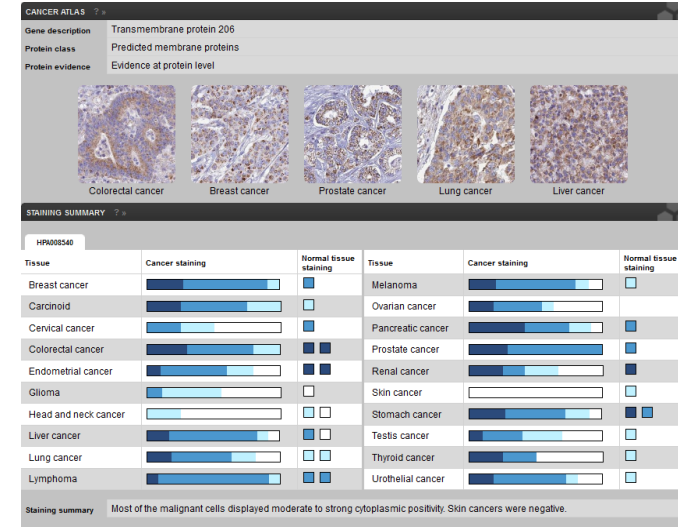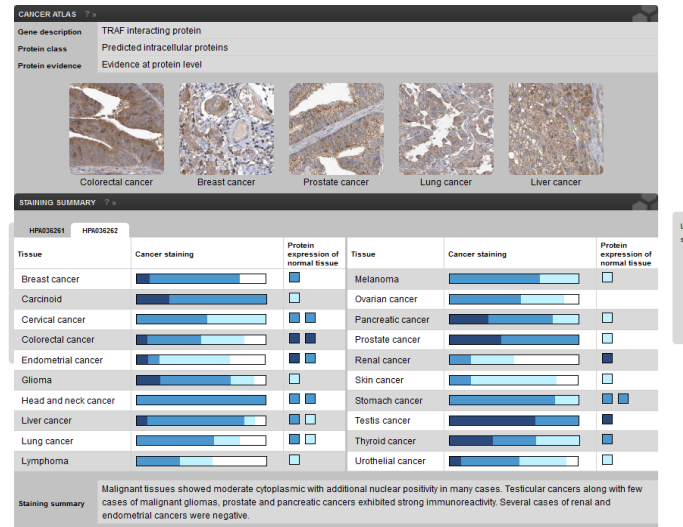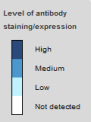

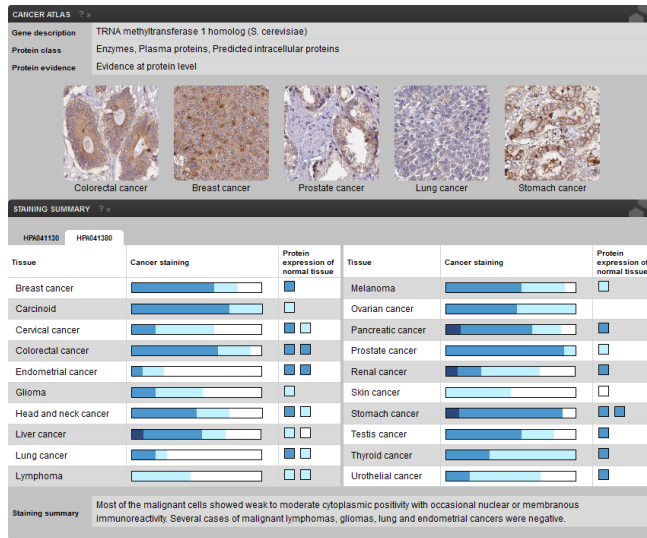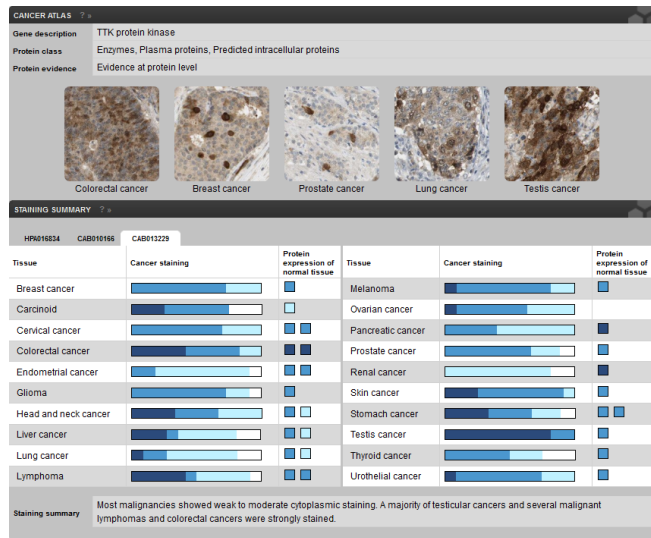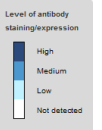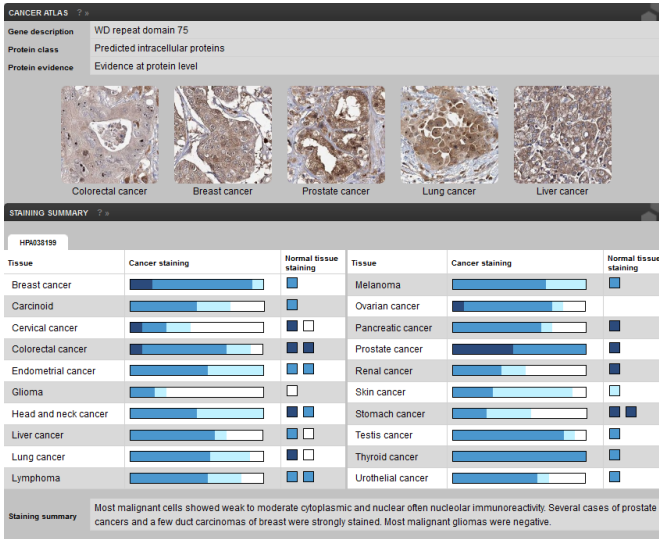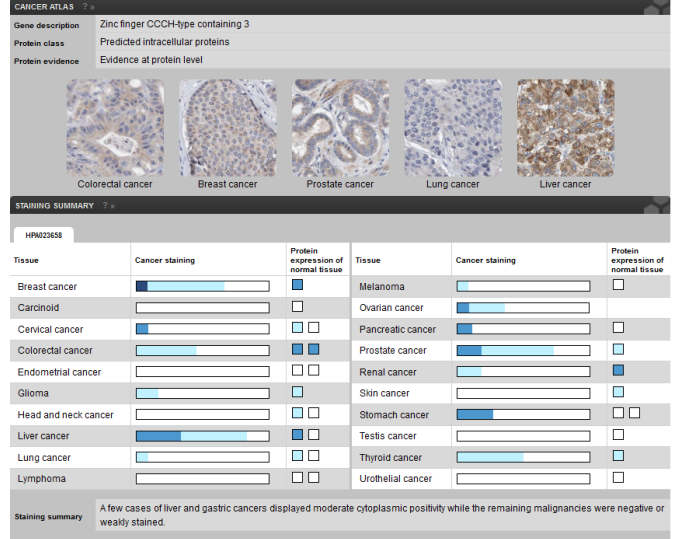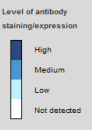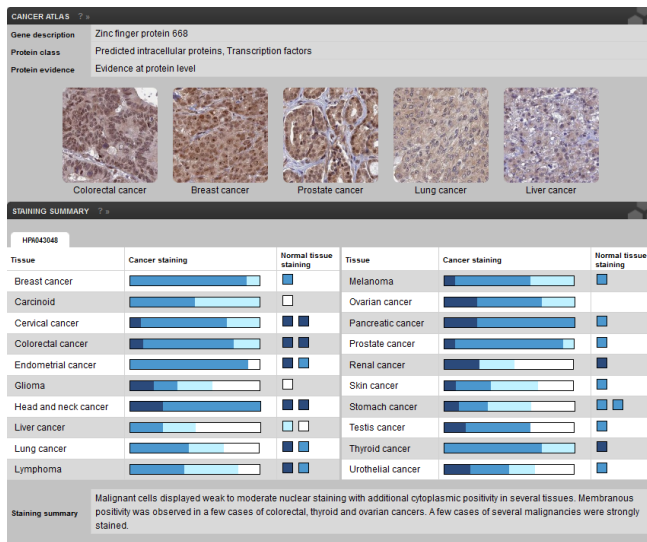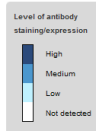

Supplement: Figure S2 — For each protein, protein expression levels are scored based on staining intensity score from immunohistochemistry experiments in several cancer tissues. The protein names are mentioned on the top right corner of the figures. [file peerj-07-6388-s002.pdf]
