# Supplementary material for: Long non-coding RNAs as pan-cancer master gene regulators of associated protein-coding genes: a systems biology approach
Source: PeerJ. 2019 Feb 20;7:e6388. doi: 10.7717/peerj.6388 (PMC6387586; doi:10.7717/peerj.6388)
Supplement: Figure S3 — Biological processes and molecular functions predicted by GeneCodis for each lncRNA are shown. [file peerj-07-6388-s003.pdf]

Supplementary fig. S2:

MAGI2-AS3

BLCA

– Singular Enrichment Analysis of GO Biological Process

axon guidance (BP) platelet activation (BP) intracellular signal transduction (BP) multicellular organismal development (BP) cell differentiation (BP) positive regulation of transcription, DNA-dependent (BP) mitotic cell cycle (BP) positive regulation of transcription from RNA polymerase II promoter (BP) regulation of transcription, DNA-dependent (BP) translational elongation (BP) G2/M transition of mitotic cell cycle (BP) negative regulation of cell proliferation (BP) translation (BP) viral reproduction (BP) innate immune response (BP) cell division (BP) RNA metabolic process (BP) cellular protein metabolic process (BP) interspecies interaction between organisms (BP) cell adhesion (BP) signal transduction (BP) blood coagulation (BP) nerve growth factor receptor signaling pathway (BP) small GTPase mediated signal transduction (BP) regulation of small GTPase mediated signal transduction (BP) mRNA metabolic process (BP) positive regulation of I-kappaB kinase/NF-kappaB cascade (BP) gene expression (BP) apoptotic process (BP) positive regulation of cell proliferation (BP) translational termination (BP)

– Singular Enrichment Analysis of GO Molecular Function

transcription coactivator activity (MF) SH3 domain binding (MF) structural constituent of ribosome (MF) protein homodimerization activity (MF) thyroid hormone receptor binding (MF) GTPase activity (MF) ubiquitin-protein ligase activity (MF) integrin binding (MF) protein binding (MF) DNA binding (MF) protein kinase binding (MF) transcription regulatory region DNA binding (MF) ATP binding (MF) ubiquitin protein ligase binding (MF) GTP binding (MF) glycoprotein binding (MF) guanyl-nucleotide exchange factor activity (MF) RNA polymerase II transcription cofactor activity (MF) signal transducer activity (MF) receptor activity (MF) RNA binding (MF) sequence-specific DNA binding transcription factor activity (MF) binding (MF) transcription cofactor activity (MF) protein heterodimerization activity (MF) Rho guanyl-nucleotide exchange factor activity (MF) calmodulin binding (MF) kinase activity (MF) transcription factor binding (MF) GTPase activator activity (MF) nucleotide binding (MF)

HNSC

– Singular Enrichment Analysis of GO Biological Process

toll-like receptor 1 signaling pathway (BP) mRNA metabolic process (BP) transcription from RNA polymerase II promoter (BP) anti-apoptosis (BP) viral reproduction (BP) intracellular signal transduction (BP) mitotic cell cycle (BP) apoptotic process (BP) blood coagulation (BP) nerve growth factor receptor signaling pathway (BP) toll-like receptor 3 signaling pathway (BP) Toll signaling pathway (BP) transport (BP) cell differentiation (BP) gene expression (BP) cell adhesion (BP) toll-like receptor 4 signaling pathway (BP) small GTPase mediated signal transduction (BP) protein phosphorylation (BP) innate immune response (BP) nervous system development (BP) RNA metabolic process (BP) interspecies interaction between organisms (BP) multicellular organismal development (BP) inflammatory response (BP) stress-activated MAPK cascade (BP) cellular protein metabolic process (BP) negative regulation of apoptotic process (BP) MyD88-independent toll-like receptor signaling pathway (BP) signal transduction (BP) translation (BP)

— Singular Enrichment Analysis of GO Molecular Function

GTPase activator activity (MF) enzyme binding (MF) binding (MF) NADH dehydrogenase (ubiquinone) activity (MF) unfolded protein binding (MF) kinase activity (MF) protein homodimerization activity (MF) protein binding (MF) calcium ion binding (MF) hydrolase activity (MF) protein serine/threonine kinase activity (MF) transcription coactivator activity (MF) transferase activity, transferring phosphorus-containing groups (MF) DNA binding (MF) translation initiation factor activity (MF) voltage-gated ion channel activity (MF) protein heterodimerization activity (MF) nucleotide binding (MF) receptor binding (MF) transferase activity (MF) protein kinase binding (MF) chromatin binding (MF) ATP binding (MF) protein kinase activity (MF) protein domain specific binding (MF) transmembrane signaling receptor activity (MF) structural molecule activity (MF) phosphatase activity (MF) structural constituent of ribosome (MF) thyroid hormone receptor binding (MF) transcription factor binding (MF)

<u>KIRC</u>

— Singular Enrichment Analysis of GO Biological Process

transport (BP) M phase of mitotic cell cycle (BP) transmembrane transport (BP) blood coagulation (BP) viral reproduction (BP) cell cycle checkpoint (BP) gene expression (BP) innate immune response (BP) positive regulation of transcription, DNA-dependent (BP) cellular protein metabolic process (BP) interspecies interaction between organisms (BP) response to drug (BP) positive regulation of transcription from RNA polymerase II promoter (BP) RNA splicing (BP) regulation of apoptotic process (BP) cell division (BP) small GTPase mediated signal transduction (BP) nerve growth factor receptor signaling pathway (BP) transcription from RNA polymerase II promoter (BP) intracellular signal transduction (BP) signal transduction (BP) carbohydrate metabolic process (BP) protein phosphorylation (BP) negative regulation of transcription from RNA polymerase II promoter (BP) mRNA metabolic process (BP) RNA metabolic process (BP) nuclear mRNA splicing, via spliceosome (BP) mitotic prometaphase (BP) mitotic cell cycle (BP) regulation of small GTPase mediated signal transduction (BP) apoptotic process (BP)

— Singular Enrichment Analysis of GO Molecular Function

protein tyrosine kinase activity (MF) DNA binding (MF) GTPase activity (MF) transferase activity, transferring glycosyl groups (MF) protein serine/threonine kinase activity (MF) magnesium ion binding (MF) transferase activity, transferring phosphorus-containing groups (MF) DNA-directed RNA polymerase activity (MF) nucleotide binding (MF) protein homodimerization activity (MF) protein binding (MF) structural constituent of ribosome (MF) identical protein binding (MF) protein kinase binding (MF) GTPase activator activity (MF) ATP binding (MF) lyase activity (MF) protein C-terminus binding (MF) kinase activity (MF) transcription coactivator activity (MF) protein kinase activity (MF) Rho guanyl-nucleotide exchange factor activity (MF) protein domain specific binding (MF) hydrogen ion transmembrane transporter activity (MF) RNA binding (MF) transcription corepressor activity (MF) TBP-class protein binding (MF) transcription regulatory region DNA binding (MF) SH3 domain binding (MF) protein tyrosine kinase activator activity (MF) lamin binding (MF)

<u>KIRP</u>

— Singular Enrichment Analysis of GO Biological Process

axon guidance (BP) blood coagulation (BP) leukocyte migration (BP) viral reproduction (BP) response to drug (BP) nerve growth factor receptor signaling pathway (BP) mitotic cell cycle (BP) RNA splicing (BP) interspecies interaction between organisms (BP) cell cycle (BP) DNA repair (BP) mRNA metabolic process (BP) GTP catabolic process (BP) protein transport (BP) cellular membrane organization (BP) nervous system development (BP) G1/S transition of mitotic cell cycle (BP) positive regulation of transcription, DNA-dependent (BP) protein phosphorylation (BP) transmembrane transport (BP) G2/M transition of mitotic cell cycle (BP) apoptotic process (BP) cell division (BP) RNA metabolic process (BP) regulation of transcription, DNA-dependent (BP) cellular protein metabolic process (BP) gene expression (BP) translation (BP) signal transduction (BP) synaptic transmission (BP) positive regulation of transcription from RNA polymerase II promoter (BP)

– Singular Enrichment Analysis of GO Molecular Function

signal transducer activity (MF) CD8 receptor binding (MF) receptor activity (MF) protein kinase activity (MF) transcription factor binding (MF) nucleotide binding (MF) protein serine/threonine kinase activity (MF) sequence-specific DNA binding transcription factor activity (MF) protein tyrosine kinase activity (MF) ATP binding (MF) RNA binding (MF) phosphoprotein binding (MF) structural constituent of ribosome (MF) hydrogen ion transmembrane transporter activity (MF) protein binding (MF) phosphatidylinositol phospholipase C activity (MF) calcium- and calmodulin-responsive adenylate cyclase activity (MF) 5'-deoxyribose-5-phosphate lyase activity (MF) GTPase activity (MF) GTP binding (MF) DNA binding (MF) cyclin-dependent protein kinase activity (MF) inward rectifier potassium channel activity (MF) non-membrane spanning protein tyrosine kinase activity (MF) kinase activity (MF) protein C-terminus binding (MF) glycoprotein binding (MF) RNA polymerase II transcription cofactor activity (MF) syntaxin binding (MF) magnesium ion binding (MF) adenylate cyclase activity (MF)

LIHC

– Singular Enrichment Analysis of GO Biological Process

regulation of apoptotic process (BP) RNA splicing (BP) positive regulation of transcription from RNA polymerase II promoter (BP) viral reproduction (BP) M/G1 transition of mitotic cell cycle (BP) G1/S transition of mitotic cell cycle (BP) RNA metabolic process (BP) blood coagulation (BP) cellular nitrogen compound metabolic process (BP) regulation of transcription, DNA-dependent (BP) translation (BP) S phase of mitotic cell cycle (BP) innate immune response (BP) transcription from RNA polymerase II promoter (BP) mRNA metabolic process (BP) protein phosphorylation (BP) cell cycle checkpoint (BP) intracellular signal transduction (BP) apoptotic process (BP) signal transduction (BP) cellular protein metabolic process (BP) small GTPase mediated signal transduction (BP) axon guidance (BP) interspecies interaction between organisms (BP) insulin receptor signaling pathway (BP) regulation of small GTPase mediated signal transduction (BP) mitotic cell cycle (BP) nerve growth factor receptor signaling pathway (BP) cell proliferation (BP) antigen processing and presentation of peptide antigen via MHC class I (BP) gene expression (BP)

– Singular Enrichment Analysis of GO Molecular Function

transcription factor binding (MF) GTP binding (MF) protein domain specific binding (MF) Rho guanyl-nucleotide exchange factor activity (MF) translation initiation factor activity (MF) catalytic activity (MF) signal transducer activity (MF) chromatin binding (MF) guanyl-nucleotide exchange factor activity (MF) protein N-terminus binding (MF) structural constituent of ribosome (MF) calcium ion binding (MF) protein kinase binding (MF) transferase activity (MF) GTPase activity (MF) protein heterodimerization activity (MF) DNA binding (MF) protein kinase activity (MF) transcription regulatory region DNA binding (MF) nucleotide binding (MF) insulin receptor binding (MF) RNA polymerase II transcription cofactor activity (MF) protein serine/threonine kinase activity (MF) RNA binding (MF) transcription coactivator activity (MF) enzyme binding (MF) receptor activity (MF) structural molecule activity (MF) actin binding (MF) protein binding (MF) ATP binding (MF)

LUAD

– Singular Enrichment Analysis of GO Biological Process

translation (BP) negative regulation of cell proliferation (BP) positive regulation of cell proliferation (BP) positive regulation of transcription from RNA polymerase II promoter (BP) platelet activation (BP) mitotic cell cycle (BP) small GTPase mediated signal transduction (BP) positive regulation of transcription, DNA-dependent (BP) apoptotic process (BP) gene expression (BP) blood coagulation (BP) RNA metabolic process (BP) mRNA metabolic process (BP) protein phosphorylation (BP) transcription from RNA polymerase II promoter (BP) viral reproduction (BP) cell cycle (BP) insulin receptor signaling pathway (BP) axon guidance (BP) activation of phospholipase C activity (BP) GTP catabolic process (BP) transmembrane transport (BP) DNA repair (BP) viral infectious cycle (BP) signal transduction (BP) intracellular signal transduction (BP) interspecies interaction between organisms (BP) cellular protein metabolic process (BP) synaptic transmission (BP) innate immune response (BP) nerve growth factor receptor signaling pathway (BP)

− Singular Enrichment Analysis of GO Molecular Function

DNA-directed DNA polymerase activity (MF) ATP binding (MF) transcription factor binding (MF) metal ion binding (MF) collagen binding (MF) transcription coactivator activity (MF) RNA binding (MF) GTPase activity (MF) NADH dehydrogenase (ubiquinone) activity (MF) nucleotide binding (MF) drug binding (MF) transferase activity, transferring phosphorus-containing groups (MF) hydrolase activity (MF) protein binding (MF) protein kinase binding (MF) protein C-terminus binding (MF) sequence-specific distal enhancer binding RNA polymerase II transcription factor activity (MF) protein kinase activity (MF) DNA binding (MF) nucleotidyltransferase activity (MF) ubiquitin protein ligase binding (MF) GTP binding (MF) receptor signaling protein activity (MF) adenylate cyclase activity (MF) identical protein binding (MF) chromatin binding (MF) enzyme binding (MF) structural constituent of ribosome (MF) protein serine/threonine kinase activity (MF) protein homodimerization activity (MF) magnesium ion binding (MF)

<u>LUSC</u>

− Singular Enrichment Analysis of GO Biological Process

transmembrane transport (BP) cell cycle checkpoint (BP) regulation of transcription, DNA-dependent (BP) mRNA metabolic process (BP) positive regulation of transcription from RNA polymerase II promoter (BP) leukocyte migration (BP) viral reproduction (BP) blood coagulation (BP) G1/S transition of mitotic cell cycle (BP) nerve growth factor receptor signaling pathway (BP) G2/M transition of mitotic cell cycle (BP) transcription from RNA polymerase II promoter (BP) mitotic prometaphase (BP) interspecies interaction between organisms (BP) M phase of mitotic cell cycle (BP) mitotic cell cycle (BP) small GTPase mediated signal transduction (BP) induction of apoptosis by extracellular signals (BP) signal transduction (BP) regulation of small GTPase mediated signal transduction (BP) cellular protein metabolic process (BP) positive regulation of transcription, DNA-dependent (BP) RNA splicing (BP) platelet activation (BP) RNA metabolic process (BP) gene expression (BP) DNA repair (BP) cell division (BP) negative regulation of cell proliferation (BP) apoptotic process (BP) translation (BP)

− Singular Enrichment Analysis of GO Molecular Function

hydrogen-exporting ATPase activity, phosphorylative mechanism (MF) SH3/SH2 adaptor activity (MF) binding (MF) protein homodimerization activity (MF) structural constituent of ribosome (MF) protein binding (MF) histone acetyltransferase activity (MF) GTPase activity (MF) transcription regulatory region DNA binding (MF) hydrogen ion transmembrane transporter activity (MF) protein serine/threonine kinase activity (MF) Rho guanyl-nucleotide exchange factor activity (MF) protein kinase binding (MF) ATP binding (MF) P-P-bond-hydrolysis-driven protein transmembrane transporter activity (MF) guanyl-nucleotide exchange factor activity (MF) nucleotide binding (MF) protein N-terminus binding (MF) DNA binding (MF) GTP binding (MF) motor activity (MF) damaged DNA binding (MF) protein kinase activity (MF) translation initiation factor activity (MF) RNA binding (MF) endopeptidase activity (MF) microtubule motor activity (MF) transcription factor binding (MF) growth factor activity (MF) peptidase activity (MF) chromatin binding (MF)

PRAD

— Singular Enrichment Analysis of GO Biological Process

activation of phospholipase C activity (BP) innate immune response (BP) immune response (BP) cell division (BP) nucleotide-excision repair (BP) G2/M transition of mitotic cell cycle (BP) intracellular signal transduction (BP) axon guidance (BP) small GTPase mediated signal transduction (BP) protein phosphorylation (BP) viral reproduction (BP) protein folding (BP) transcription from RNA polymerase II promoter (BP) signal transduction (BP) mitotic cell cycle (BP) carbohydrate metabolic process (BP) nuclear mRNA splicing, via spliceosome (BP) regulation of small GTPase mediated signal transduction (BP) mitosis (BP) cell cycle (BP) gene expression (BP) blood coagulation (BP) nerve growth factor receptor signaling pathway (BP) cell proliferation (BP) translation (BP) inflammatory response (BP) synaptic transmission (BP) metabolic process (BP) DNA repair (BP) cellular protein metabolic process (BP) RNA splicing (BP)

— Singular Enrichment Analysis of GO Molecular Function

receptor activity (MF) GTPase activity (MF) protein C-terminus binding (MF) nucleotide binding (MF) kinase activity (MF) protein heterodimerization activity (MF) structural constituent of ribosome (MF) protein N-terminus binding (MF) single-stranded DNA binding (MF) protein binding (MF) Rho guanyl-nucleotide exchange factor activity (MF) ubiquitin protein ligase binding (MF) protein kinase binding (MF) unfolded protein binding (MF) damaged DNA binding (MF) protein kinase activity (MF) hydrolase activity (MF) DNA-directed RNA polymerase activity (MF) protein serine/threonine kinase activity (MF) structural molecule activity (MF) enzyme binding (MF) protein domain specific binding (MF) ATP binding (MF) RNA binding (MF) guanyl-nucleotide exchange factor activity (MF) chromatin binding (MF) DNA binding (MF) calmodulin binding (MF) actin binding (MF) transcription coactivator activity (MF) magnesium ion binding (MF)

MIR22HG

BLCA

— Singular Enrichment Analysis of GO Biological Process

intracellular signal transduction (BP) regulation of small GTPase mediated signal transduction (BP) mRNA metabolic process (BP) transport (BP) viral reproduction (BP) positive regulation of transcription from RNA polymerase II promoter (BP) multicellular organismal development (BP) Toll signaling pathway (BP) gene expression (BP) toll-like receptor 4 signaling pathway (BP) nuclear mRNA splicing, via spliceosome (BP) toll-like receptor 1 signaling pathway (BP) RNA metabolic process (BP) cell division (BP) mitotic cell cycle (BP) signal transduction (BP) small GTPase mediated signal transduction (BP) G-protein coupled receptor signaling pathway (BP) innate immune response (BP) nerve growth factor receptor signaling pathway (BP) RNA splicing (BP) negative regulation of cell proliferation (BP) cell cycle (BP) apoptotic process (BP) protein phosphorylation (BP) blood coagulation (BP) transcription from RNA polymerase II promoter (BP) toll-like receptor signaling pathway (BP) axon guidance (BP) toll-like receptor 2 signaling pathway (BP) MyD88-dependent toll-like receptor signaling pathway (BP)

— Singular Enrichment Analysis of GO Molecular Function

microtubule motor activity (MF) nucleotide binding (MF) signal transducer activity (MF) protein binding (MF) protein kinase activity (MF) protein kinase binding (MF) insulin receptor substrate binding (MF) Hsp90 protein binding (MF) RNA polymerase II transcription cofactor activity (MF) kinase binding (MF) protein serine/threonine kinase activity (MF) mRNA binding (MF) structural constituent of ribosome (MF) Rho GTPase activator activity (MF) transferase activity, transferring phosphorus-containing groups (MF) ATP binding (MF) protein transporter activity (MF) protein self-association (MF) NADH dehydrogenase (ubiquinone) activity (MF) GTPase activator activity (MF) phosphatidylinositol phospholipase C activity (MF) DNA binding (MF) phospholipase binding (MF) binding (MF) protein heterodimerization activity (MF) transcription coactivator activity (MF) metal ion binding (MF) RNA binding (MF) Rac GTPase activator activity (MF) receptor activity (MF) MAP kinase activity (MF)

HNSC

— Singular Enrichment Analysis of GO Biological Process

positive regulation of cell migration (BP) nerve growth factor receptor signaling pathway (BP) endocrine pancreas development (BP) RNA splicing (BP) axon guidance (BP) chemotaxis (BP) gene expression (BP) viral reproduction (BP) translational elongation (BP) apoptotic process (BP) negative regulation of apoptotic process (BP) positive regulation of cell proliferation (BP) transcription from RNA polymerase II promoter (BP) cellular protein metabolic process (BP) T cell receptor signaling pathway (BP) insulin receptor signaling pathway (BP) transport (BP) viral transcription (BP) cell division (BP) mRNA metabolic process (BP) G-protein coupled receptor signaling pathway (BP) protein phosphorylation (BP) viral infectious cycle (BP) RNA metabolic process (BP) cell adhesion (BP) G2/M transition of mitotic cell cycle (BP) blood coagulation (BP) mitotic cell cycle (BP) translational termination (BP) signal transduction (BP) translation (BP)

— Singular Enrichment Analysis of GO Molecular Function

non-membrane spanning protein tyrosine kinase activity (MF) magnesium ion binding (MF) protein heterodimerization activity (MF) protein kinase binding (MF) heparin binding (MF) glycoprotein binding (MF) ATP binding (MF) guanyl-nucleotide exchange factor activity (MF) transcription coactivator activity (MF) transcription factor binding (MF) protein tyrosine kinase activity (MF) binding (MF) receptor activity (MF) protein binding (MF) protein kinase activity (MF) lyase activity (MF) transferase activity, transferring phosphorus-containing groups (MF) RNA binding (MF) GTPase activity (MF) kinase activity (MF) oxidoreductase activity (MF) collagen binding (MF) SH2 domain binding (MF) structural constituent of ribosome (MF) microtubule binding (MF) activin binding (MF) protein serine/threonine kinase activity (MF) nucleotide binding (MF) protein domain specific binding (MF) electron carrier activity (MF) NADH dehydrogenase (ubiquinone) activity (MF)

KIRC

— Singular Enrichment Analysis of GO Biological Process

anaphase-promoting complex-dependent proteasomal ubiquitin-dependent protein catabolic process (BP) cellular protein metabolic process (BP) cell adhesion (BP) interspecies interaction between organisms (BP) small GTPase mediated signal transduction (BP) actin cytoskeleton organization (BP) S phase of mitotic cell cycle (BP) cell division (BP) negative regulation of cell proliferation (BP) induction of apoptosis by extracellular signals (BP) mitotic cell cycle (BP) blood coagulation (BP) regulation of apoptotic process (BP) nervous system development (BP) platelet activation (BP) RNA metabolic process (BP) signal transduction (BP) cell cycle checkpoint (BP) gene expression (BP) apoptotic process (BP) cell differentiation (BP) G-protein coupled receptor signaling pathway (BP) viral reproduction (BP) positive regulation of transcription, DNA-dependent (BP) protein transport (BP) DNA repair (BP) nerve growth factor receptor signaling pathway (BP) axon guidance (BP) G1/S transition of mitotic cell cycle (BP) mRNA metabolic process (BP) regulation of small GTPase mediated signal transduction (BP)

— Singular Enrichment Analysis of GO Molecular Function

Rac guanyl-nucleotide exchange factor activity (MF) growth factor binding (MF) protein heterodimerization activity (MF) GTPase activity (MF) ubiquitin-protein ligase activity (MF) protein kinase activity (MF) protein binding (MF) nucleotide binding (MF) GTPase activator activity (MF) actin binding (MF) structural constituent of ribosome (MF) DNA-directed RNA polymerase activity (MF) cytochrome-c oxidase activity (MF) protein C-terminus binding (MF) transcription coactivator activity (MF) threonine-type endopeptidase activity (MF) calcium ion binding (MF) Rho guanyl-nucleotide exchange factor activity (MF) ATP-dependent DNA helicase activity (MF) RNA polymerase II transcription cofactor activity (MF) guanyl-nucleotide exchange factor activity (MF) receptor activity (MF) ATP binding (MF) DNA binding (MF) signal transducer activity (MF) protein serine/threonine/tyrosine kinase activity (MF) nucleotidyltransferase activity (MF) ribonucleoprotein complex binding (MF) chemokine activity (MF) cytokine binding (MF) chaperone binding (MF)

KIRP

− Singular Enrichment Analysis of GO Biological Process

apoptotic process (BP) energy reserve metabolic process (BP) gene expression (BP) fibroblast growth factor receptor signaling pathway (BP) protein transport (BP) mitotic cell cycle (BP) transmembrane transport (BP) translation (BP) insulin receptor signaling pathway (BP) nuclear mRNA splicing, via spliceosome (BP) negative regulation of transcription from RNA polymerase II promoter (BP) signal transduction (BP) small GTPase mediated signal transduction (BP) transport (BP) cellular protein metabolic process (BP) cell division (BP) viral reproduction (BP) leukocyte migration (BP) RNA splicing (BP) negative regulation of cell proliferation (BP) respiratory electron transport chain (BP) carbohydrate metabolic process (BP) mRNA metabolic process (BP) nerve growth factor receptor signaling pathway (BP) transcription from RNA polymerase II promoter (BP) axon guidance (BP) blood coagulation (BP) regulation of small GTPase mediated signal transduction (BP) RNA metabolic process (BP) response to drug (BP) platelet activation (BP)

− Singular Enrichment Analysis of GO Molecular Function

phosphatidylinositol-4,5-bisphosphate 3-kinase activity (MF) Rho guanyl-nucleotide exchange factor activity (MF) kinase activity (MF) 1-phosphatidylinositol-3-kinase activity (MF) metal ion binding (MF) phospholipid binding (MF) lipid binding (MF) RNA binding (MF) protein transporter activity (MF) protein binding (MF) nucleotide binding (MF) guanyl-nucleotide exchange factor activity (MF) hydrolase activity (MF) protein complex binding (MF) DNA binding (MF) histone acetyltransferase activity (MF) signal transducer activity (MF) phosphotransferase activity, alcohol group as acceptor (MF) GTPase activity (MF) transcription factor binding (MF) protein C-terminus binding (MF) protein serine/threonine phosphatase activity (MF) GTPase activator activity (MF) protein kinase binding (MF) helicase activity (MF) Rho GTPase activator activity (MF) protein N-terminus binding (MF) structural constituent of ribosome (MF) protein heterodimerization activity (MF) translation initiation factor activity (MF) ATP binding (MF)

LIHC

− Singular Enrichment Analysis of GO Biological Process

immune response (BP) intracellular signal transduction (BP) negative regulation of cell proliferation (BP) nerve growth factor receptor signaling pathway (BP) viral reproduction (BP) translational termination (BP) apoptotic process (BP) regulation of small GTPase mediated signal transduction (BP) viral transcription (BP) protein phosphorylation (BP) RNA splicing (BP) cell division (BP) protein transport (BP) translation (BP) mitotic cell cycle (BP) blood coagulation (BP) axon guidance (BP) transport (BP) translational elongation (BP) RNA metabolic process (BP) gene expression (BP) cellular membrane organization (BP) endocrine pancreas development (BP) positive regulation of cell proliferation (BP) cellular protein metabolic process (BP) energy reserve metabolic process (BP) viral infectious cycle (BP) mRNA metabolic process (BP) small GTPase mediated signal transduction (BP) platelet activation (BP) signal transduction (BP)

− Singular Enrichment Analysis of GO Molecular Function

Rho guanyl-nucleotide exchange factor activity (MF) protein C-terminus binding (MF) GTPase activity (MF) cAMP-dependent protein kinase activity (MF) protein domain specific binding (MF) translation factor activity, nucleic acid binding (MF) translation elongation factor activity (MF) insulin receptor binding (MF) G-protein beta/gamma-subunit complex binding (MF) nucleotide binding (MF) cytochrome-c oxidase activity (MF) protein heterodimerization activity (MF) protein binding (MF) translation initiation factor activity (MF) receptor activity (MF) protein complex binding (MF) insulin-like growth factor receptor binding (MF) receptor tyrosine kinase binding (MF) protein kinase binding (MF) structural constituent of ribosome (MF) guanyl-nucleotide exchange factor activity (MF) motor activity (MF) chemokine activity (MF) RNA binding (MF) ATP binding (MF) GTPase activator activity (MF) interferon-gamma receptor activity (MF) triglyceride lipase activity (MF) microtubule motor activity (MF) receptor binding (MF) NADH dehydrogenase (ubiquinone) activity (MF)

LUAD

− Singular Enrichment Analysis of GO Biological Process

positive regulation of transcription from RNA polymerase II promoter (BP) signal transduction (BP) regulation of small GTPase mediated signal transduction (BP) regulation of transcription, DNA-dependent (BP) innate immune response (BP) mitotic cell cycle (BP) blood coagulation (BP) cell cycle (BP) viral transcription (BP) viral reproduction (BP) carbohydrate metabolic process (BP) RNA metabolic process (BP) cell division (BP) small GTPase mediated signal transduction (BP) inflammatory response (BP) transmembrane transport (BP) cellular protein metabolic process (BP) interspecies interaction between organisms (BP) axon guidance (BP) immune response (BP) endocrine pancreas development (BP) mRNA metabolic process (BP) gene expression (BP) negative regulation of transcription from RNA polymerase II promoter (BP) protein phosphorylation (BP) cell adhesion (BP) translation (BP) transcription from RNA polymerase II promoter (BP) transcription initiation from RNA polymerase II promoter (BP) translational termination (BP) positive regulation of transcription, DNA-dependent (BP)

− Singular Enrichment Analysis of GO Molecular Function

transcription factor binding (MF) protein kinase activity (MF) structural constituent of ribosome (MF) protein binding (MF) structural molecule activity (MF) kinase activity (MF) DNA binding (MF) structural constituent of cytoskeleton (MF) sequence-specific DNA binding transcription factor activity (MF) actin binding (MF) ATP binding (MF) structural constituent of muscle (MF) receptor activity (MF) enzyme binding (MF) protein kinase binding (MF) heparin binding (MF) signal transducer activity (MF) chromatin binding (MF) RNA binding (MF) nucleotide binding (MF) binding (MF) identical protein binding (MF) transcription coactivator activity (MF) protein C-terminus binding (MF) GTPase activator activity (MF) transporter activity (MF) chemokine activity (MF) sugar binding (MF) protein N-terminus binding (MF) protein tyrosine kinase activity (MF) transcription regulatory region DNA binding (MF)

LUSC

− Singular Enrichment Analysis of GO Biological Process

respiratory electron transport chain (BP) RNA splicing (BP) viral reproduction (BP) gene expression (BP) positive regulation of transcription from RNA polymerase II promoter (BP) transcription from RNA polymerase II promoter (BP) nervous system development (BP) cellular protein metabolic process (BP) nuclear mRNA splicing, via spliceosome (BP) mitotic cell cycle (BP) transmembrane transport (BP) positive regulation of transcription, DNA-dependent (BP) protein phosphorylation (BP) cellular component movement (BP) protein transport (BP) cell division (BP) apoptotic process (BP) cell cycle (BP) cell adhesion (BP) interspecies interaction between organisms (BP) response to drug (BP) innate immune response (BP) RNA metabolic process (BP) G2/M transition of mitotic cell cycle (BP) transport (BP) cellular membrane organization (BP) blood coagulation (BP) small GTPase mediated signal transduction (BP) signal transduction (BP) axon guidance (BP) negative regulation of cell proliferation (BP)

− Singular Enrichment Analysis of GO Molecular Function

motor activity (MF) RNA polymerase II transcription cofactor activity (MF) transcription factor binding (MF) binding (MF) protein binding (MF) transmembrane signaling receptor activity (MF) protein serine/threonine kinase activity (MF) histone binding (MF) actin binding (MF) NADH dehydrogenase (ubiquinone) activity (MF) guanyl-nucleotide exchange factor activity (MF) transcription regulatory region DNA binding (MF) protein transporter activity (MF) transcription coactivator activity (MF) cytokine receptor activity (MF) GTP binding (MF) RNA binding (MF) protein kinase binding (MF) signal transducer activity (MF) ubiquitin protein ligase binding (MF) structural constituent of ribosome (MF) beta-catenin binding (MF) chromatin binding (MF) receptor activity (MF) protein kinase activity (MF) nucleotide binding (MF) extracellular matrix structural constituent (MF) ATP binding (MF) DNA binding (MF) GTPase activity (MF) transferase activity, transferring phosphorus-containing groups (MF)

PRAD

− Singular Enrichment Analysis of GO Biological Process

gene expression (BP) G1/S transition of mitotic cell cycle (BP) axon guidance (BP) regulation of small GTPase mediated signal transduction (BP) cell cycle checkpoint (BP) apoptotic process (BP) blood coagulation (BP) RNA splicing (BP) viral reproduction (BP) platelet activation (BP) cellular protein metabolic process (BP) nerve growth factor receptor signaling pathway (BP) transport (BP) protein phosphorylation (BP) negative regulation of transcription, DNA-dependent (BP) mitotic cell cycle (BP) positive regulation of transcription from RNA polymerase II promoter (BP) nuclear mRNA splicing, via spliceosome (BP) insulin receptor signaling pathway (BP) heart development (BP) multicellular organismal development (BP) cell division (BP) cell differentiation (BP) cell cycle (BP) mRNA metabolic process (BP) S phase of mitotic cell cycle (BP) DNA repair (BP) RNA metabolic process (BP) signal transduction (BP) small GTPase mediated signal transduction (BP) transcription from RNA polymerase II promoter (BP)

− Singular Enrichment Analysis of GO Molecular Function

ATP binding (MF) ephrin receptor binding (MF) protein binding (MF) protein serine/threonine kinase activity (MF) transferase activity (MF) protein kinase activity (MF) protein homodimerization activity (MF) chromatin binding (MF) hydrolase activity (MF) ligase activity (MF) heme binding (MF) calcium ion binding (MF) non-membrane spanning protein tyrosine kinase activity (MF) PDZ domain binding (MF) guanyl-nucleotide exchange factor activity (MF) kinase activity (MF) nucleotide binding (MF) protein kinase C binding (MF) SH3/SH2 adaptor activity (MF) integrin binding (MF) structural constituent of ribosome (MF) enzyme binding (MF) protein tyrosine kinase activity (MF) GTPase activity (MF) protein domain specific binding (MF) RNA binding (MF) Rho guanyl-nucleotide exchange factor activity (MF) receptor binding (MF) signal transducer activity (MF) identical protein binding (MF) GTP binding (MF)

PVT1

BLCA

− Singular Enrichment Analysis of GO Biological Process

intracellular signal transduction (BP) regulation of small GTPase mediated signal transduction (BP) mRNA metabolic process (BP) transport (BP) viral reproduction (BP) positive regulation of transcription from RNA polymerase II promoter (BP) multicellular organismal development (BP) Toll signaling pathway (BP) gene expression (BP) toll-like receptor 4 signaling pathway (BP) nuclear mRNA splicing, via spliceosome (BP) toll-like receptor 1 signaling pathway (BP) RNA metabolic process (BP) cell division (BP) mitotic cell cycle (BP) signal transduction (BP) small GTPase mediated signal transduction (BP) G-protein coupled receptor signaling pathway (BP) innate immune response (BP) nerve growth factor receptor signaling pathway (BP) RNA splicing (BP) negative regulation of cell proliferation (BP) cell cycle (BP) apoptotic process (BP) protein phosphorylation (BP) blood coagulation (BP) transcription from RNA polymerase II promoter (BP) toll-like receptor signaling pathway (BP) axon guidance (BP) toll-like receptor 2 signaling pathway (BP) MyD88-dependent toll-like receptor signaling pathway (BP)

− Singular Enrichment Analysis of GO Molecular Function

microtubule motor activity (MF) nucleotide binding (MF) signal transducer activity (MF) protein binding (MF) protein kinase activity (MF) protein kinase binding (MF) insulin receptor substrate binding (MF) Hsp90 protein binding (MF) RNA polymerase II transcription cofactor activity (MF) kinase binding (MF) protein serine/threonine kinase activity (MF) mRNA binding (MF) structural constituent of ribosome (MF) Rho GTPase activator activity (MF) transferase activity, transferring phosphorus-containing groups (MF) ATP binding (MF) protein transporter activity (MF) protein self-association (MF) NADH dehydrogenase (ubiquinone) activity (MF) GTPase activator activity (MF) phosphatidylinositol phospholipase C activity (MF) DNA binding (MF) phospholipase binding (MF) binding (MF) protein heterodimerization activity (MF) transcription coactivator activity (MF) metal ion binding (MF) RNA binding (MF) Rac GTPase activator activity (MF) receptor activity (MF) MAP kinase activity (MF)

<u>HNSC</u>

— Singular Enrichment Analysis of GO Biological Process

cell differentiation (BP) mitotic cell cycle (BP) nerve growth factor receptor signaling pathway (BP) positive regulation of cell proliferation (BP) cellular protein metabolic process (BP) interspecies interaction between organisms (BP) regulation of transcription, DNA-dependent (BP) respiratory electron transport chain (BP) mRNA metabolic process (BP) blood coagulation (BP) signal transduction (BP) transport (BP) RNA splicing (BP) transcription from RNA polymerase II promoter (BP) G2/M transition of mitotic cell cycle (BP) regulation of small GTPase mediated signal transduction (BP) negative regulation of transcription from RNA polymerase II promoter (BP) axon guidance (BP) apoptotic process (BP) intracellular signal transduction (BP) positive regulation of transcription from RNA polymerase II promoter (BP) small GTPase mediated signal transduction (BP) insulin receptor signaling pathway (BP) response to hypoxia (BP) RNA metabolic process (BP) cell division (BP) gene expression (BP) nuclear mRNA splicing, via spliceosome (BP) viral reproduction (BP) multicellular organismal development (BP) negative regulation of cell proliferation (BP)

— Singular Enrichment Analysis of GO Molecular Function

double-stranded DNA binding (MF) chromatin binding (MF) GTPase activity (MF) transferase activity, transferring phosphorus-containing groups (MF) extracellular matrix structural constituent (MF) binding (MF) RNA binding (MF) phosphatidylinositol binding (MF) protein binding (MF) guanyl-nucleotide exchange factor activity (MF) protein kinase activity (MF) structural molecule activity (MF) transcription regulatory region DNA binding (MF) transcription factor binding (MF) ATPase activity (MF) activin binding (MF) structural constituent of ribosome (MF) NADH dehydrogenase (ubiquinone) activity (MF) calcium ion binding (MF) phospholipid binding (MF) protein complex binding (MF) hydrolase activity (MF) sequence-specific DNA binding (MF) sequence-specific DNA binding transcription factor activity (MF) microtubule motor activity (MF) protein kinase C binding (MF) protein tyrosine kinase activity (MF) GTP binding (MF) nucleotide binding (MF) DNA binding (MF) ATP binding (MF)

<u>KIRC</u>

— Singular Enrichment Analysis of GO Biological Process

innate immune response (BP) cellular protein metabolic process (BP) positive regulation of cell proliferation (BP) RNA splicing (BP) RNA metabolic process (BP) positive regulation of transcription from RNA polymerase II promoter (BP) S phase of mitotic cell cycle (BP) regulation of transcription, DNA-dependent (BP) signal transduction (BP) mitotic cell cycle (BP) positive regulation of transcription, DNA-dependent (BP) blood coagulation (BP) cell cycle (BP) platelet activation (BP) nuclear mRNA splicing, via spliceosome (BP) mRNA metabolic process (BP) regulation of apoptotic process (BP) apoptotic process (BP) respiratory electron transport chain (BP) axon guidance (BP) negative regulation of transcription from RNA polymerase II promoter (BP) transcription from RNA polymerase II promoter (BP) interspecies interaction between organisms (BP) gene expression (BP) G1/S transition of mitotic cell cycle (BP) protein phosphorylation (BP) DNA repair (BP) translation (BP) transport (BP) viral reproduction (BP) nerve growth factor receptor signaling pathway (BP)

— Singular Enrichment Analysis of GO Molecular Function

DNA binding (MF) growth factor binding (MF) GTPase activity (MF) protein tyrosine kinase activity (MF) nucleotide binding (MF) ATP binding (MF) protein C-terminus binding (MF) enzyme binding (MF) protein heterodimerization activity (MF) protein serine/threonine kinase activity (MF) protein domain specific binding (MF) protein kinase activity (MF) fibroblast growth factor binding (MF) receptor binding (MF) hydrolase activity (MF) heparin binding (MF) transcription coactivator activity (MF) ATPase activity (MF) protein binding (MF) NADH dehydrogenase (ubiquinone) activity (MF) nucleotidyltransferase activity (MF) structural constituent of ribosome (MF) phosphatidylinositol binding (MF) kinase activity (MF) nucleoside-triphosphatase activity (MF) GTP binding (MF) chromatin binding (MF) RNA binding (MF) structural constituent of cytoskeleton (MF) protein homodimerization activity (MF) ion channel activity (MF)

KIRP

– Singular Enrichment Analysis of GO Biological Process

nuclear mRNA splicing, via spliceosome (BP) translation (BP) interspecies interaction between organisms (BP) respiratory electron transport chain (BP) transmembrane transport (BP) axon guidance (BP) positive regulation of transcription from RNA polymerase II promoter (BP) innate immune response (BP) mRNA metabolic process (BP) synaptic transmission (BP) negative regulation of apoptotic process (BP) transcription from RNA polymerase II promoter (BP) transport (BP) viral infectious cycle (BP) gene expression (BP) ion transport (BP) blood coagulation (BP) viral reproduction (BP) inflammatory response (BP) RNA splicing (BP) carbohydrate metabolic process (BP) RNA metabolic process (BP) regulation of transcription, DNA-dependent (BP) regulation of small GTPase mediated signal transduction (BP) small GTPase mediated signal transduction (BP) signal transduction (BP) mitotic cell cycle (BP) cellular nitrogen compound metabolic process (BP) positive regulation of transcription, DNA-dependent (BP) cellular protein metabolic process (BP) platelet activation (BP)

– Singular Enrichment Analysis of GO Molecular Function

RNA polymerase II transcription cofactor activity (MF) structural constituent of ribosome (MF) transcription coactivator activity (MF) protein domain specific binding (MF) RNA binding (MF) GTPase activator activity (MF) protein kinase activity (MF) ATP binding (MF) hydrolase activity, acting on acid anhydrides, catalyzing transmembrane movement of substances (MF) 4 iron, 4 sulfur cluster binding (MF) histone acetyltransferase activity (MF) phosphatidylinositol phospholipase C activity (MF) cysteine-type endopeptidase inhibitor activity involved in apoptotic process (MF) magnesium ion binding (MF) hydrolase activity (MF) oxidoreductase activity (MF) inward rectifier potassium channel activity (MF) protein binding (MF) proton-transporting ATPase activity, rotational mechanism (MF) NADH dehydrogenase (ubiquinone) activity (MF) NAD binding (MF) translation initiation factor activity (MF) nucleotide binding (MF) metal ion binding (MF) protein heterodimerization activity (MF) hydrogen ion transporting ATP synthase activity, rotational mechanism (MF) SMAD binding (MF) protein serine/threonine kinase activity (MF) ligand-dependent nuclear receptor transcription coactivator activity (MF) Rho GTPase activator activity (MF) protein homodimerization activity (MF)

LIHC

– Singular Enrichment Analysis of GO Biological Process

RNA splicing (BP) viral reproduction (BP) cell division (BP) cell cycle (BP) positive regulation of transcription, DNA-dependent (BP) blood coagulation (BP) DNA replication (BP) viral transcription (BP) nerve growth factor receptor signaling pathway (BP) cellular membrane organization (BP) positive regulation of transcription from RNA polymerase II promoter (BP) regulation of transcription, DNA-dependent (BP) mRNA metabolic process (BP) protein phosphorylation (BP) transcription from RNA polymerase II promoter (BP) RNA metabolic process (BP) protein transport (BP) viral infectious cycle (BP) translation (BP) negative regulation of transcription, DNA-dependent (BP) apoptotic process (BP) nuclear mRNA splicing, via spliceosome (BP) cell cycle checkpoint (BP) axon guidance (BP) DNA repair (BP) mitotic cell cycle (BP) interspecies interaction between organisms (BP) gene expression (BP) mitosis (BP) G2/M transition of mitotic cell cycle (BP) cellular protein metabolic process (BP)

– Singular Enrichment Analysis of GO Molecular Function

kinase activity (MF) ubiquitin-protein ligase activity (MF) transcription cofactor activity (MF) RNA polymerase II transcription cofactor activity (MF) helicase activity (MF) protein binding (MF) transcription factor binding (MF) transcription coactivator activity (MF) protein N-terminus binding (MF) GTPase activity (MF) protein kinase binding (MF) RNA binding (MF) double-stranded DNA binding (MF) DNA binding (MF) ATP binding (MF) structural constituent of ribosome (MF) ligand-dependent nuclear receptor transcription coactivator activity (MF) transferase activity, transferring phosphorus-containing groups (MF) SH3 domain binding (MF) hydrolase activity (MF) protein serine/threonine kinase activity (MF) translation initiation factor activity (MF) GTPase activator activity (MF) transcription regulatory region DNA binding (MF) GTP binding (MF) identical protein binding (MF) protein kinase activity (MF) nucleotide binding (MF) chromatin binding (MF) binding (MF) protein C-terminus binding (MF)

LUAD

− Singular Enrichment Analysis of GO Biological Process

regulation of ubiquitin-protein ligase activity involved in mitotic cell cycle (BP) positive regulation of transcription, DNA-dependent (BP) G-protein coupled receptor signaling pathway (BP) small GTPase mediated signal transduction (BP) positive regulation of transcription from RNA polymerase II promoter (BP) intracellular signal transduction (BP) platelet activation (BP) mitotic cell cycle (BP) nerve growth factor receptor signaling pathway (BP) cellular protein metabolic process (BP) cell division (BP) RNA metabolic process (BP) S phase of mitotic cell cycle (BP) negative regulation of ubiquitin-protein ligase activity involved in mitotic cell cycle (BP) mRNA metabolic process (BP) transport (BP) translation (BP) M phase of mitotic cell cycle (BP) anaphase-promoting complex-dependent proteasomal ubiquitin-dependent protein catabolic process (BP) G1/S transition of mitotic cell cycle (BP) blood coagulation (BP) signal transduction (BP) cell cycle checkpoint (BP) protein phosphorylation (BP) positive regulation of ubiquitin-protein ligase activity involved in mitotic cell cycle (BP) viral reproduction (BP) apoptotic process (BP) regulation of small GTPase mediated signal transduction (BP) response to hypoxia (BP) mitotic prometaphase (BP) gene expression (BP)

− Singular Enrichment Analysis of GO Molecular Function

translation initiation factor activity (MF) hydrolase activity (MF) lipid binding (MF) nucleotide binding (MF) Rho guanyl-nucleotide exchange factor activity (MF) chemokine activity (MF) receptor activity (MF) protein kinase activity (MF) structural constituent of ribosome (MF) protein kinase binding (MF) transmembrane receptor protein serine/threonine kinase activity (MF) protein serine/threonine kinase activity (MF) phosphoric ester hydrolase activity (MF) calmodulin binding (MF) ATP binding (MF) transferase activity, transferring phosphorus-containing groups (MF) SMAD binding (MF) G-protein coupled receptor activity (MF) phosphatidylinositol binding (MF) protein binding (MF) nucleotidyltransferase activity (MF) RNA binding (MF) ubiquinol-cytochrome-c reductase activity (MF) heme binding (MF) signal transducer activity (MF) protein heterodimerization activity (MF) cytokine activity (MF) ubiquitin-protein ligase activity (MF) guanyl-nucleotide exchange factor activity (MF) lipopolysaccharide binding (MF) acid-amino acid ligase activity (MF)

LUSC

− Singular Enrichment Analysis of GO Biological Process

transport (BP) translational termination (BP) G2/M transition of mitotic cell cycle (BP) transmembrane transport (BP) mitotic prometaphase (BP) signal transduction (BP) M phase of mitotic cell cycle (BP) blood coagulation (BP) nucleotide-excision repair (BP) RNA metabolic process (BP) viral reproduction (BP) apoptotic process (BP) positive regulation of transcription from RNA polymerase II promoter (BP) DNA repair (BP) S phase of mitotic cell cycle (BP) protein phosphorylation (BP) interspecies interaction between organisms (BP) axon guidance (BP) nuclear mRNA splicing, via spliceosome (BP) mRNA metabolic process (BP) cellular protein metabolic process (BP) cell division (BP) translation (BP) transcription from RNA polymerase II promoter (BP) G1/S transition of mitotic cell cycle (BP) negative regulation of transcription from RNA polymerase II promoter (BP) regulation of transcription, DNA-dependent (BP) gene expression (BP) RNA splicing (BP) mitotic cell cycle (BP) translational elongation (BP)

− Singular Enrichment Analysis of GO Molecular Function

catalytic activity (MF) metal ion binding (MF) heparin binding (MF) proton-transporting ATPase activity, rotational mechanism (MF) protein kinase activity (MF) signal transducer activity (MF) structural constituent of ribosome (MF) DNA-directed RNA polymerase activity (MF) zinc ion binding (MF) extracellular matrix structural constituent (MF) enzyme binding (MF) unfolded protein binding (MF) ATPase activity (MF) RNA binding (MF) chromatin binding (MF) protein C-terminus binding (MF) hydrolase activity (MF) GTPase activator activity (MF) protein kinase binding (MF) protein homodimerization activity (MF) structural molecule activity (MF) protein binding (MF) nucleotide binding (MF) ligase activity (MF) integrin binding (MF) sequence-specific DNA binding transcription factor activity (MF) DNA binding (MF) ubiquitin-protein ligase activity (MF) protein domain specific binding (MF) ATP binding (MF) receptor binding (MF)

PRAD

– Singular Enrichment Analysis of GO Biological Process

regulation of transcription, DNA-dependent (BP) axon guidance (BP) protein transport (BP) transmembrane transport (BP) innate immune response (BP) positive regulation of transcription, DNA-dependent (BP) positive regulation of transcription from RNA polymerase II promoter (BP) interspecies interaction between organisms (BP) RNA metabolic process (BP) protein phosphorylation (BP) nerve growth factor receptor signaling pathway (BP) cellular protein metabolic process (BP) translation (BP) carbohydrate metabolic process (BP) platelet activation (BP) blood coagulation (BP) intracellular signal transduction (BP) viral transcription (BP) translational elongation (BP) regulation of small GTPase mediated signal transduction (BP) gene expression (BP) viral infectious cycle (BP) negative regulation of cell proliferation (BP) mRNA metabolic process (BP) mitotic cell cycle (BP) endocrine pancreas development (BP) Toll signaling pathway (BP) viral reproduction (BP) apoptotic process (BP) signal transduction (BP) small GTPase mediated signal transduction (BP)

– Singular Enrichment Analysis of GO Molecular Function

G-protein beta/gamma-subunit complex binding (MF) ligase activity (MF) RNA binding (MF) ubiquitin-protein ligase activity (MF) ATPase activity (MF) transferase activity (MF) protein binding (MF) protein kinase activity (MF) protein serine/threonine kinase activity (MF) GTP binding (MF) identical protein binding (MF) transcription coactivator activity (MF) transferase activity, transferring phosphorus-containing groups (MF) structural constituent of ribosome (MF) chromatin binding (MF) hydrogen ion transporting ATP synthase activity, rotational mechanism (MF) proton-transporting ATPase activity, rotational mechanism (MF) flavin adenine dinucleotide binding (MF) histone acetyltransferase activity (MF) protein N-terminus binding (MF) ATP binding (MF) GTPase activator activity (MF) transferase activity, transferring acyl groups (MF) nucleotide binding (MF) protein heterodimerization activity (MF) guanyl-nucleotide exchange factor activity (MF) RNA polymerase II transcription cofactor activity (MF) metal ion binding (MF) GTPase activity (MF) structural molecule activity (MF) hydrolase activity (MF)

SNHG11

BLCA

– Singular Enrichment Analysis of GO Biological Process

intracellular signal transduction (BP) mRNA metabolic process (BP) regulation of apoptotic process (BP) mitotic cell cycle (BP) G1/S transition of mitotic cell cycle (BP) signal transduction (BP) transcription from RNA polymerase II promoter (BP) gene expression (BP) RNA metabolic process (BP) cell proliferation (BP) M/G1 transition of mitotic cell cycle (BP) insulin receptor signaling pathway (BP) RNA splicing (BP) transcription elongation from RNA polymerase II promoter (BP) protein phosphorylation (BP) translation (BP) immune response (BP) cellular protein metabolic process (BP) S phase of mitotic cell cycle (BP) nuclear mRNA splicing, via spliceosome (BP) cell cycle (BP) negative regulation of transcription, DNA-dependent (BP) negative regulation of cell proliferation (BP) viral infectious cycle (BP) cell cycle checkpoint (BP) viral reproduction (BP) cell adhesion (BP) apoptotic process (BP) interspecies interaction between organisms (BP) axon guidance (BP) blood coagulation (BP)

– Singular Enrichment Analysis of GO Molecular Function

nucleoside-triphosphatase activity (MF) GTPase activity (MF) glucuronosyl-N-acetylgalactosaminyl-proteoglycan 4-beta-N-acetylgalactosaminyltransferase activity (MF) receptor activity (MF) transferase activity (MF) GTP binding (MF) metal ion binding (MF) 3',5'-cyclic-AMP phosphodiesterase activity (MF) protein kinase activity (MF) extracellular matrix structural constituent (MF) damaged DNA binding (MF) binding (MF) phosphoric diester hydrolase activity (MF) calcium ion binding (MF) kinase activity (MF) hydrolase activity (MF) protein serine/threonine kinase activity (MF) transmembrane signaling receptor activity (MF) protein kinase binding (MF) DNA binding (MF) structural constituent of ribosome (MF) eukaryotic cell surface binding (MF) transferase activity, transferring hexosyl groups (MF) collagen binding (MF) transferase activity, transferring phosphorus-containing groups (MF) protein binding (MF) ATP binding (MF) catalytic activity (MF) nucleotide binding (MF) NADH dehydrogenase (ubiquinone) activity (MF) RNA binding (MF)

HNSC

— Singular Enrichment Analysis of GO Biological Process

cellular protein metabolic process (BP) cell cycle (BP) blood coagulation (BP) viral transcription (BP) inflammatory response (BP) induction of apoptosis by extracellular signals (BP) RNA metabolic process (BP) nervous system development (BP) intracellular signal transduction (BP) mitotic cell cycle (BP) axon guidance (BP) cell adhesion (BP) regulation of apoptotic process (BP) nerve growth factor receptor signaling pathway (BP) small GTPase mediated signal transduction (BP) translational termination (BP) interspecies interaction between organisms (BP) transport (BP) endocrine pancreas development (BP) protein phosphorylation (BP) positive regulation of transcription from RNA polymerase II promoter (BP) signal transduction (BP) gene expression (BP) immune response (BP) translation (BP) mRNA metabolic process (BP) regulation of small GTPase mediated signal transduction (BP) viral reproduction (BP) protein transport (BP) apoptotic process (BP) platelet activation (BP)

— Singular Enrichment Analysis of GO Molecular Function

transcription corepressor activity (MF) kinase activity (MF) protein N-terminus binding (MF) receptor activity (MF) histone acetyltransferase activity (MF) transferase activity (MF) Rho guanyl-nucleotide exchange factor activity (MF) chromatin binding (MF) protein kinase C binding (MF) GTP binding (MF) protein kinase activity (MF) insulin receptor binding (MF) RNA binding (MF) protein domain specific binding (MF) ubiquitin protein ligase binding (MF) transferase activity, transferring phosphorus-containing groups (MF) DNA binding (MF) histone deacetylase binding (MF) structural constituent of ribosome (MF) microtubule binding (MF) guanyl-nucleotide exchange factor activity (MF) GTPase activator activity (MF) integrin binding (MF) nucleotide binding (MF) GTPase activity (MF) transmembrane signaling receptor activity (MF) ATP binding (MF) protein binding (MF) flavin adenine dinucleotide binding (MF) protein phosphatase binding (MF) transcription coactivator activity (MF)

KICH

— Singular Enrichment Analysis of GO Biological Process

small GTPase mediated signal transduction (BP) axon guidance (BP) viral reproduction (BP) nervous system development (BP) regulation of small GTPase mediated signal transduction (BP) negative regulation of transcription, DNA-dependent (BP) nuclear mRNA splicing, via spliceosome (BP) negative regulation of transcription from RNA polymerase II promoter (BP) positive regulation of transcription from RNA polymerase II promoter (BP) mRNA metabolic process (BP) mitotic cell cycle (BP) RNA metabolic process (BP) transport (BP) response to virus (BP) embryo development (BP) gene expression (BP) blood coagulation (BP) viral infectious cycle (BP) interspecies interaction between organisms (BP) transcription from RNA polymerase II promoter (BP) protein phosphorylation (BP) cellular protein metabolic process (BP) innate immune response (BP) response to drug (BP) negative regulation of cell proliferation (BP) intracellular signal transduction (BP) apoptotic process (BP) signal transduction (BP) RNA splicing (BP) positive regulation of transcription, DNA-dependent (BP) G-protein coupled receptor signaling pathway (BP)

— Singular Enrichment Analysis of GO Molecular Function

receptor binding (MF) Rho GTPase activator activity (MF) transcription regulatory region DNA binding (MF) DNA binding (MF) transferase activity, transferring acyl groups (MF) receptor activity (MF) double-stranded DNA binding (MF) protein homodimerization activity (MF) transcription factor binding (MF) protein kinase binding (MF) protein binding (MF) protein kinase activity (MF) signal transducer activity (MF) transferase activity (MF) ATP binding (MF) enzyme binding (MF) identical protein binding (MF) structural constituent of ribosome (MF) G-protein coupled receptor activity (MF) protein complex binding (MF) transcription coactivator activity (MF) ubiquitin protein ligase binding (MF) DNA-directed RNA polymerase activity (MF) kinase activity (MF) protein heterodimerization activity (MF) transferase activity, transferring phosphorus-containing groups (MF) GTPase activator activity (MF) sequence-specific DNA binding transcription factor activity (MF) RNA binding (MF) beta-catenin binding (MF) nucleotide binding (MF)

KIRC

– Singular Enrichment Analysis of GO Biological Process

regulation of transcription, DNA-dependent (BP) positive regulation of transcription from RNA polymerase II promoter (BP) apoptotic process (BP) innate immune response (BP) protein phosphorylation (BP) blood coagulation (BP) nuclear mRNA splicing, via spliceosome (BP) DNA repair (BP) mitotic cell cycle (BP) toll-like receptor signaling pathway (BP) cell division (BP) intracellular signal transduction (BP) transport (BP) gene expression (BP) signal transduction (BP) cell cycle (BP) regulation of transcription from RNA polymerase II promoter (BP) transcription from RNA polymerase II promoter (BP) protein transport (BP) translation (BP) regulation of small GTPase mediated signal transduction (BP) small GTPase mediated signal transduction (BP) axon guidance (BP) RNA splicing (BP) viral reproduction (BP) synaptic transmission (BP) mRNA metabolic process (BP) cellular protein metabolic process (BP) nerve growth factor receptor signaling pathway (BP) negative regulation of cell proliferation (BP) RNA metabolic process (BP)

– Singular Enrichment Analysis of GO Molecular Function

DNA-directed RNA polymerase activity (MF) protein serine/threonine kinase activity (MF) guanyl-nucleotide exchange factor activity (MF) translation initiation factor activity (MF) RNA polymerase II core promoter proximal region sequence-specific DNA binding (MF) transmembrane signaling receptor activity (MF) netrin receptor activity (MF) DNA binding (MF) NADH dehydrogenase (ubiquinone) activity (MF) protein binding (MF) damaged DNA binding (MF) protein kinase activity (MF) structural constituent of ribosome (MF) receptor activity (MF) transferase activity, transferring phosphorus-containing groups (MF) protein kinase binding (MF) protein domain specific binding (MF) ATP binding (MF) RNA binding (MF) transcription coactivator activity (MF) protein tyrosine kinase activity (MF) protein phosphatase binding (MF) translation elongation factor activity (MF) protein homodimerization activity (MF) RNA polymerase II transcription cofactor activity (MF) microtubule binding (MF) protein binding transcription factor activity (MF) nucleotide binding (MF) protein C-terminus binding (MF) signal transducer activity (MF) magnesium ion binding (MF)

KIRP

– Singular Enrichment Analysis of GO Biological Process

blood coagulation (BP) G1/S transition of mitotic cell cycle (BP) gene expression (BP) regulation of ubiquitin-protein ligase activity involved in mitotic cell cycle (BP) RNA metabolic process (BP) S phase of mitotic cell cycle (BP) axon guidance (BP) intracellular signal transduction (BP) nucleotide-excision repair (BP) protein phosphorylation (BP) negative regulation of ubiquitin-protein ligase activity involved in mitotic cell cycle (BP) cell cycle checkpoint (BP) DNA repair (BP) viral reproduction (BP) positive regulation of transcription, DNA-dependent (BP) small GTPase mediated signal transduction (BP) positive regulation of ubiquitin-protein ligase activity involved in mitotic cell cycle (BP) protein polyubiquitination (BP) regulation of transcription, DNA-dependent (BP) positive regulation of cell proliferation (BP) nerve growth factor receptor signaling pathway (BP) interspecies interaction between organisms (BP) mitotic cell cycle (BP) anaphase-promoting complex-dependent proteasomal ubiquitin-dependent protein catabolic process (BP) mRNA metabolic process (BP) signal transduction (BP) apoptotic process (BP) transcription from RNA polymerase II promoter (BP) M/G1 transition of mitotic cell cycle (BP) cell division (BP) cellular nitrogen compound metabolic process (BP)

– Singular Enrichment Analysis of GO Molecular Function

Rho GTPase activator activity (MF) binding (MF) transferase activity, transferring phosphorus-containing groups (MF) beta-catenin binding (MF) translation initiation factor activity (MF) nucleotide binding (MF) ATP binding (MF) protein domain specific binding (MF) NADH dehydrogenase (ubiquinone) activity (MF) protein C-terminus binding (MF) growth factor activity (MF) ubiquitin-protein ligase activity (MF) protein phosphatase binding (MF) GTPase activator activity (MF) protein complex binding (MF) ubiquitin protein ligase binding (MF) ATPase activity (MF) damaged DNA binding (MF) protein tyrosine kinase activity (MF) metal ion binding (MF) protein transporter activity (MF) DNA binding (MF) nucleoside-triphosphatase activity (MF) threonine-type endopeptidase activity (MF) protein kinase binding (MF) protein serine/threonine kinase activity (MF) SH2 domain binding (MF) kinase activity (MF) protein binding (MF) protein kinase activity (MF) peptidase activity (MF)

LUAD

– Singular Enrichment Analysis of GO Biological Process

cellular protein metabolic process (BP) gene expression (BP) platelet activation (BP) interspecies interaction between organisms (BP) nerve growth factor receptor signaling pathway (BP) innate immune response (BP) intracellular signal transduction (BP) cell adhesion (BP) protein phosphorylation (BP) RNA splicing (BP) small GTPase mediated signal transduction (BP) insulin receptor signaling pathway (BP) RNA metabolic process (BP) blood coagulation (BP) positive regulation of transcription from RNA polymerase II promoter (BP) cell division (BP) signal transduction (BP) positive regulation of cell proliferation (BP) apoptotic process (BP) mitotic cell cycle (BP) nuclear mRNA splicing, via spliceosome (BP) positive regulation of transcription, DNA-dependent (BP) transmembrane transport (BP) DNA repair (BP) cell cycle (BP) multicellular organismal development (BP) response to drug (BP) regulation of small GTPase mediated signal transduction (BP) fibroblast growth factor receptor signaling pathway (BP) transcription from RNA polymerase II promoter (BP) viral reproduction (BP)

– Singular Enrichment Analysis of GO Molecular Function

transcription regulatory region DNA binding (MF) magnesium ion binding (MF) RNA binding (MF) growth factor activity (MF) guanyl-nucleotide exchange factor activity (MF) lipid binding (MF) serine-type endopeptidase inhibitor activity (MF) structural molecule activity (MF) transferase activity, transferring glycosyl groups (MF) nucleotide binding (MF) transferase activity (MF) metal ion binding (MF) chromatin binding (MF) structural constituent of ribosome (MF) receptor activity (MF) ATP binding (MF) receptor binding (MF) binding (MF) GTPase activity (MF) GTPase activator activity (MF) ubiquitin-protein ligase activity (MF) enzyme binding (MF) protein heterodimerization activity (MF) GTP binding (MF) protein tyrosine kinase activity (MF) identical protein binding (MF) protein kinase activity (MF) heparin binding (MF) protein binding (MF) signal transducer activity (MF) receptor signaling protein activity (MF)

LUSC

– Singular Enrichment Analysis of GO Biological Process

mRNA metabolic process (BP) regulation of transcription, DNA-dependent (BP) cell division (BP) translational termination (BP) viral infectious cycle (BP) gene expression (BP) G-protein coupled receptor signaling pathway (BP) nerve growth factor receptor signaling pathway (BP) RNA splicing (BP) cellular protein metabolic process (BP) transmembrane transport (BP) protein transport (BP) interspecies interaction between organisms (BP) transcription from RNA polymerase II promoter (BP) small GTPase mediated signal transduction (BP) RNA metabolic process (BP) signal transduction (BP) regulation of small GTPase mediated signal transduction (BP) blood coagulation (BP) viral transcription (BP) apoptotic process (BP) translational elongation (BP) viral reproduction (BP) mitotic cell cycle (BP) anti-apoptosis (BP) response to hypoxia (BP) transport (BP) translation (BP) insulin receptor signaling pathway (BP) cell cycle (BP) endocrine pancreas development (BP)

– Singular Enrichment Analysis of GO Molecular Function

GTPase activator activity (MF) structural constituent of ribosome (MF) ATPase activity (MF) protein heterodimerization activity (MF) ubiquitin-protein ligase activity (MF) ATP binding (MF) protein serine/threonine kinase activity (MF) protein binding (MF) receptor activity (MF) protein kinase activity (MF) hydrolase activity (MF) GTP binding (MF) ubiquitin protein ligase binding (MF) lipid binding (MF) transcription factor binding (MF) protein kinase binding (MF) enzyme binding (MF) translation initiation factor activity (MF) nucleotide binding (MF) RNA binding (MF) Rho guanyl-nucleotide exchange factor activity (MF) transcription coactivator activity (MF) GTPase activity (MF) signal transducer activity (MF) G-protein coupled receptor activity (MF) ligase activity (MF) protein homodimerization activity (MF) magnesium ion binding (MF) guanyl-nucleotide exchange factor activity (MF) identical protein binding (MF) growth factor activity (MF)

PRAD

− Singular Enrichment Analysis of GO Biological Process

transcription from RNA polymerase II promoter (BP) mitotic cell cycle (BP) transcription-coupled nucleotide-excision repair (BP) apoptotic process (BP) DNA repair (BP) protein phosphorylation (BP) positive regulation of transcription from RNA polymerase II promoter (BP) cellular protein metabolic process (BP) induction of apoptosis by extracellular signals (BP) nuclear mRNA splicing, via spliceosome (BP) S phase of mitotic cell cycle (BP) cell division (BP) blood coagulation (BP) RNA metabolic process (BP) insulin receptor signaling pathway (BP) mRNA metabolic process (BP) small GTPase mediated signal transduction (BP) RNA splicing (BP) transmembrane transport (BP) carbohydrate metabolic process (BP) cell proliferation (BP) transport (BP) nucleotide-excision repair (BP) nerve growth factor receptor signaling pathway (BP) post-translational protein modification (BP) viral reproduction (BP) signal transduction (BP) gene expression (BP) protein polyubiquitination (BP) cell cycle checkpoint (BP) G1/S transition of mitotic cell cycle (BP)

− Singular Enrichment Analysis of GO Molecular Function

microtubule motor activity (MF) chromatin binding (MF) protein kinase activity (MF) transferase activity (MF) protein serine/threonine kinase activity (MF) GTP binding (MF) RNA binding (MF) protein binding (MF) PDZ domain binding (MF) signal transducer activity (MF) DNA-directed DNA polymerase activity (MF) protein homodimerization activity (MF) ATP binding (MF) phosphatidylinositol phospholipase C activity (MF) electron carrier activity (MF) kinase activity (MF) nucleotide binding (MF) metal ion binding (MF) GTPase activator activity (MF) peptidase activity (MF) NADH dehydrogenase (ubiquinone) activity (MF) peptide binding (MF) hydrolase activity, acting on glycosyl bonds (MF) hydrolase activity (MF) endopeptidase activity (MF) protein N-terminus binding (MF) threonine-type endopeptidase activity (MF) magnesium ion binding (MF) DNA binding (MF) protein kinase binding (MF) GTPase activity (MF)
