# Supplementary material for: Long non-coding RNAs as pan-cancer master gene regulators of associated protein-coding genes: a systems biology approach
Source: PeerJ. 2019 Feb 20;7:e6388. doi: 10.7717/peerj.6388 (PMC6387586; doi:10.7717/peerj.6388)
Supplement: Supplemental Information 1 [file peerj-07-6388-s004.pdf]

Supplementary Information: Supporting Text and References

Long non-coding RNAs as pan-cancer master gene regulators of associated protein-coding genes

A. Saleembhasha and Seema Mishra*

Among our upregulated list of genes, ASF1B (anti-silencing function 1 B histone chaperone) is an isoform of ASF1 and encodes a member of H3 and H4 family of histone chaperone proteins. It is involved in histone deposition, exchange, and removal during nucleosome assembly and disassembly as well as histone modification. ASF1B is implicated as a prognostic value in breast cancer cells, and *ASF1B* mRNA expression level is significantly associated with disease progression, metastasis and poor or shorter survival (1).

CHAF1A (Chromatin Assembly Factor 1 Subunit A) is a primary component of the chromatin assembly factor 1 (CAF1) involved in bringing histones H3 and H4 to replicating DNA and also involved in heterochromatin maintenance (2, 3). CHAF1A inhibits differentiation and promotes an aggressive form of neuroblastoma and elevated levels of CHAF1A strongly correlates with poor prognosis and survival in glioblastoma patients (4). Overexpression of CHAF1A promotes cell proliferation and inhibits apoptosis. CHAF1A is identified as a prognostic biomarker and potential therapeutic target for epithelial ovarian cancer, colon cancer and hepatocellular carcinoma (5, 6, 7).

DNAJC9 (DnaJ Heat Shock Protein Family (Hsp40) Member C9), a novel human type C DnaJ/HSP40 member, contains a typical N-terminal J domain. It can interact with HSP70s through the J domain (N-terminal 98 residues) activating HSP70 ATPase activity (8). The precise role of DNAJC9 in cancer is not investigated.

E2F1 acts as a tumor suppressor in the presence of inactivated RB protein. Dysregulated E2F1 in transformed cells induces apoptosis signaling cascade through activation of an intrinsic p53/p73 cell death pathway. Upon overexpression of E2F1, oncogenic E2F1 target genes, such as EGFR, androgen receptor, EZH2 and CoF are upregulated. Consequent activation of cytoplasmic (PI3K/AKT and RAS/MAPK/ERK) and nuclear signaling cascades related to invasion and metastasis leads to further damages. E2F1 cofactors are also lost which are required for proper activation of apoptotic genes (9). ACTR/AIB1, an E2F1 coactivator, is required for E2F1-mediated gene expression and the consequent

proliferation of ER-negative breast cancer cells (10).

DNA Flap Endonuclease 1 (FEN1) plays a role in both DNA replication and repair thereby maintaining stability and integrity of the genome. Suppression of FEN1 in breast and lung cancers leads to the retardation of DNA replication, DNA double-strand breaks (DSBs) and apoptosis. FEN1 is established as a potential therapeutic treatment for breast cancer and as well as in other cancer types (11, 12).

Forkhead Box M1 (FoxM1) is a transcription factor essential for cell proliferation and cell cycle progression and is associated with proliferation. FOXM1 is involved in tumorigenesis and its elevated levels are associated with tumor progression, invasion and angiogenesis by increasing expression of matrix metalloproteinase-2 (MMP-2), MMP-9 and vascular endothelial growth factor (VEGF) in pancreatic cancer, clear cell renal cell carcinoma; MMP-2 genes in glioma and increased Prostate Specific Antigen gene (PSA-androgen responsive element) transcription in prostate cancer (13, 14, 15, 16). Overexpression of FOXM1 coincides with metastasis of prostate cancer, hepatocellular carcinoma and promotes TGF-β/SMAD3 pathway in breast cancer (17, 18, 19).

CDCA5 (Cell Division Cycle Associated 5) regulates sister chromatid cohesion and proper segregation in mitosis. It is degraded through ubiquitination in G1phase which is anaphase-promoting complex (APC)-dependent (20, 21, 22). CDCA5 is overexpressed and plays a critical role in tumor progression of oral squamous cell carcinoma, urothelial cancer, lung cancer, lung adenocarcinoma (23, 24, 25, 26).

IRAK1 (interleukin-1 receptor-associated kinase) mediates toll-like receptor (TLR) and interleukin-1 receptor (IL1R) signaling process through interacting with MyD88 and subsequent activation and phosphorylation (27). The phosphorylated IRAK1 is released from receptor complex in order to activate NF-κB by binding to TRAF6 (28). Higher levels of IRAK1 lead to cancer progression and is identified as a novel inflammation-related marker and chemoprevention agent in T cell acute lymphoblastic leukemia (IRAK1 inhibition reduced antiapoptotic protein MCL1 stability), melanoma, lung cancer, breast cancer and as an oncogene in hepatocellular carcinoma (29-35).

ILF3 (interleukin enhancer-binding factor or NF90) binds to both DNA and RNA (36). It regulates IL2 and IL3 transcription, MKP-1 mRNA stability by binding to AU-rich 3'-UTR, translation and primary miRNA processing by a variety of protein complexes (37-39). Overexpression of ILF3 is observed in nasopharyngeal carcinoma, non-small cell lung carcinoma, ovarian cancer and breast cancer (40-43). It involved in tumor progression by urokinase-type plasminogen activator (uPA) in aggressive breast cancer (44).

MAD2L1 (Mitotic Arrest Deficient 2 Like 1) is a mitotic spindle assembly checkpoint protein involved in the spindle-kinetochore attachment and it prevents anaphase till there is proper alignment of all chromosomes on metaphase plate (45). Higher expression of MAD2L1 is associated with tumor progression and poor disease survival in breast cancer and lung adenocarcinoma (46, 47).

NOP2 or p120 functions in the cell cycle during tumor proliferation and is identified as a biomarker in lung cancer (48).

NR2C2AP (Nuclear Receptor 2C2 Associated Protein) or TRA16 (TR4 Orphan Receptor-Associated 16 kDa Protein) suppresses NR2C2-mediated transactivation by binding between NR2C2/TR4 and the TR4-response element in target genes (49). TRA16 promotes tumor growth through enhancing ERβ-mediated transcriptional activity by interacting with ERβ and TR2 in non-small cell lung carcinoma (50). Higher expression levels of ORC6L or ORC6 (Origin Recognition Complex Subunit 6) is observed in colorectal cancer; decreased expression of ORC6 induces p21, GADD45beta, reduced JNK1 expression observed in HCT-116 (null-p53) cell and increases chemosensitivity of colon cancer cells to 5-fluorouracil and cisplatin (51).

Upregulated PXNA3 is involved in tumor progression and angiogenesis in breast cancer (52).

Germline mutation in the exonuclease domain of POLE and POLD1 predispose to breast cancer, oligodendroglioma, endometrial cancer and colorectal adenomas (53, 54).

PPIA or CypA (Cyclophilin A) is up-regulated in various cancer types and is associated with malignancy, transformation and metastasis (55-60). Overexpressed PPIA induces tumor growth in small cell lung cancer by activating ERK1/2 signal and resistance to hypoxia- and cisplatin-induced cell death. Knockdown of endogenous CypA disruption of F-actin structure results in decreased anchorage-independent growth, proliferation, and migration in tumor and inhibition (59, 60, 61).

PPP1R14B is significantly overexpressed in ovarian clear cell carcinoma (62).

PTBP1 promotes oncogenesis in breast cancer cell by interacting with PKM2 in anaplastic large cell lymphoma and metastasis in colorectal cancer by negatively regulating ATG10 (63-66).

NR3C2 (Nuclear Receptor Subfamily 3 Group C Member 2) is a receptor for both mineralocorticoids and glucocorticoids, involved in transactivation of target genes. It binds to mineralocorticoid response elements (MRE) (67). Under-expression of NR3C2 is observed in clear cell renal cell carcinoma and in pancreatic cancers by migration inhibitory factor (MIF overexpression observed in pancreatic cancer) through up-regulating miR-301b (68).

Aly/REF Export Factor or THOC4 up-regulation is observed in ovarian cancer and in oral squamous cell carcinoma, it regulates lymph node metastasis by promoting invasiveness and migration (69, 70).

Delta- aminolevulinate dehydratase (ALAD) which is an endogenous inhibitor of the 26S proteasome,

is involved in heme biosynthesis. Down-regulation of delta-aminolevulinate dehydratase is observed in breast cancer and overexpression of ALAD suppresses epithelial-mesenchymal transition by regulating TGF-β (71).

KAT2B/ PCAF a histone acetyltransferase protein acetylates core histones (H3 and H4) and also promotes transcriptional activation by acetylating non-histone proteins. PCAF is induced and activated by ATRA (all-trans-retinoic acid) and PCAF substrates' acetylation promotes granulocytic differentiation in leukemia cells and autophagy by inhibiting Akt/mTOR signaling pathway in hepatocellular carcinoma (72, 73). PCAF blocks up-regulation of TIMP-1 by acetylating STAT3 which modulates crosstalk between tumor cells and CAFs (cancer-associated fibroblasts) in HCC microenvironment (74).

Among our list of lncRNA genes, *PVT1* protects MYC protein, which is a major cancer protein, from phosphorylation-mediated degradation by inhibiting phosphorylation of threonine58 (75). *PVT1* was found to be up-regulated in gastric cancer through *FOXM1* positive feedback loop mechanism involved in growth and invasion (76). From our studies, *PVT1* was found to be up-regulated in all nine types of tumors and *FOXM1* was up-regulated in all 15 types of cancers, providing a major prominent role of these two genes (table 7 and 3). Further, *hPVT1* promotes cell proliferation, cell cycling and acquisition of stem cell-like properties of hepatocellular carcinoma by stabilizing NOP2 proteins (77). Up-regulation of *PVT1* and its splicing variant (PVT1ΔE4) was observed in clear cell renal cell carcinoma (ccRCC) and promoted cell proliferation, invasion and migration through overexpression of BMI1, ZEB1 and ZEB2 (78). Overexpression of *PVT1* is also observed in gastric cancer, showing anti-apoptotic property and upregulating expression of MDR1, MRP, mTOR and HIF-1α (79).

MIR22HG is also known as *C17orf91*. In one study, repression of *C17orf91* impaired migration, invasion and viability of ovarian cancer cells, and downregulated the pro-metastatic gene, MYC, at both mRNA and protein level (80). This lncRNA is seen downregulated across tumor types in our studies.

AC068491.1 (*MIR4435-2* Host Gene or *MIR4435-2 HG*) is found enriched in gastric cancer tissues along with other lncRNAs (*AK001058, INHBA-AS1, UCA1* and *CEBPA-AS1*) and a series of *MIR4435-2HG* fragments observed in the plasma of patients with gastric cancer possess significant differential expression levels (81).

SNHG1 (Small Nucleolar RNA Host Gene 1) is highly expressed in multiple cancer types and is involved in cancer cell growth by regulating gene expression both in cis and in trans. It promotes the transcription of the protein-coding gene SLC3A2 in *cis*; *in trans* form, *SNHG1* upregulates the expression of oncogene MYC by antagonizing FBP-interacting repressor to *FUBP1* (82). *SNHG1* is

predicted as a poor prognostic marker and promotes cell proliferation and invasion, and reduces apoptosis, in hepatocellular carcinoma, glioma, non-small cell lung cancer (NSCLC) and gastric cancer by promoting DNMT1 expression (83-85).

Overexpression of lncRNA *ZNFX1-AS1* o r *ZFAS1* (zinc finger antisense 1) enhances epithelial-mesenchymal transition in glioma and gastric cancer (86, 87). It is also involved in tumor progression, invasion and metastasis in acute myeloid leukemia cell, colorectal cancer by modulating ZEB1, in gastric cancer through exosomes-mediated transfer of *ZFAS1* and in glioma by activation of the Notch signaling pathway (88-92). Over-expression of *ZFAS1* induces hepatocellular carcinoma development by upregulating miR-9 and reducing methylation at CpG island of *miR-9* promoter (93). *ZFAS1* may function as oncogene in colorectal cancer by destabilizing p53 and interacting with CDK1/cyclin B1 complex leading to cell cycle control and inhibition of apoptosis (94). Surprisingly, in a dual action mode, *ZFAS1* is identified as a putative tumor suppressor in breast cancer (95). In epithelial ovarian cancer, *ZFAS1* inhibits proliferation, migration, and chemoresistance by upregulating Sp1 through inhibiting miR-150-5p (96).

LncRNA *GAS5* promotes cell proliferation and inhibits apoptosis in prostate cancer and is identified as a potential diagnostic biomarker and therapeutic target (97). *GAS5* expression was significantly reduced in non-small cell lung cancer, and colorectal cancer; overexpression of *GAS5* inhibits cell proliferation, induces G0/G1 arrest and apoptosis (98-101).

The snoRNA host gene-11 (*SNHG11*) expression was determined in cells exposed to ionizing radiation, *SNHG11* expression was found to be induced in TK6 (p53 positive) and reduced or repressed in WTK1 (p53 negative) cells; this altered expression is part of stress response complex system in radiated cell and its response depends upon p53 function (102).

References:

1. Corpet A, De Koning L, Toedling J (2011). Asf1b, the necessary Asf1 isoform for proliferation, is predictive of outcome in breast cancer. *EMBO J*. 30(3):480-93.
2. Hoek M, Stillman B (2003). Chromatin assembly factor 1 is essential and couples chromatin assembly to DNA replication in vivo. *Proc Natl Acad Sci U S A*. 100(21):12183-8.
3. Reese BE, Bachman KE, Baylin SB, Rountree MR (2003). The methyl-CpG binding protein MBD1 interacts with the p150 subunit of chromatin assembly factor 1. *Mol Cell Biol*. 23(9):3226-36.
4. Barbieri E, De Preter K, Capasso M. (2014). Histone chaperone CHAF1A inhibits differentiation and promotes aggressive neuroblastoma. *Cancer Res*. 74(3):765-74.
5. Xia D, Yang X, Liu W (2017). Over-expression of CHAF1A in Epithelial Ovarian Cancer can promote cell proliferation and inhibit cell apoptosis. *Biochem Biophys Res Commun*. 486(1):191-197.
6. Meng Xu, Yuli Jia, Zhikui Liu (2016). Chromatin assembly factor 1, subunit A (P150) facilitates cell proliferation in human hepatocellular carcinoma. *Onco Targets Ther*. 9: 4023–4035.
7. Wu Z, Cui F, Yu F (2014). Up-regulation of CHAF1A, a poor prognostic factor, facilitates cell proliferation of colon cancer. *Biochem Biophys Res Commun*. 449(2):208-15.
8. Han C, Chen T, Li N, Yang M, Wan T, Cao X. HDJC9 (2007). a novel human type C DnaJ/HSP40 member interacts with and cochaperones HSP70 through the J domain. *Biochem Biophys Res Commun*. 353(2):280-5.
9. Engelmann D, Pützer BM (2012). The dark side of E2F1: in transit beyond apoptosis. *Cancer Res*. 72(3):571-5.
10. Louie MC, Zou JX, Rabinovich A, Chen HW (2004). ACTR/AIB1 functions as an E2F1 coactivator to promote breast cancer cell proliferation and antiestrogen resistance. *Mol Cell Biol*. 24(12):5157-71.
11. He L, Zhang Y, Sun H (2016). Targeting DNA Flap Endonuclease 1 to Impede Breast Cancer Progression. *EBioMedicine*. 2016; 14:32-43.
12. He L, Luo L, Zhu H (2017). FEN1 promotes tumor progression and confers cisplatin resistance in non-small-cell lung cancer. *Mol Oncol*. 2017;11(6):640-654.
13. Wang Z, Banerjee S, Kong D, Li Y, Sarkar FH (2007). Down-regulation of Forkhead Box M1 transcription factor leads to the inhibition of invasion and angiogenesis of pancreatic cancer cells. *Cancer Res*. 67(17):8293-300.

14. Xue YJ, Xiao RH, Long DZ (2012). Overexpression of FoxM1 is associated with tumor progression in patients with clear cell renal cell carcinoma. *J Transl Med*. 10:200.

15. Dai B, Kang SH, Gong W. (2007). Aberrant FoxM1B expression increases matrix metalloproteinase-2 transcription and enhances the invasion of glioma cells. *Oncogene*. 26(42):6212-9.

16. Liu Y, Liu Y, Yuan B (2017). FOXM1 promotes the progression of prostate cancer by regulating PSA gene transcription. *Oncotarget*. 2017; 8(10):17027-17037.

17. Chandran UR, Ma C, Dhir R. (2007). Gene expression profiles of prostate cancer reveal involvement of multiple molecular pathways in the metastatic process. *BMC Cancer*. 7:64.

18. Park HJ, Gusarova G, Wang Z (2011). Deregulation of FoxM1b leads to tumour metastasis. *EMBO Mol Med*. 3(1):21-34.

19. Xue J, Lin X, Chiu WT (2014). Sustained activation of SMAD3/SMAD4 by FOXM1 promotes TGF-β-dependent cancer metastasis. *J Clin Invest*. 124(2):564-79.

20. Zhang N1, Pati D (2012). Sororin is a master regulator of sister chromatid cohesion and separation. *Cell Cycle*. 11(11):2073-83.

21. Schmitz J1, Watrin E, Lénárt P, Mechtler K, Peters JM (2007). Sororin is required for stable binding of cohesin to chromatin and for sister chromatid cohesion in interphase. Curr Biol. 17(7):630-6.

22. Rankin S, Ayad NG, Kirschner MW (2005). Sororin, a substrate of the anaphase-promoting complex, is required for sister chromatid cohesion in vertebrates. *Mol Cell*. 18(2):185-200.

23. Tokuzen N, Nakashiro K, Tanaka H, Iwamoto K, Hamakawa H (2016). Therapeutic potential of targeting cell division cycle associated 5 for oral squamous cell carcinoma. *Oncotarget*. 7(3):2343-53.

24. Chang IW, Lin VC2, He HL (2015). CDCA5 overexpression is an indicator of poor prognosis in patients with urothelial carcinomas of the upper urinary tract and urinary bladder. *Am J Transl Res*. 7(4):710-22.

25. Bidkhori G, Narimani Z, Hosseini Ashtiani S (2013). Reconstruction of an integrated genome-scale co-expression network reveals key modules involved in lung adenocarcinoma. *PLoS One*. 8(7):e67552.

26. Nguyen MH, Koinuma J, Ueda K (2010). Phosphorylation and activation of cell division cycle associated 5 by mitogen-activated protein kinase play a crucial role in human lung carcinogenesis. *Cancer Res*. 70(13):5337-47.

27. Vollmer S, Strickson S, Zhang T (2017). The mechanism of activation of IRAK1 and IRAK4 by interleukin-1 and Toll-like receptor agonists. *Biochem J*. 474(12):2027-2038.

28. Tanishima M, Takashima S, Honda A3 (2017). Identification of optineurin as an interleukin-1 receptor-associated kinase 1-binding protein and its role in regulation of MyD88-dependent signaling. *J Biol Chem*. 292(42):17250-17257.

29. Li Z, Younger K, Gartenhaus R (2015). Inhibition of IRAK1/4 sensitizes T cell acute lymphoblastic leukemia to chemotherapies. *J Clin Invest*. 125(3):1081-97.

30. Srivastava R, Geng D, Liu Y (2012). Augmentation of therapeutic responses in melanoma by inhibition of IRAK-1,-4. *Cancer Res*. 72(23):6209-16.

31. Behrens C, Feng L, Kadara H (2010). Expression of interleukin-1 receptor-associated kinase-1 in non-small cell lung carcinoma and preneoplastic lesions. *Clin Cancer Res*. 16(1):34-44.

32. Wee ZN, Yatim SM1, Kohlbauer VK (2015). IRAK1 is a therapeutic target that drives breast cancer metastasis and resistance to paclitaxel. *Nat Commun*. 6:8746.

33. Scheeren FA, Kuo AH, van Weele LJ (2014). A cell-intrinsic role for TLR2-MYD88 in intestinal and breast epithelia and oncogenesis. *Nat Cell Biol*. 16(12):1238-48.

34. Ye ZH, Gao L, Wen DY, He Y, Pang YY, Chen G (2017). Diagnostic and prognostic roles of IRAK1 in hepatocellular carcinoma tissues: an analysis of immunohistochemistry and RNA-sequencing data from the cancer genome atlas. *Onco Targets Ther*. 10:1711-1723.

35. Ning Li, Jinhua Jiang, Jing Fu (2016). Targeting interleukin-1 receptor-associated kinase 1 for human hepatocellular carcinoma. *J Exp Clin Cancer Res*. 35(1):140.

36. Duchange N, Pidoux J, Camus E, Sauvaget D (2000). Alternative splicing in the human interleukin enhancer binding factor 3 (ILF3) gene. *Gene*. 261(2):345-53.

37. Shi L, Godfrey WR, Lin J, Zhao G, Kao PN (2007). NF90 regulates inducible IL-2 gene expression in T cells. J Exp Med. 204(5):971-7.

38. Kiesler P, Haynes PA, Shi L. et al. (2010). NF45 and NF90 regulate HS4-dependent interleukin-13 transcription in T cells. J Biol Chem. 285(11):8256-67.

39. Kuwano Y, Kim HH, Abdelmohsen K. et al. MKP-1 mRNA stabilization and translational control by RNA-binding proteins HuR and NF90. Mol Cell Biol. 2008; 28(14):4562-75.

40. Guo NL, Wan YW, Tosun K. et al. Confirmation of gene expression-based prediction of survival in non-small cell lung cancer. Clin Cancer Res. 2008; 14(24):8213-20.

41. Dougherty EJ, Elinoff JM, Ferreyra GA. et al. Mineralocorticoid Receptor (MR) trans-Activation of Inflammatory AP-1 Signaling: dependence on DNA sequence, MR conformation, and AP-1 family member expression. J Biol Chem. 2016; 291(45):23628-23644.

42. Guo NL, Wan YW, Tosun K. Confirmation of gene expression-based prediction of survival in non-small cell lung cancer. Clin Cancer Res. 2008;14(24):8213-20.

43. Guo Y, Fu P, Zhu H. Correlations among ERCC1, XPB, UBE2I, EGF, TAL2 and ILF3 revealed by gene signatures of histological subtypes of patients with epithelial ovarian cancer. Oncol Rep. 2012; 27(1):286-92.

44. Hu Q, Lu YY, Noh H, Hong S, Dong Z, Ding HF, Su SB, Huang S. Interleukin enhancer-binding factor 3 promotes breast tumor progression by regulating sustained urokinase-type plasminogen activator expression. Oncogene. 2013; 32(34):3933-43.

45. Li Y, Benezra R. Identification of a human mitotic checkpoint gene: hsMAD2. Science. 1996; 274(5285):246-8.

46. Wang Z, Katsaros D2, Shen Y. et al. Biological and Clinical Significance of MAD2L1 and BUB1, Genes Frequently Appearing in Expression Signatures for Breast Cancer Prognosis. PLoS One. 2015; 10(8):e0136246.

47. Shi YX, Zhu T, Zou T. et al. Prognostic and predictive values of CDK1 and MAD2L1 in lung adenocarcinoma. Oncotarget. 2016; 7(51):85235-85243.

48. Uchiyama B, Saijo Y, Kumano N. et al. Expression of nucleolar protein p120 in human lung cancer: difference in histological types as a marker for proliferation. Clin Cancer Res. 1997; 3(10):1873-7.

49. Yang Y, Wang X, Dong T. et al. dentification of a novel testicular orphan receptor-4 (TR4)-associated protein as repressor for the selective suppression of TR4-mediated transactivation. J Biol Chem. 2003; 278(9):7709-17.

50. Fang F1, Zheng Q, Zhang J (2013). Testicular orphan nuclear receptor 4-associated protein 16 promotes non-small cell lung carcinoma by activating estrogen receptor β and blocking testicular orphan nuclear receptor 2. *Oncol Rep*. 29(1):297-305.

51. Gavin EJ1, Song B, Wang Y, Xi Y, Ju J (2008). Reduction of Orc6 expression sensitizes human colon cancer cells to 5-fluorouracil and cisplatin. *PLoS One*. 3(12):e4054.

52. Gabrovska PN, Smith RA, Tiang T (2011). Semaphorin-plexin signalling genes associated with human breast tumourigenesis. *Gene*. 489(2):63-9.

53. Palles C, Cazier JB, Howarth KM (2013). Germline mutations affecting the proofreading domains of POLE and POLD1 predispose to colorectal adenomas and carcinomas. *Nat Genet*. 45(2):136-44.

54. Bellido F, Pineda M1, Aiza G. et al. POLE and POLD1 mutations in 529 kindred with familial colorectal cancer and/or polyposis: review of reported cases and recommendations for genetic testing and surveillance. Genet Med. 2016; 18(4):325-32.

55. Gong Z, Chi C1, Huang X. et al. Cyclophilin A Is Overexpressed in Hepatocellular Carcinoma and Is Associated with the Cell Cycle. Anticancer Res. 2017; 37(8):4443-4447.

56. Yang H, Chen J, Yang J, Qiao S, Zhao S, Yu L. Cyclophilin A is upregulated in small cell lung cancer and activates ERK1/2 signal. Biochem Biophys Res Commun. 2007; 361(3):763-7.

57. Qi YJ, He QY, Ma YF. Proteomic identification of malignant transformation-related proteins in esophageal squamous cell carcinoma. J Cell Biochem. 2008; 104(5):1625-35.

58. Li Z, Zhao X, Bai S, Wang Z, Chen L, Wei Y, Huang C. Proteomics identification of cyclophilin a as a potential prognostic factor and therapeutic target in endometrial carcinoma. Mol Cell Proteomics. 2008; 7(10):1810-23.

59. Semba S, Huebner K. Protein expression profiling identifies cyclophilin A as a molecular target in Fhit-mediated tumor suppression. Mol Cancer Res. 2006; 4(8):529-38.

60. Choi KJ1, Piao YJ, Lim MJ. et al. Overexpressed cyclophilin A in cancer cells renders resistance to hypoxia- and cisplatin-induced cell death. Cancer Res. 2007; 67(8):3654-62.

61. Calhoun CC1, Lu YC, Song J, Chiu R. Knockdown endogenous CypA with siRNA in U2OS cells results in disruption of F-actin structure and alters tumor phenotype. Mol Cell Biochem. 2009; 320(1-2):35-43.

62. Worley MJ Jr, Liu S, Hua Y. Molecular changes in endometriosis-associated ovarian clear cell carcinoma. Eur J Cancer. 2015; 51(13):1831-42.

63. He X, Arslan AD, Ho TT. Involvement of polypyrimidine tract-binding protein (PTBP1) in maintaining breast cancer cell growth and malignant properties. Oncogenesis. 2014; 3:e84.

64. Hwang SR, Murga-Zamalloa C, Brown N. Pyrimidine tract-binding protein 1 mediates pyruvate kinase M2-dependent phosphorylation of signal transducer and activator of transcription 3 and oncogenesis in anaplastic large cell lymphoma. Lab Invest. 2017; 97(8):962-970.

65. Takahashi H, Nishimura J, Kagawa Y. Significance of Polypyrimidine Tract-Binding Protein 1 Expression in Colorectal Cancer. Mol Cancer Ther. 2015; 14(7):1705-16.

66. Rachidi SM, Qin T, Sun S, Zheng WJ, Li Z. Molecular profiling of multiple human cancers defines an inflammatory cancer-associated molecular pattern and uncovers KPNA2 as a uniform poor prognostic cancer marker. Hum Pathol. 2016; 47(1):45-51.

67. Bhalla S, Chaudhary K, Kumar R. et al. Gene expression-based biomarkers for discriminating early and late stage of clear cell renal cancer. Sci Rep. 2017; 7:44997.

68. Yang S, He P, Wang J. et al. A Novel MIF Signaling Pathway Drives the Malignant Character of Pancreatic Cancer by Targeting NR3C2. Cancer Res. 2016; 76(13):3838-50.

69. Rachidi SM, Qin T, Sun S, Zheng WJ, Li Z (2016). Molecular profiling of multiple human cancers defines an inflammatory cancer-associated molecular pattern and uncovers KPNA2 as a uniform poor prognostic cancer marker. *Hum Pathol*. 47(1):45-51.

70. Saito Y, Kasamatsu A, Yamamoto A (2013). ALY as a potential contributor to metastasis in human oral squamous cell carcinoma. *J Cancer Res Clin Oncol*. 139(4):585-94.

71. Ge J, Yu Y, Xin F. Downregulation of delta-aminolevulinate dehydratase is associated with poor prognosis in patients with breast cancer. Cancer Sci. 2017;108(4):604-611.

72. Sunami Y, Araki M, Kan S. Histone Acetyltransferase p300/CREB-binding Protein-associated Factor (PCAF) Is Required for All-trans-retinoic Acid-induced Granulocytic Differentiation in Leukemia Cells. J Biol Chem. 2017; 292(7):2815-2829.

73. Jia YL, Xu M, Dou CW. P300/CBP-associated factor (PCAF) inhibits the growth of hepatocellular carcinoma by promoting cell autophagy. Cell Death Dis. 2016; 7(10):e2400.

74. Zheng X, Xu M, Yao B. IL-6/STAT3 axis initiated CAFs via up-regulating TIMP-1 which was attenuated by acetylation of STAT3 induced by PCAF in HCC microenvironment. Cell Signal. 2016; 28(9):1314-24.

75. Teresa Colombo, Lorenzo Farina, Giuseppe Macino and Paola Paci (). PVT1: A Rising Star among Oncogenic Long Noncoding RNAs. Biomed Res Int. 2015; 2015: 304208.

76. Xu .M.D, Wang .Y, Weng. W. et al. A Positive Feedback Loop of lncRNA-PVT1 and FOXM1 Facilitates Gastric Cancer Growth and Invasion. Clinical Cancer Research, 2017; 16-0742.

77. Wang F, Yuan JH, Wang SB. et al. Oncofetal long noncoding RNA PVT1 promotes proliferation and stem cell-like property of hepatocellular carcinoma cells by stabilizing NOP2. Hepatology. 2014; 60(4):1278-90.

78. Yang T, Zhou H1,2, Liu P1. lncRNA PVT1 and its splicing variant function as competing endogenous RNA to regulate clear cell renal cell carcinoma progression. Oncotarget. 2017; 8(49):85353-85367.

79. Zhang XW, Bu P, Liu L, Zhang XZ, Li J. Overexpression of long non-coding RNA PVT1 in gastric cancer cells promotes the development of multidrug resistance. Biochem Biophys Res Commun. 2015; 462(3):227-32.

80. Jun Li, Hailin Yu, Meili Xi, and Xin. Long noncoding RNA C17orf91 is a potential prognostic marker and functions as an oncogene in ovarian cancer. J Ovarian Res. 2016; 9: 49.

81. Ke D, Li H, Zhang Y, An Y, Fu H, Fang X, Zheng X. The combination of circulating long noncoding RNAs AK001058, INHBA-AS1, MIR4435-2HG, and CEBPA-AS1 fragments in plasma serve as diagnostic markers for gastric cancer. Oncotarget. 2017; 8(13):21516-21525.

82. Sun Y, Wei G, Luo H, Wu W, Skogerbø G, Luo J and Chen R. The long noncoding RNA SNHG1 promotes tumor growth through regulating transcription of both local and distal genes. Oncogene. 2017; 36(49):6774-6783.

83. Zhang M, Wang W, Li T. et al. Long noncoding RNA SNHG1 predicts a poor prognosis and promotes hepatocellular carcinoma tumorigenesis. Biomed Pharmacother. 2016; 80:73-9.

84. Wang Q, Li Q, Zhou P. et al. Upregulation of the long non-coding RNA SNHG1 predicts poor prognosis, promotes cell proliferation and invasion, and reduces apoptosis in glioma. Biomed Pharmacother. 2017; 91:906-911.

85. Hu Y, Ma Z, He Y, Liu W, Su Y, Tang Z. et al. LncRNA-SNHG1 contributes to gastric cancer cell proliferation by regulating DNMT1. Biochem Biophys Res Commun. 2017; 491(4):926-931.

86. Lv QL, Chen SH, Zhang X. et al. Upregulation of long noncoding RNA zinc finger antisense 1 enhances epithelial-mesenchymal transition in vitro and predicts poor prognosis in glioma. Tumour Biol. 2017; 39(3):1010428317695022.

87. Zhou H, Wang F, Chen H. et al. Increased expression of long-noncoding RNA ZFAS1 is associated with epithelial-mesenchymal transition of gastric cancer. Aging (Albany NY). 2016; 8(9):2023-2038.

88. Guo H, Wu L, Zhao P, Feng A. Overexpression of long non-coding RNA zinc finger antisense 1 in acute myeloid leukemia cell lines influences cell growth and apoptosis. Exp Ther Med. 2017; 14(1):647-651.

89. Fang C, Zan J, Yue B, Liu C, He C, Yan D. Long non-coding ribonucleic acid zinc finger antisense 1 promotes the progression of colonic cancer by modulating ZEB1 expression. J Gastroenterol Hepatol. 2017; 32(6):1204-1211.

90. Wang W, Xing C. Upregulation of long noncoding RNA ZFAS1 predicts poor prognosis and prompts invasion and metastasis in colorectal cancer. Pathol Res Pract. 2016; 212(8):690-5.

91. Pan L, Liang W, Fu M. et al. Exosomes-mediated transfer of long noncoding RNA ZFAS1 promotes gastric cancer progression. J Cancer Res Clin Oncol. 2017; 143(6):991-1004.

92. Gao K, Ji Z, She K, Yang Q, Shao L. Long non-coding RNA ZFAS1 is an unfavourable prognostic factor and promotes glioma cell progression by activation of the Notch signaling pathway. Biomed Pharmacother. 2017; 87:555-560.

93. Wang T, Ma S, Qi X. et al. Long noncoding RNA ZNFX1-AS1 suppresses growth of hepatocellular carcinoma cells by regulating the methylation of miR-9. Onco Targets Ther. 2016; 9:5005-14.

94. Thorenoor N, Faltejskova-Vychytilova P, Hombach S. et al. Long non-coding RNA ZFAS1 interacts with CDK1 and is involved in p53-dependent cell cycle control and apoptosis in colorectal cancer. Oncotarget. 2016; 7(1):622-37.

95. Askarian-Amiri ME, Crawford J, French JD. et al. SNORD-host RNA Zfas1 is a regulator of mammary development and a potential marker for breast cancer. RNA. 2011; 17(5):878-91.

96. Xia B, Hou Y, Chen H, Yang S, Liu T, Lin M, Lou G. Long non-coding RNA ZFAS1 interacts with miR-150-5p to regulate Sp1 expression and ovarian cancer cell malignancy. Oncotarget. 2017; 8(12):19534-19546.

97. Zhang Y, Su X, Kong Z. et al. An androgen reduced transcript of LncRNA GAS5 promoted prostate cancer proliferation. PLoS One. 2017; 12(8):e0182305.

98. Tan Q, Zuo J, Qiu S. Identification of circulating long non-coding RNA GAS5 as a potential biomarker for non-small cell lung cancer diagnosisnon-small cell lung cancer, long non-coding RNA, plasma, GAS5, biomarker. Int J Oncol. 2017; 50(5):1729-1738.

99. Shi X, Sun M, Liu H, Yao Y, Kong R, Chen F, Song Y. A critical role for the long non-coding RNA GAS5 in proliferation and apoptosis in non-small-cell lung cancer. Mol Carcinog. 2015; 54 Suppl 1:E1-E12.

100. Yang Y, Shen Z, Yan Y. Long non-coding RNA GAS5 inhibits cell proliferation, induces G0/G1 arrest and apoptosis, and functions as a prognostic marker in colorectal cancer. Oncol Lett. 2017; 13(5):3151-3158.

101. Ma C, Shi X, Zhu Q, Li Q, Liu Y, Yao Y, Song Y. The growth arrest-specific transcript 5 (GAS5): a pivotal tumor suppressor long noncoding RNA in human cancers. Tumour Biol. 2016; 37(2):1437-44.

102. M. Ahmad Chaudhry (2013). Expression Pattern of Small Nucleolar RNA Host Genes and Long Non-Coding RNA in X-rays-Treated Lymphoblastoid Cells. Int J Mol Sci. 14(5): 9099–9110.
